# Molecular Motors’ Magic Methyl and Its Pivotal Influence on Rotation

**DOI:** 10.1021/jacs.4c01628

**Published:** 2024-04-24

**Authors:** Yohan Gisbert, Maximilian Fellert, Charlotte N. Stindt, Alexander Gerstner, Ben L. Feringa

**Affiliations:** Stratingh Institute for Chemistry, University of Groningen, Nijenborgh 4, 9747 AG Groningen, The Netherlands

## Abstract

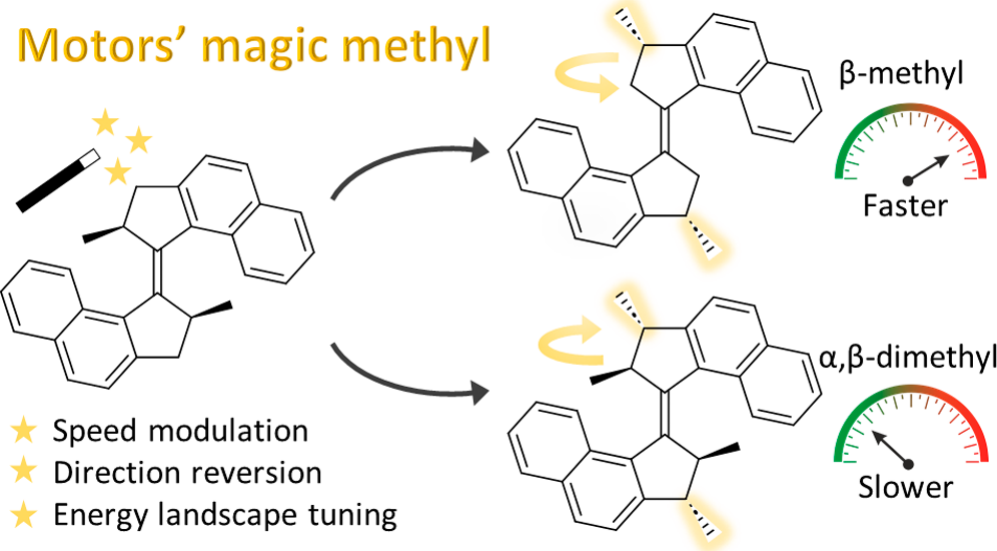

Molecular motors have found a wide range of applications, powering a transition from molecules to dynamic molecular systems for which their motion must be precisely tuned. To achieve this adjustment, strategies involving laborious changes in their design are often used. Herein, we show that control over a single methyl group allows a drastic change in rotational properties. In this regard, we present the straightforward asymmetric synthesis of β-methylated first-generation overcrowded-alkene-based molecular motors. Both enantiomers of the new motors were prepared in good yields and high enantiopurities, and these motors were thoroughly studied by variable-temperature nuclear magnetic resonance (VT–NMR), ultraviolet–visible (UV–vis), and circular dichroism (CD) spectroscopy, showing a crucial influence of the methylation pattern on the rotational behavior of the motors. Starting from a common chiral precursor, we demonstrate that subsequent methylation can drastically reduce the speed of the motor and reverse the direction of the rotation. We show for the first time that complete unidirectionality can be achieved even when the energy difference between the stable and metastable states is small, resulting in the coexistence of both states under ambient conditions without hampering the energy ratcheting process. This discovery opens the way for the design of more advanced first-generation motors.

## Introduction

Since the discovery of artificial light-driven molecular motors^1,2^ in 1999,^3^ there has been tremendous progress in controlling the rotary motion,^4,5^ understanding the key principles of their functioning,^6^ and fine-tuning their properties. Among other artificial molecular machines^7−14^ that have been used as pumps,^15−18^ muscles,^19−22^ shuttles,^23−25^ and transporters,^26,27^ molecular motors have been applied in various fields of chemistry,^28,29^ in materials,^30,31^ including metal–organic frameworks (MOFs), covalent organic frameworks (COFs),^32−34^ liquid crystals (LCs),^35−37^ and for drug delivery.^38^

**Scheme 1:**
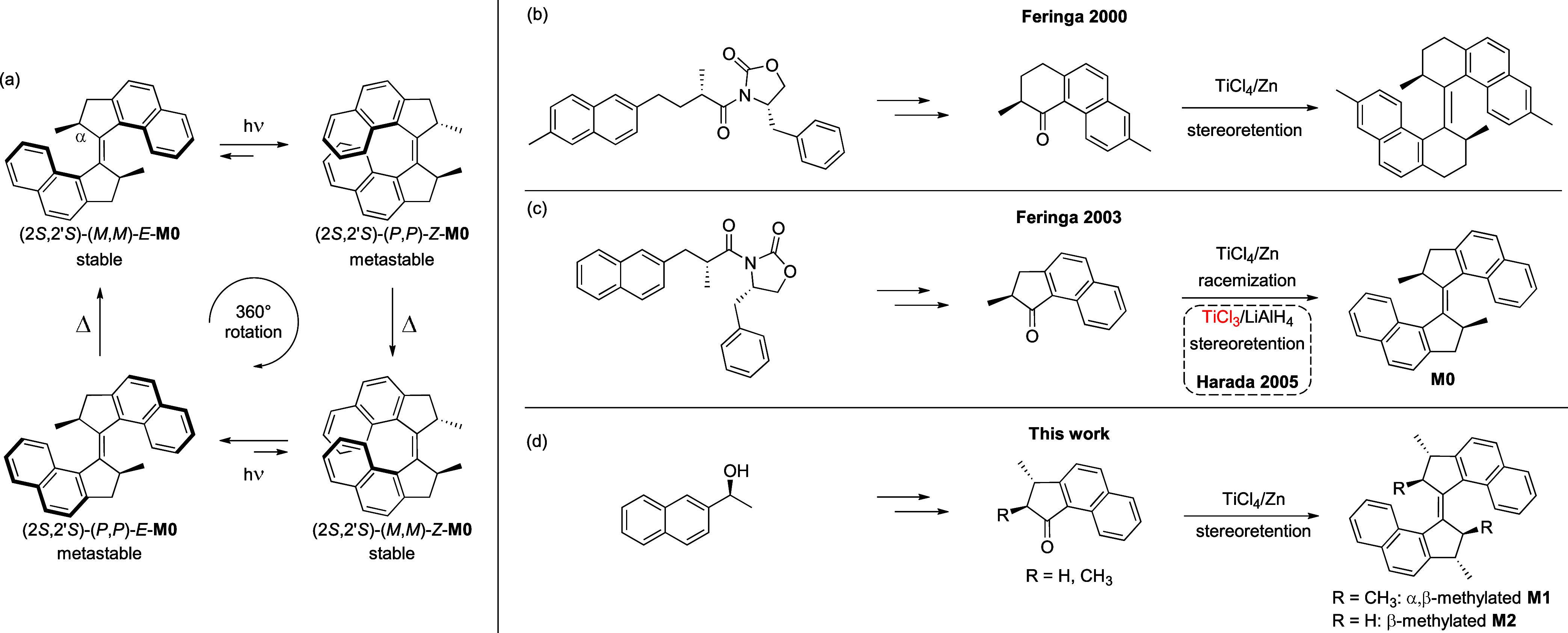
(a) Unidirectional Rotation Cycle of Molecular Motor M0.^49^ (b–d) Strategies for the Asymmetric Synthesis of First-Generation Molecular Motors

First-generation molecular motors are based on symmetric overcrowded alkenes^39,40^ comprising two point chiral stereogenic centers in proximity to the central alkene double bond functioning as the rotation axle. Due to the steric bulk around this double bond, the molecule is forced to adopt a helical shape. The interplay between the two stereochemical elements–the point chirality and the helical chirality–enables unidirectional rotation of the molecular motor through a four-step cycle (Scheme 1a) composed of successive photoisomerizations of the central double bond and thermal helix inversions (THIs).^3^ It has been shown that the choice of the substituent at the stereogenic center allows for a fine-tuning of the rotational properties, by changing the steric hindrance within the fjord region.^41,42^ This change affects the energy differences between stable and metastable states of *Z* and *E* isomers, governing the nature of the corresponding equilibria and resulting in the modulation of key features such as the rotational speed and directionality. Furthermore, the configuration at the stereocenters defines the direction of the rotation. A sample of the enantiopure molecular motor will thus show rotation in a single direction, whereas the overall directionality of a population composed of a racemic motor will be null, even if each individual motor molecule shows unidirectional rotary motion. Controlling the direction of the rotation via a well-defined stereocenter is undeniably essential for many applications of the molecular motors, ranging from collective behavior in materials such as LCs^43^ to multistage chiral catalysts.^44,45^

Therefore, achieving access to enantiopure molecular motors has been a topic of great interest over the past two decades.^46^ The methods employed to obtain enantioenriched molecular motors can be divided into three main categories:^2,47^ chiral chromatographic separation,^48,49^ direct asymmetric synthesis,^49−53^ including transition-metal catalyzed domino cyclization strategies,^54,55^ and the use of chiral auxiliaries.^56^ Aside from the direct resolution of specifically functionalized derivatives,^57^ there have been few asymmetric synthesis strategies reported for first-generation motors. Early approaches by our group involved the use of Evans’ auxiliary for the asymmetric synthesis of motor precursors (Scheme 1b,c).^49,52^ Subsequent dimerization under standard McMurry conditions successfully afforded first-generation motors featuring six-membered rings on both halves (Scheme 1b), but their five-membered analogues racemized under these conditions (Scheme 1c).^49^ It was later shown by Harada et al.^58^ that this racemization process could be circumvented by replacing TiCl_4_/Zn with TiCl_3_/LiAlH_4_ in the McMurry coupling step affording the enantiopure product, albeit in low yields (Scheme 1c).^50^ More recently, the asymmetric synthesis of smaller indanone-based upper halves was shortened by employing an expensive Au-catalyzed enantioselective protonation, followed by McMurry coupling (TiCl_3_/Zn).^50^ Nevertheless, these McMurry conditions involve the use of sensitive TiCl_3_, which has very limited commercial availability. Facing the previously cited stereochemical issues and aiming for a more practical and scalable approach, we developed an alternative strategy relying on the asymmetric synthesis of α,β-dimethylated ketones for which epimerization under the usual McMurry conditions is disfavored by a privileged *trans* configuration of the methyl groups, analogously to a pathway reported for second-generation motors preventing racemization during the Barton–Kellogg coupling methodology (Scheme 1d).^53^

This new method provides access to a novel class of β-methylated molecular motors distinct from the previously developed α-methylated first-generation molecular motors.^59^ This raises a key fundamental question about the possibility of driving the unidirectional rotation of first-generation motors with remote β-methyl substituents. Here, we thoroughly study the influence of the methylation pattern on the rotational characteristics of a series of enantiopure first-generation molecular motors and show that minor structural changes can result in radically different properties. The methyl group at the stereogenic center plays a predominant role in the functioning of the overcrowded-alkene-based motors. Changing its configuration induces an inversion of the helicity of the molecule resulting in an opposite direction of the rotation.^60^ Modification of its position significantly influences the energy landscape of the rotation cycle, altering both the directionality and the rotation frequency of the motor.^61^ Such behavior is reminiscent of the concept of “magic methyl”, widely used in medicinal chemistry to illustrate the profound effect of the addition, stereochemistry, or change of position of a single methyl group on the pharmacological effect of a drug.^62,63^ These findings shed new light on the design rules governing the properties of light-driven molecular rotary motors.

## Results and Discussion

### Asymmetric Synthesis

**Scheme 2:**
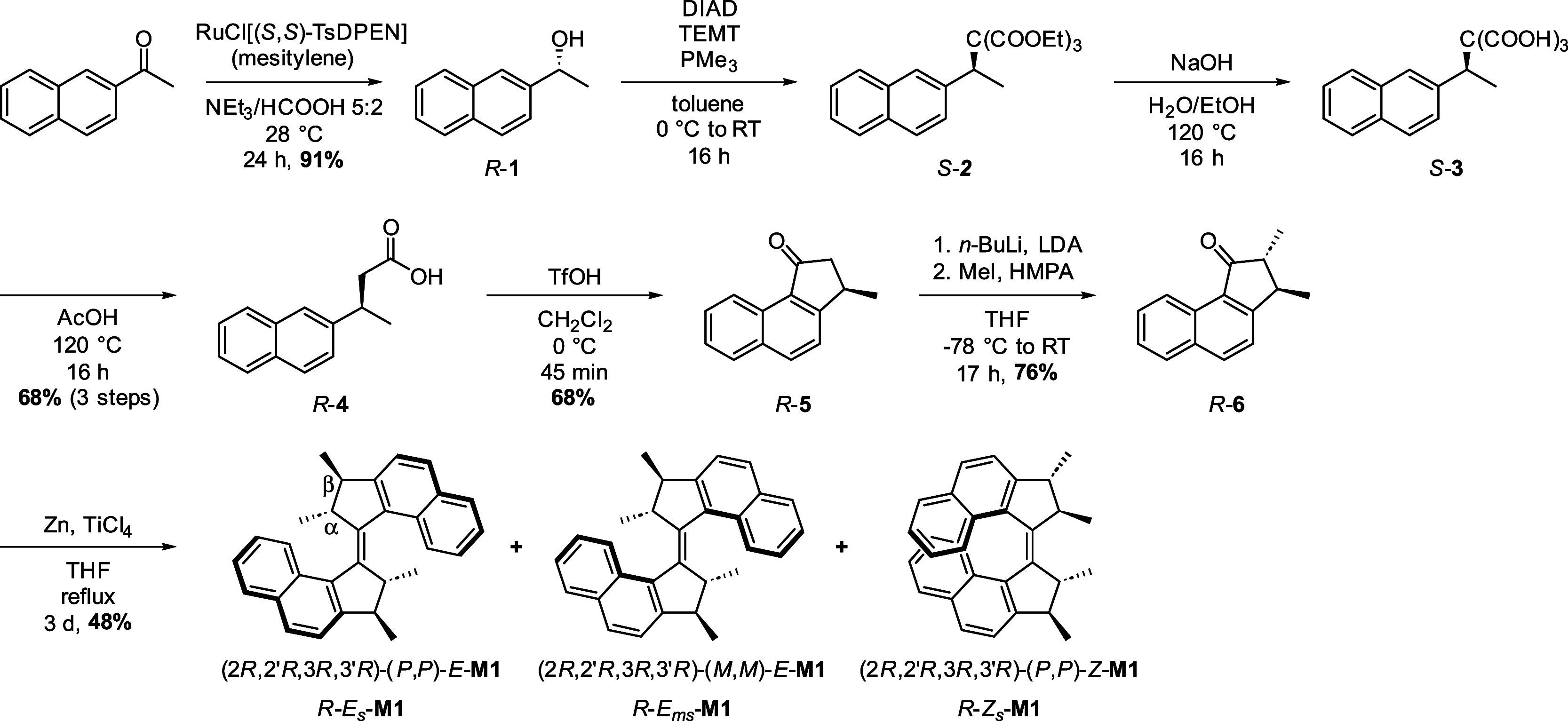
Asymmetric Synthesis of Motor *R*-**M1** *E*/*Z* ratios were found to be varying depending on the handling conditions. *E*_s_/*E*_ms_ ratio is 72:28 at 20 °C.

As a general asymmetric synthetic strategy to access both enantiomers, the previously reported pathway for the synthesis of the extended indanone *S-***6**(53) was modified for the preparation of *R-***6** by introducing a catalytic enantioselective reduction step, early in the sequence, allowing to obtain both benzyl alcohol precursors *R*-**1** and *S*-**1** (Scheme 2). Specifically, *R***-1** was synthesized by a Ru-catalyzed Noyori-type asymmetric transfer hydrogenation^64^ in good yield (91%) and with high enantiopurity (ee = 95%) starting from readily available and inexpensive 2-acetonaphthone. Unlike the previously reported methods, we were able to access both enantiomers from the same widely available precursor. Nonreported ketones *R***-5** and *R*-**6** were then prepared according to our previously described method (Scheme 2):^53^ Starting from *R***-1**, *R***-4** was obtained in a three-step sequence involving a Mitsunobu reaction with triethylmethanetricarboxylate (TEMT) as the nucleophile, followed by saponification and decarboxylation. Subsequent TfOH-mediated ring closing afforded β-methylated ketone *R***-5**, which could be further converted into the α,β-dimethylated ketone *R-***6** by reacting the in situ formed lithium enolate with MeI. Enantiomeric ketones *S-***5** and *S-***6** were prepared using the same synthetic sequence.

Molecular motor **M1** was obtained by dimerization of *R-***6** via McMurry coupling, affording a mixture of *R-E-***M1** and *R-Z-***M1** in varying ratios in 48% yield (Scheme 2). Pure *R-E-***M1** isomer could be isolated by selective precipitation from methanol. After this precipitation step, an enantiomeric excess of ≥99% was determined for the purified *R*-*E*-**M1** isomer, underlining that the McMurry coupling with TiCl_4_/Zn did not result in epimerization. Spectroscopic analysis of the purified *E* isomer of the overcrowded alkene revealed that it consisted of a mixture of two conformers: the stable (*R-E*_s_*-***M1**) and metastable (*R-E*_ms_*-***M1**) isomers in a 72:28 ratio at 20 °C.

**Figure 1:**
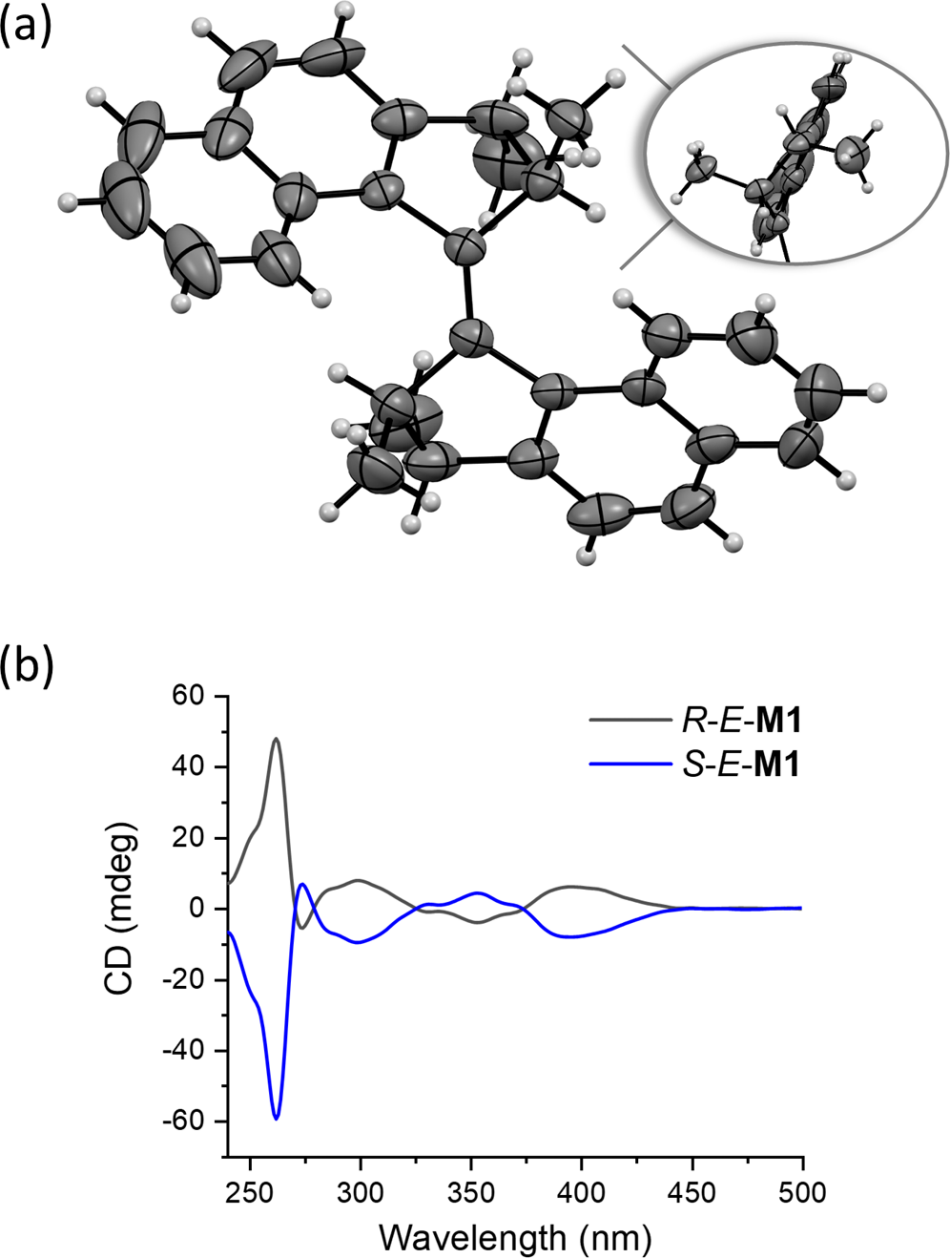
(a) X-ray structure of *R*-*E*_s_-**M1** and side view of the aliphatic region of one half of motor **M1**. Thermal ellipsoids are drawn at a 50% probability. (b) CD spectra of *R*-*E*-**M1** (black) and *S*-*E*-**M1** (blue) in CH_2_Cl_2_ at 20 °C. Both samples are composed of a mixture of stable and metastable states *E*_s_-M1/*E*_ms_-**M1** in a 72:28 ratio.

Single crystals of *R*-*E*_s_-**M1** suitable for X-ray diffraction (XRD) were obtained by the slow evaporation of a concentrated CH_2_Cl_2_/methanol solution of *R*-**M1**. The obtained crystal structure confirmed the expected structure of the overcrowded alkene (Figure 1a), featuring all methyl substituents in a pseudoaxial conformation and (*P*,*P*)-helicity. The enantiomer of the motor (*S-E-***M1**) was prepared in an analogous way by dimerization of the α,β-dimethylated ketone *S***-6**, leading to a comparable yield (46%) and enantiopurity (ee ≥ 99%). Figure 1b shows the circular dichroism (CD) spectra obtained for *R*-*E*-**M1** and *S-E-***M1**, and as expected, both enantiomers display opposite Cotton effects.

### Experimental Demonstration of the Unidirectional Rotation of **M1**

Molecular motor **M1** was originally envisioned as an analogue of our previously reported motor **M0** (Scheme 1),^49,58,65−67^ featuring two adjacent methyl substituents at each rotor unit and similar properties but accessed through asymmetric synthesis. However, we discovered that the presence of an extra methyl group at the β-position with respect to the double bond significantly influenced the relative stability of the isomers composing the four-step rotation cycle of molecular motor **M1**. Indeed, density functional theory (DFT) calculations showed that compared with its α-methylated counterpart **M0**, **M1** displays smaller energy differences between the stable and metastable forms of both *Z* and *E* isomers (Figure S33). This distinction is particularly pronounced for the *E* isomer, for which the stable state was calculated to be only 1.4 kJ/mol lower in energy than the metastable form for α,β-dimethylated **M1**, while this difference was calculated to be 4.4 kJ/mol for reported α-methylated analogue **M0**. Experimentally, this small difference in energy resulted in an equilibrium of both the stable state of the *E* isomer (*E*_s_-**M1**) and the metastable state (*E*_ms_-**M1**) in a ratio of 72:28 in fully relaxed samples at room temperature.

**Figure 2:**
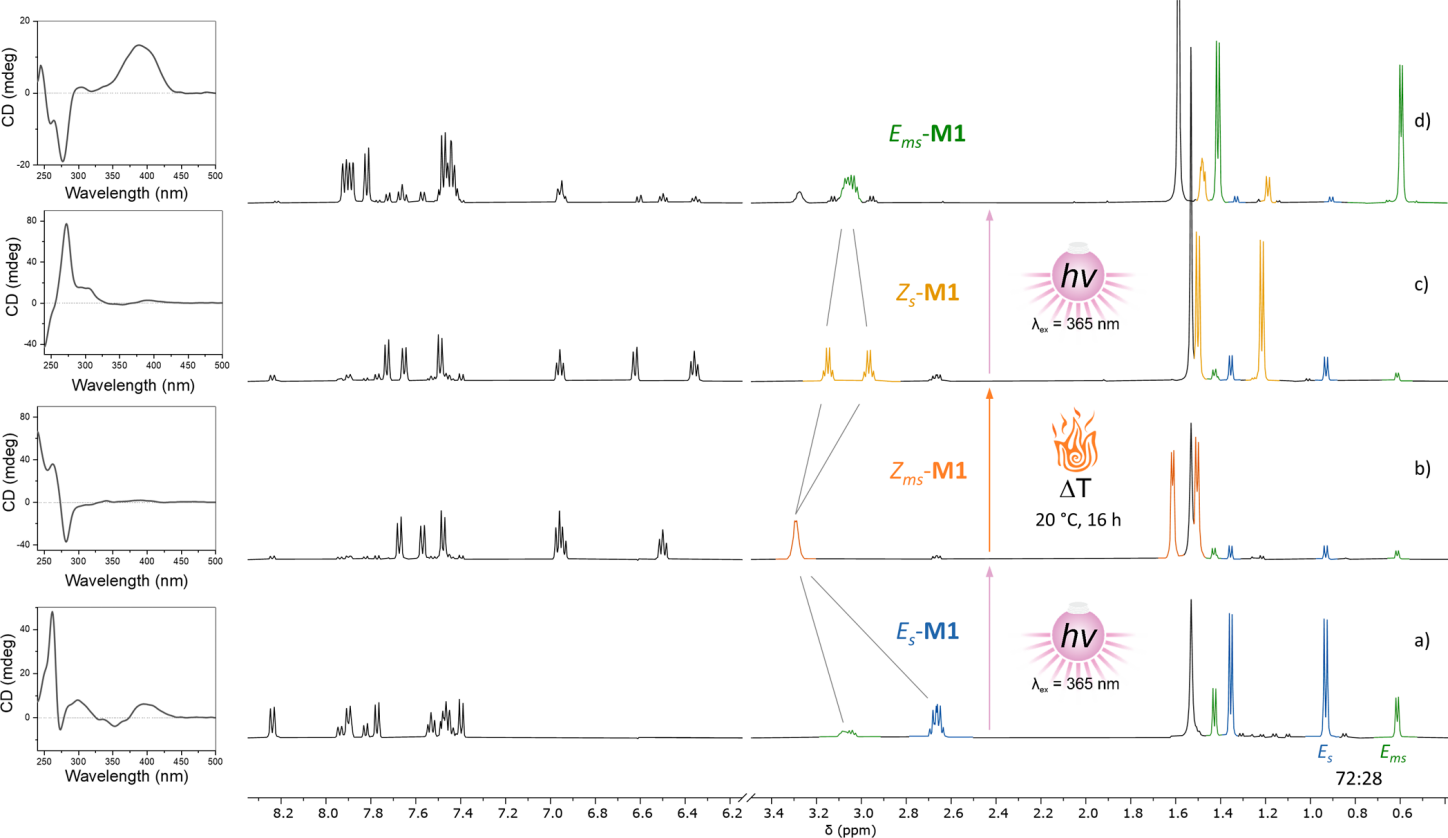
Rotational cycle of **M1** monitored by CD spectroscopy (*R*-**M1**, CH_2_Cl_2_, ∼ 30 μM) and variable temperature ^1^H NMR spectroscopy (500 MHz, CH_2_Cl_2_). (a) Initial sample of pure stable *E*_s_-**M1** at 20 °C, (b) PSS reached after in situ irradiation with 365 nm light showing mainly *Z*_ms_-**M1** (20 °C), (c) mixture obtained after complete relaxation at 20 °C over 16 h composed of a majority of *Z*_s_-**M1**, and (d) PSS reached after in situ irradiation with 365 nm light showing a majority of *E*_ms_-**M1** (^1^H NMR at −10 °C, CD at −20 °C).

It is generally assumed that such a small energy difference, resulting in a thermal equilibrium of stable and metastable states, is detrimental to the directionality efficiency of the rotation mechanism of a molecular motor, even though a bias on a single THI can result in overall directionality. Indeed, it is often inferred that upon irradiation, photoisomerization of both species would be observed, resulting in simultaneous rotation in the desired direction (conversion of *E*_s_ to *Z*_ms_) and in the opposite way (*E*_ms_ to *Z*_s_) inducing a loss of overall directionality.^53,60,61^ In sharp contrast, we discovered that irradiation of samples of *E*-**M1**, composed of stable and metastable states in a 72:28 ratio (Figure 2a) with 365 nm light at room temperature afforded only the *Z*_ms_-**M1** isomer while the intensity of the signals corresponding to both *E*_ms_-**M1** and *E*_s_-**M1** decreased (84:16 *Z*_ms_/*E*_s+ms_ ratio at PSS). Z_ms_-**M1** could be easily identified by ^1^H NMR with characteristic methyl doublets at 1.50 and 1.61 ppm and a single signal for the α-methyl protons at 3.29 ppm (Figure 2b). This observation of an apparently fully unidirectional process disagrees with generally accepted design rules of overcrowded-alkene-based molecular motors,^2^ which led us to investigate the mechanism of this step in more detail. In situ NMR irradiation of a sample of *E*-**M1** at −30 °C resulted in a PSS with a 6:94 ratio between *E*_s_-**M1** and *Z*_ms_-**M1**, while no formation of the *Z*_s_-**M1** state by photoisomerization of the *E*_ms_-**M1** state was observed (Figure S6). Notably, at this temperature, both THI processes were inhibited, and the concentration of *E*_ms_-**M1** remained constant, thus confirming the complete unidirectionality of this step. Interestingly, a separate experiment showed that a nonquantitative PSS ratio (*Z*_s_-**M1**/*E*_ms_-**M1** = 15:85) was reached when *Z*_s_-**M1** was converted to *E*_ms_-**M1** by irradiation at the same wavelength (Figure 2d), meaning that a photoequilibrium between *Z*_s_-**M1** and *E*_ms_-**M1** exists and should result in the backswitching of ca. 15% of *E*_ms_-**M1** to *Z*_s_-**M1** when the initial mixture of *E* isomers is irradiated at low temperature. Nevertheless, this behavior, which would decrease the directionality efficiency, was not observed. This result suggests that the directionality of this first step arises from an interplay of thermal and photochemical ratcheting processes, with *E*_s_-**M1** being converted significantly faster than *E*_ms_-**M1** upon irradiation. Valuable insights into the mechanism of this process can be obtained by comparison of **M1** to **M0** (our original first-generation rotary molecular motor) for which an in-depth photochemical study was reported by Sension et al.^65^ In this work, the authors demonstrated that the conversion of *E*_ms_-**M0** into *Z*_s_-**M0** has a low quantum yield (Φ = 0.08) compared to the conversion of *Z*_s_-**M0** into *E*_ms_-**M0** (Φ = 0.85) and *E*_s_-**M0** into *Z*_ms_-**M0** (Φ = 0.85) having both very high quantum yields, thus explaining the faster conversion of *E*_s_ compared to *E*_ms_ upon irradiation. The authors showed that this asymmetry is due to different profiles of the excited-state landscapes for the forward and backward reactions and the possible involvement of two distinct conical intersections. This result also sheds light on the mechanism of the switching of both states of the *E* isomer to *Z*_ms_-**M1** at room temperature. Although photoswitching apparently takes place only between *E*_s_-**M1** and *Z*_ms_-**M1**, *E*_ms_-**M1** is also depleted because it is in thermal equilibrium with *E*_s_-**M1** at a temperature where the THI process is relatively fast. The overall process at 20 °C was also monitored by ultraviolet–visible (UV–vis) spectroscopy, showing a bathochromic shift of the maximum absorption wavelength (λ_max_) from 374 to 398 nm (Figure S24), consistent with the formation of a metastable state. Under the same conditions, CD spectroscopy displayed significant changes, including the formation of two new bands with strong intensities at 282 and around 240 nm (Figure 2b).

The thermal helix inversion from *Z*_ms_-**M1** to *Z*_s_-**M1** took place at 20 °C over ca. 14 h. This process was monitored by ^1^H NMR spectroscopy, showing the decrease of intensity of a doublet corresponding to methyl protons at 1.61 ppm and the simultaneous increase of a new doublet at 1.22 ppm, as well as the splitting of the broad signal at 3.29 ppm corresponding to both α-methyl protons in two well-defined quartets at 2.97 and 3.15 ppm (Figure 2c). As expected for the THI of a first-generation molecular motor, a hypsochromic shift of λ_max_ to 375 nm was observed by UV–vis spectroscopy (Figure S25). CD spectroscopy showed a sign inversion of the Cotton effect at 240 and 273 nm (Figure 2c), confirming the change in helicity during the THI step.

To experimentally evaluate the difference in the speed and thermodynamic parameters of **M1** compared with its reported counterpart **M0** (Scheme 1), the thermal relaxation process was monitored at five different temperatures by ^1^H NMR spectroscopy. This series of experiments allowed us to determine the Gibbs free energy of activation of the THI at 20 °C (Δ*G*_**M1***-Z*-THI_^‡^ = 94.5 ± 0.5 kJ/mol) by means of Eyring analysis, corresponding to a half-life time of *t*_1/2-**M1**-*Z*-THI_ = 2.1 h at the same temperature (Figure S16). This free energy value is similar to the one reported in the same solvent for **M0** (Δ*G*_**M0***-Z*-THI_^‡^ = 94.2 kJ/mol), resulting in a half-life time *t*_1/2-**M0**-*Z*-THI_ = 1.9 h at 20 °C.^58^

The *Z*_s_-**M1** state was then converted to the *E*_ms_-**M1** state by irradiation with 365 nm light. In situ ^1^H NMR spectroscopy irradiation experiments at −10 °C showed the upfield shift of both characteristic doublets corresponding to the methyl protons of *Z*_s_-**M1** at 1.19 and 1.48 ppm to two new doublets at 0.59 and 1.41 ppm (Figure 2d). As discussed before, a PSS ratio of 15:85 (*Z*_s_-**M1**/*E*_*m*s_-**M1**) was observed. UV–vis spectroscopy at 0 °C displayed a bathochromic shift of the λ_max_ from 375 to 393 nm (Figure S26), similar to what was observed for the *E*_s_-**M1** to *Z*_ms_-**M1** switching process. CD spectroscopy at −20 °C showed significant changes, consistent with a helix inversion including sign inversions of the Cotton effects at 245 and 278 nm and a strong increase in intensity of the band at 388 nm (Figure 2d). At these temperatures, no relaxation to the *E*_s_-**M1** isomer through the THI took place on the short time scale of the irradiation.

Monitoring of the subsequent THI process was performed by ^1^H NMR spectroscopy at 5 °C, showing regeneration of the initial *E*_s_-**M1** state after a complete unidirectional rotation cycle (Figure S5). In line with the observation of an equilibrium between the *E*_s_-**M1** and *E*_ms_-**M1** states in the initial sample, the THI process afforded a mixture of both isomers with a 72:28 ratio at 20 °C. Both CD and UV–vis spectroscopy showed regeneration of the spectral features of the initial sample of the *E* isomer. Eyring analysis of this THI process allowed us to determine the Gibbs free energy of activation at 20 °C of Δ*G*_**M1***-E*-THI_^‡^ = 85.0 ± 0.4 kJ/mol corresponding to a half-life time of 2.6 min at the same temperature (Figure S17). These values are significantly higher than the ones reported for the α-methylated derivative **M0** (Δ*G*_**M0***-E*-THI_^‡^ = 80.4 kJ/mol) for which the half-life time was found to be only 24 s (0.4 min) at 20 °C.^49^

Thus, **M1** performs a fully unidirectional rotary motion upon irradiation at room temperature, as both steps display perfect directionality efficiencies, and can be employed as an attractive alternative to the already reported **M0**, as both enantiomers of **M1** are readily accessible in good yields and with high enantiopurity (>99%) by a scalable asymmetric synthesis. The use of dimethylated halves significantly reduced the energy difference between the stable and metastable forms of the *E* isomer, resulting in an equilibrium of both states after complete relaxation. Nevertheless, we have shown that this feature did not result in a loss of directionality of the motor, in contrast with what was inferred in previous studies. Inspired by this observation, we set out to explore new first-generation molecular motors that lack a stereocenter in the α-position.

There are only a few examples of second-generation molecular motors bearing only a stereocenter in the β-position, which were mainly explored to reverse the direction of rotary motion.^42,53,60,61^ However, they all feature small energy differences between the stable and metastable states, which resulted in a hampering of the overall unidirectionality of the rotation, thus resulting in a lower efficiency. To our knowledge, no such example of a β-methylated first-generation motor has been studied. As we demonstrated that a small energy difference between stable and metastable states did not result in a loss of unidirectionality for our α,β-dimethylated motor **M1**, we designed **M2**, a less hindered motor featuring mono-β-methylated halves.

### Preparation and Study of the Unidirectional Rotation of **M2**

**Scheme 3:**
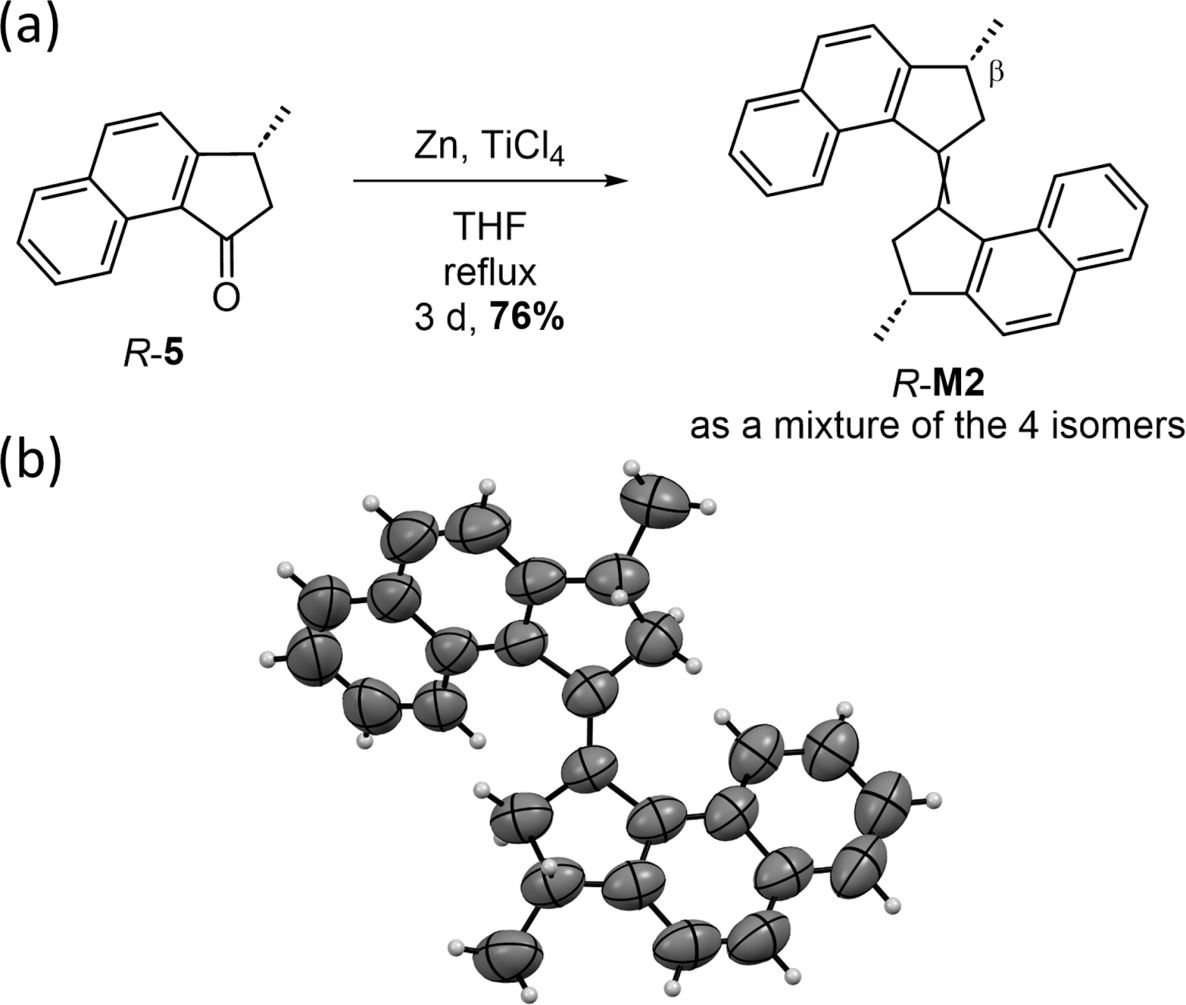
(a) Asymmetric Synthesis of Motor *R*-**M2**, (b) X-ray Structure of *R*-*E*_s_-**M2**. Thermal Ellipsoids Are Drawn at 50% Probability

The corresponding enantioenriched motor *R*-**M2** was obtained by direct McMurry coupling of mono-β-methylated ketone *R*-**5** in a good yield of 76% (Scheme 3a). The *E* isomer *R*-*E*-**M2** was isolated with a high enantiopurity after precipitation from methanol (ee = 94%), whereas *S*-**M2** was obtained in comparable yield (72%) and enantiopurity (ee = 98%) using the same procedure but employing ketone *S*-**5** as the precursor.

**Figure 3:**
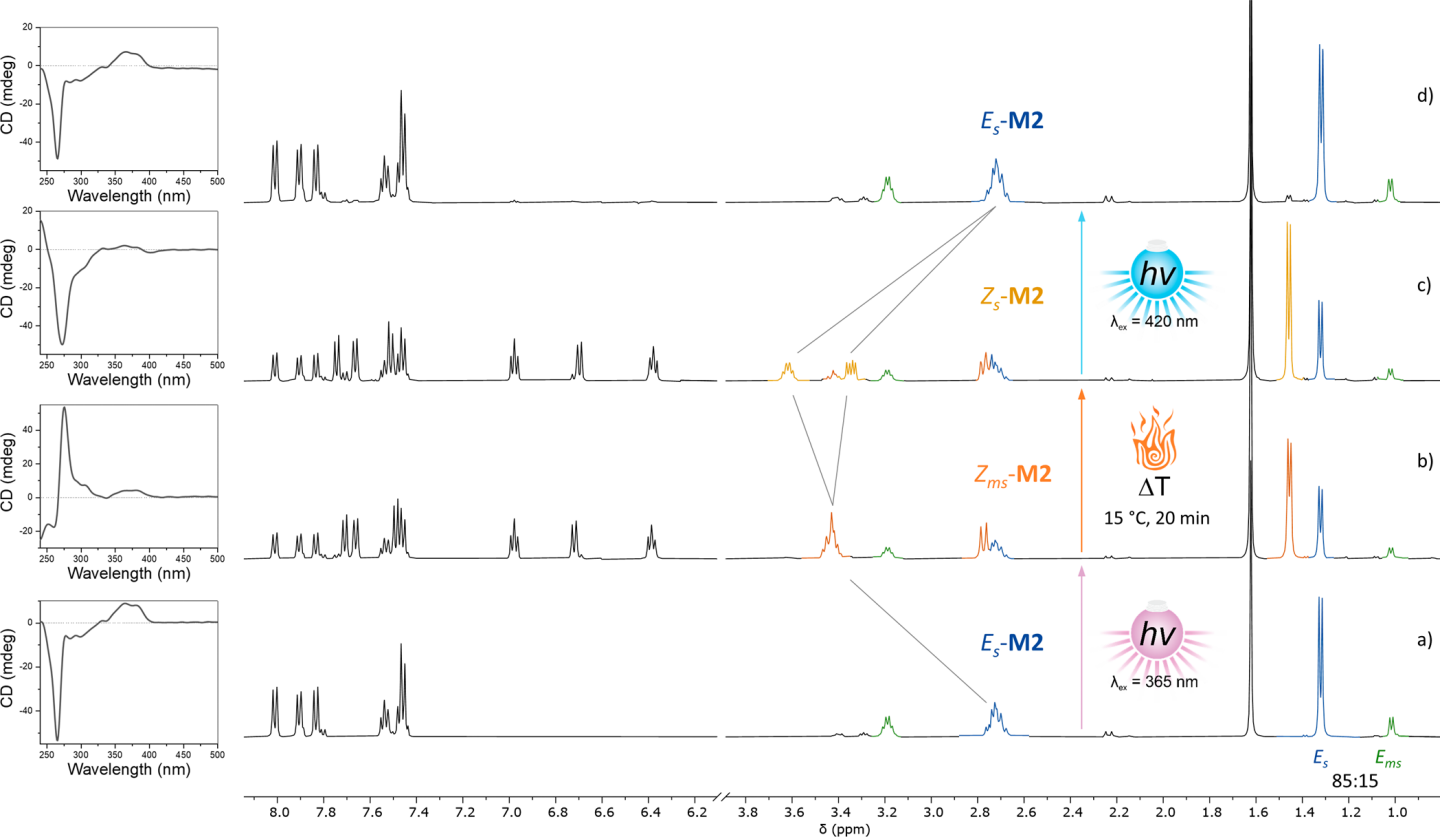
Full rotational cycle of **M2** monitored by CD spectroscopy (*R*-**M2**, CH_2_Cl_2_, ∼30 μM, −20 °C) and ^1^H NMR spectroscopy (500 MHz, CH_2_Cl_2_, −30 °C). (a) Initial sample of *E*_s_-**M2**, (b) PSS reached after in situ irradiation with 365 nm light showing conversion to metastable *Z*_ms_-**M2**, (c) mixture obtained after complete relaxation at 15 °C over 20 min composed of a majority of *Z*_s_-**M2**, and (d) *E*_s_-**M2** recovered after a complete rotation cycle.

^1^H NMR spectroscopy confirmed the presence of two isomers, stable *E*_*s*_-**M2** and metastable *E*_ms_-**M2** (Figure 3a). These two isomers were found to be in an intermediate exchange regime on the NMR time scale at 20 °C, resulting in broad aliphatic signals. Cooling the sample to −30 °C slowed the exchange and resulted in the splitting and sharpening of the aliphatic signals (Figure 3a). Well-resolved doublets were observed for *E*_s_-**M2** (1.32 ppm) and for *E*_ms_-**M2** (1.02 ppm) in an 85:15 ratio at −30 °C in CH_2_Cl_2_.

In situ ^1^H NMR irradiation at −30 °C with 365 nm light resulted in the partial disappearance of both *E*_s_-**M2** and *E*_ms_-**M2** and the formation of a single new species, metastable *Z*_ms_-**M2**, displaying a characteristic doublet at 1.46 ppm arising from the methyl groups. Upon irradiation, a steady state composed of these three species (*Z*_ms_/*E*_s_/*E*_ms_) in a 61:33:6 ratio was reached (Figure 3b). No formation of *Z*_s_-**M2** by the photoisomerization of *E*_ms_-**M2** was observed. This suggests a mechanism similar to that observed for **M1**, involving a faster photoisomerization of *E*_s_-**M2** to *Z*_ms_-**M2** and a simultaneous conversion of *E*_ms_-**M2** to *E*_s_-**M2** by equilibration through the thermally favored THI process. UV–vis spectroscopy of this mixture at 20 °C showed a decreasing intensity of the band at λ_max_ = 367 nm and the formation of a redshifted shoulder to this band at λ ≈ 415 nm upon irradiation with 365 nm light (Figure S28). CD spectroscopy displayed a sign inversion and a slight shift (266–276 nm) of the most intense Cotton effect (Figure 3b), in line with the expected inversion of helicity induced by the photoisomerization process.

Increase in the temperature to 15 °C allowed the thermally induced helix inversion to take place, resulting in the complete relaxation of metastable *Z*_ms_-**M2** to *Z*_s_-**M2** (Figure 3c). Due to the small energy difference between *Z*_ms_-**M2** and *Z*_s_-**M2**, this thermal relaxation did not lead to the complete disappearance of *Z*_ms_-**M2** but afforded a mixture of the two species in a ratio of 83:17 (*Z*_s_-**M2**/*Z*_ms_-**M2**). This thermal decay was monitored by ^1^H NMR spectroscopy at five different temperatures, which allowed us to determine an energy barrier for this THI process of Δ*G*_**M2***-Z*-THI_^‡^ = 84.8 ± 0.7 kJ/mol at 20 °C, corresponding to a half-life time of 2.4 min at the same temperature (Figure S18). Therefore, the slowest helix inversion of **M2** is remarkably faster than the one of **M1** for which a half-life time of 2.1 h was determined, highlighting that the removal of the methyl groups in the α positions resulted in a 50× increase in the rotational speed for this THI step. The crowding in the Fjord region of overcrowded-alkene-based molecular motors has indeed been shown to have a strong influence on their rotational speed.^68,69^

Although this transformation was associated with almost negligible changes in the UV–vis spectrum at 0 or 20 °C (Figure S29), it resulted in a sign inversion of the most intense Cotton effect at 276 nm as observed by CD spectroscopy (Figure 3c).

When attempting irradiation at −30 °C using various wavelengths, it was found that *Z*_s_-**M2** could selectively be isomerized to *E*_s_-**M2** (after a rapid thermal decay of the initially formed *E*_ms_-**M2**) with 420 nm light, while the generated *E*_s_ state remained unchanged (no formation of *Z*_ms_-**M2** was observed). The PSS_420_ reached after irradiation is composed of *E*_s_-**M2** and *E*_ms_-**M2** (82 and 14% respectively), being in thermal equilibrium, and ca. 4% of residual *Z*_ms_-**M2** from the almost quantitative PSS ratio. This ratio indicates that upon irradiation at 420 nm, part of *Z*_ms_-**M2** (originally ca. 10% of the global composition) was photoisomerized to *E*_s_-**M2**, implying that 6% of the overall population rotated in the opposite direction (i.e., decreased directionality efficiency). This phenomenon is consistent with the common behavior of first-generation molecular motors undergoing back-isomerization at longer wavelengths.^58^

This process was further studied at −90 °C, where the thermal decay of *E*_ms_-**M2** to *E*_s_-**M2** was prevented, indeed showing the conversion of *Z*_s_-**M2** to *E*_ms_-**M2** while a part of *Z*_ms_-**M2** was converted to *E*_s_-**M2** by rotation in the opposite direction (Figure S11). While irradiation with 365 nm light proved inefficient under these conditions, it was found that using 395 nm light at −90 °C allowed us to selectively convert *Z*_s_-**M2** to *E*_ms_-**M2**, while the concentration of *Z*_ms_-**M2** remained unchanged, thus providing complete overall unidirectionality (Figure S12), hence inducing rotation of **M2** with a perfect directionality efficiency. Under these conditions, the obtained PSS_395_ (31% of residual *Z* isomer) is significantly lower than PSS_420_ (4–5% of the *Z* isomer). Therefore, the use of 420 nm light was preferred to study the intermediates of the rotation cycle, while 395 nm is more appropriate for discussing the autonomous fully unidirectional rotation of the motor.

The formation of *E*_ms_-**M2** was also supported by low-temperature CD spectroscopy showing a sign inversion and a slight shift of the most intense band at 268 nm (Figure S32) after irradiation with 420 nm light at −90 °C, as expected from a photoinduced helicity inversion.

The decay of *E*_ms_-**M2** to *E*_s_-**M2** was studied by ^1^H NMR spectroscopy by successive irradiation at −90 °C and thermal relaxation at −75 °C (Figure S14). However, as *E*_ms_-**M2** and *E*_s_-**M2** are in constant exchange under experimentally less demanding cryogenic conditions via the same transition state, we determined the associated energy barrier by variable-temperature exchange spectroscopy (VT-EXSY, Figures S17–S21): Δ*G*_**M1***-E*-THI_^‡^ = 60.1 ± 0.6 kJ/mol, corresponding to a half-life time of 6 ms, both at 20 °C.

To study the unidirectional rotation cycle described above, two different wavelengths were used for photoisomerizations: 365 nm for the conversion of *E*_s_-**M2** to *Z*_ms_-**M2** and 420 or 395 nm for the switching of *Z*_s_-**M2** to *E*_s_-**M2**. Even though dual-wavelength irradiation of **M2** can be achieved experimentally to obtain autonomous unidirectional rotation of the motor, using a single wavelength can be desirable for experimental convenience. Hence, it was shown that a single wavelength of 395 nm can be used instead to achieve both photochemical steps involved in the rotational cycle simultaneously upon irradiation, which results in a fully autonomous rotation under ambient conditions with near-visible light (Figure S15).

**Figure 4:**
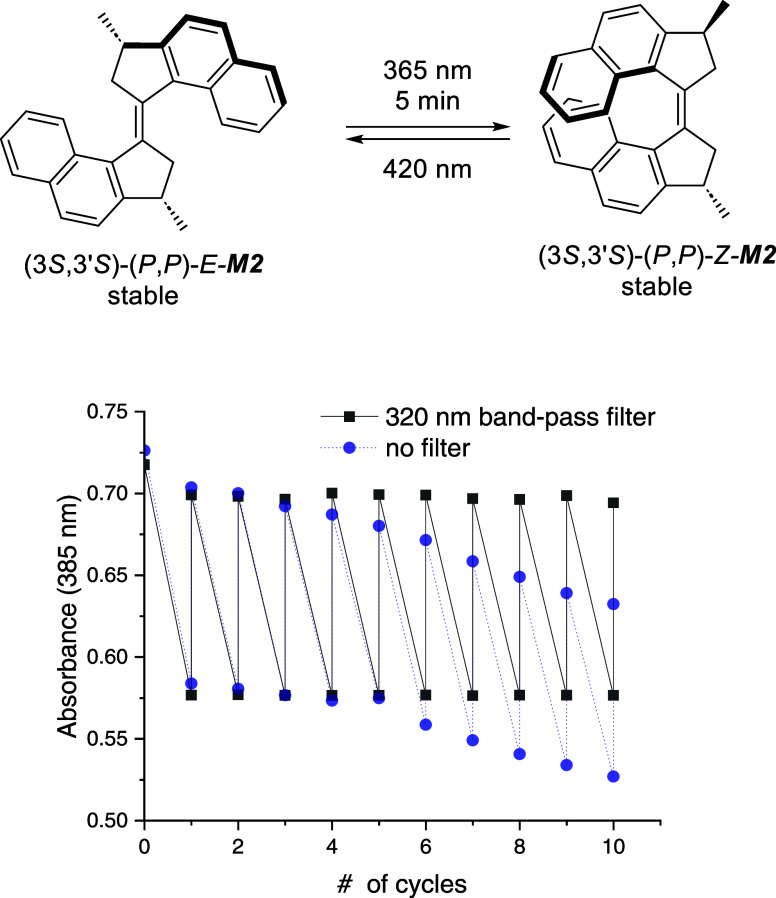
Fatigue study of **M2**, measured by UV–vis spectroscopy (CH_2_Cl_2_, ∼30 μM, 20 °C). Changes in absorbance at 385 nm were monitored upon irradiating with 365 nm light, waiting for ca. 5 min, recording a spectrum, irradiating with 420 nm light recording a spectrum, and repeating the cycle (experiment with and without a band-pass filter).

Because of its relatively high rotation speed and the possibility to regenerate the initial states almost quantitatively upon irradiation with 365 and 420 nm light, we envisioned that **M2** could be used as an efficient chiroptical switch under ambient conditions, with apparent bistable behavior if the temperature is high enough. Indeed, at room temperature, **M2** could reliably be switched between two states, one containing a majority of *E*_s_-**M2**, in exchange with *E*_ms_**-M2** that can be readily converted to *Z*_s_-**M2** upon irradiation with 365 nm light followed by a fast thermal relaxation of the obtained *Z*_ms_-**M2** (less than 5 min at 20 °C) to *Z*_s_-**M2**, constituting the second state. The initial *E*_s_-**M2** could be regenerated by irradiation with 420 nm light. A fatigue study of this switching process (Figure 4) was performed using UV–vis spectroscopy under the same conditions as before. The switching system proved to be very robust and demonstrated excellent photostability and reversibility at the studied wavelengths, with no loss in absorbance observed over ten switching/backswitching cycles when a 320 nm band-pass filter was used to block the rather intense low-wavelength light from the spectrophotometer detector lamp. In this way, UV–vis and CD spectra can be switched between the two previously described states with, respectively, a majority of *E*_s_-**M2** with a λ_max_ of 367 nm and a main Cotton effect resulting in a narrow band at 266 nm and a second state featuring a majority of *Z*_s_-**M2** displaying a λ_max_ of 381 nm and a broader Cotton effect with a maximum at 274 nm.

### Theoretical and Structural Analysis

DFT calculations were also performed to further investigate and compare the rotation mechanisms of **M0**, **M1**, and **M2**. Geometries of the isomers and transition states involved in the rotational cycles were computed at the r^2^SCAN-3c^70^ level of theory using the conductor-like polarizable continuum CPCM(CH_2_Cl_2_) solvent model.^71^ As expected from the experimental results discussed above, very similar energy profiles were calculated for **M0** and **M1** except a smaller energy difference between the *E*_s_ and *E*_ms_ states of **M1** (1.4 kJ/mol for **M1** vs 4.4 kJ/mol for **M0**), in line with the observed equilibrium of *E*_s_-**M1** and *E*_ms_-**M1**, whereas the isolation of pure *E*_s_-**M0** was previously reported. In contrast, the rotation mechanism calculated for **M2** features a flatter energy landscape with small energy differences among both pairs of metastable and stable isomers (2.5 kJ/mol between *Z*_ms_-**M2** and *Z*_s_-**M2** and 2.4 kJ/mol difference for *E*_ms_-**M2** and *E*_s_-**M2**). The energy barriers associated with the THI processes of **M2** are also computed to be smaller than the ones obtained for **M0** and **M1**, as observed experimentally. This difference in behavior can be rationalized by a smaller steric hindrance within the fjord region of **M2**. The calculated energy barriers for the transition states of all three motors are in good agreement with the experimental values determined by Eyring analysis (Table 1).

**Table 1:** Calculated Gibbs Free Energy Values at 20 °C for Stable and Metastable States of Molecular Motors **M0**–**2** and Corresponding THI Barriers, Compared with Experimental Values (kJ/mol)

	**M0**	**M1**	**M2**
*Z*_s_	0	0	0
*Z*_ms_	23.8	22.7	2.5
*E*_s_	19.8	21.7	1.3
*E*_ms_	24.2	23.1	3.7
TS(*Z*-THI)_calc_	97.2	99.7	92.5
TS(*E*-THI)_calc_	87.1	86.4	62.6
TS(*Z*-THI)_exp_	94.2^a^ (93 ± 1^b^)	94.5 ± 0.5	84.8 ± 0.7
TS(*E*-THI)_exp_	80.4^a^ (80 ± 1^b^)	85.0 ± 0.4	60.1 ± 0.6

a Experimental values reported for the THI barriers of **M0** in CD_2_Cl_2_.^58^

b Values in *n*-hexane.^49^

**Scheme 4:**
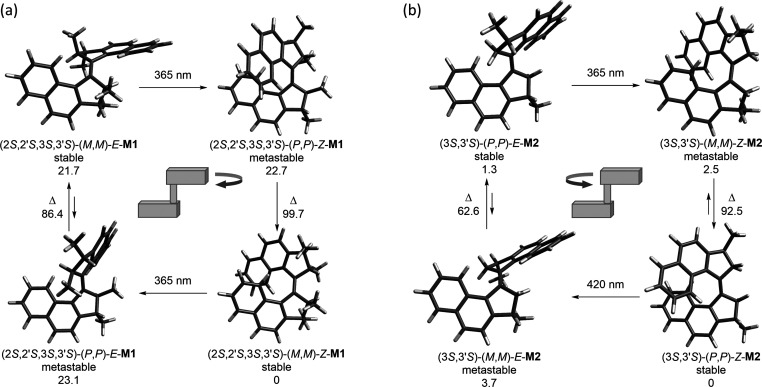
Calculated Geometries and Energies (kJ/mol) of the Intermediates Involved in the Rotational Cycles of (a) *S*-**M1** and (b) *S*-**M2** Both motors originate from the same precursor *S*-**5** but display opposite rotation directions.

The optimized geometries obtained from these calculations also provided insight into the structural differences induced by the absence of methyl groups at the α position of the overcrowded alkene within **M2** compared with those of **M0** and **M1**. It was found that despite originating from the same enantioenriched extended indanone (3*S*)-**5**, (2*S*,2′*S*,3*S*,3′*S*)-**M1** and (3*S*,3′*S*)-**M2** display opposite helicities at every step of the rotation cycle. For instance, both stable *E* and *Z* forms of (3*S*,3′*S*)-**M2** feature (*P*,*P*)-helicities, whereas the stable isomers of (2*S*,2′*S*,3*S*,3′*S*)-**M1** have (*M*,*M*)-helicities (identical to (2*S*,2′*S*)-**M0**), as shown in Scheme 4. The DFT-optimized geometries also show that the methyl groups of **M1** are in a pseudoaxial conformation in the stable states and pseudoequatorial conformation in the metastable states, just as in the reported **M0** and, more generally, in all α-methylated first- and second-generation molecular motors.^2,40^ The opposite behavior is observed for β-methylated **M2**, with methyl groups displaying a pseudoequatorial conformation in the stable states and being pseudoaxial for the metastable isomers (Figure S34).

These geometrical differences were also observed in the crystalline state, as shown by the X-ray structures of *R*-*E*-**M1** (Figure 1) and *R*-*E*-**M2** (Scheme 3b). Both structures correspond to the calculated lowest energy *E* form (i.e., *E*_s_) and feature opposite helicities and opposite conformations (pseudoaxial/equatorial) of the stereogenic methyl groups. CD spectra also give additional information about the opposite helicities of **M1** and **M2** along the rotation cycle (Figures S29–S30), with opposite signs of the most intense Cotton effects (in the 250–300 nm region) for **M1** and **M2** resulting from the same enantiopure ketone (identical stereodescriptors for 3,3′-positions).

The stereochemical implications of the β-methylation (**M2**) compared with the α,β-methylation (**M1**) of this first-generation molecular motor scaffold are therefore much more substantial than initially foreseen, resulting in an opposite helical handedness induced by the same initially installed point chirality at the α-methylated stereocenter.

Remarkably, this phenomenon results in an opposite direction of the rotation for **M2** compared with **M1** despite arising from the same homochiral ketone. For instance, *S*-**M1** and *S*-**M2**, originating from chiral ketone *S*-**5** rotate in opposite directions (Scheme 4). Indeed, the position of the stereogenic methyl group has a crucial influence on directionality and can even lead to its reversal.

## Conclusions

In conclusion, we developed a scalable method for the asymmetric synthesis of first-generation overcrowded-alkene-based molecular motors allowing access to both enantiomers with excellent enantiopurities. Employing this synthetic strategy allowed for the study of the influence of the methylation pattern of molecular motors on their core properties, such as their ability to rotate in a specific unidirectional sense and their speed of rotation. The newly synthesized α,β-dimethylated motor **M1** displayed a small energy difference between stable and metastable isomers which are in thermal equilibrium. Therefore, both states are always present as a mixture of isomers under ambient conditions. Unlike previously inferred in the literature, we demonstrated that this small energy difference does not necessarily result in a loss of directionality, as **M1** provided a fully unidirectional rotation. We showed that this unidirectionality arises from an interplay of photochemical and thermal ratcheting processes, and expect that these new insights will inspire the design of all-photochemical molecular motors based on similar scaffolds.^5,72,73^ This phenomenon was further explored with the preparation and study of the unprecedented β-methylated first-generation molecular motor **M2**. This less-hindered motor was found to be ca. 50 times faster than its α-methylated analogue **M0** and to be fully unidirectional, which thus far had not been observed in molecular motors lacking a substituent at the α-position. We also showed that **M2** rotates in the opposite direction compared with its α,β-dimethylated analogue, **M1**, originating from the same enantioenriched ketone precursor. Therefore, we demonstrated that a slight tuning of the first-generation overcrowded-alkene-based molecular motors’ “magic methyl” not only influences the speed of rotation of the motors but also results in a drastic change in the observed equilibria between the isomers and even induces an inversion of the rotation direction. We anticipate that these fundamental insights will facilitate the future design of novel molecular-motor-based systems.

## Supporting Information Available

Experimental procedures and characterization data, photochemical and kinetic data including UV/vis absorption and NMR spectra and associated kinetic analyses, X-ray crystal structures, and computational details; (PDF)
XYZ coordinates of the structures calculated by DFT (TXT)
