# Supplementary material for: Molecular Motors’ Magic Methyl and Its Pivotal Influence on Rotation
Source: J Am Chem Soc. 2024 Apr 24;146(18):12609–19. doi: 10.1021/jacs.4c01628 (PMC11082891; doi:10.1021/jacs.4c01628)
Supplement: Supplementary file 1 — ja4c01628_si_001.pdf [file ja4c01628_si_001.pdf]

Supporting Information

Molecular motors' magic methyl and its pivotal influence on rotation

Yohan Gisbert,† Maximilian Fellert,† Charlotte N. Stindt, Alexander Gerstner, Ben L. Feringa*
†: equal contributions
Stratingh Institute for Chemistry, University of Groningen, Nijenborgh 4, 9747 AG Groningen (The Netherlands)

Table of contents

1. Supplementary experimental procedures 2
   1.1. General methods 2
   1.2. Computational methods 3
   1.3. X-ray analysis 3
   1.4. Synthetic procedures 3
2. In-situ NMR irradiation and relaxation experiments 12
   2.1. ^{1}H NMR study of M1 12
   2.2. ^{1}H NMR study of M2 15
3. Eyring Analysis 21
   3.1. Eyring analysis: *Z*-M1 21
   3.2. Eyring analysis: *E*-M1 22
   3.3. Eyring analysis: *Z*-M2 23
4. Exchange spectroscopy (VT-EXSY) 24
   4.1. Theoretical model 24
   4.2. Exchange spectroscopy 25
5. Variable-temperature UV-vis spectroscopy 29
   5.1. UV-vis study of M1 29
   5.2. UV-vis study of M2 31
6. Variable-temperature circular dichroism 32
   6.1. CD study of M1 32
   6.2. CD study of M2 33
7. Computational analysis 34
8. X-ray structural data 35
9. HPLC chromatograms 38
10. NMR spectra of new compounds 45
11. References 65

1. Supplementary experimental procedures

1.1. General methods

Commercial reagents and solvents: All chemicals and solvents were purchased from commercial suppliers unless otherwise stated. Anhydrous solvents were obtained using a MBraun SPS 800 system and stored under N_2.

Synthesized reagents: *R*-**1**, *S*-**1**[1] and *R*-**2**, *R*-**3**, *S*-**4**, *S*-**5**, *S*-**6**[2] were synthesized according to reported literature procedures and characterized using routine characterization techniques.

Synthesis and purification: Standard Schlenk techniques were used, employing nitrogen or argon as the inert gas. If not performed at room temperature, the reaction temperatures refer to the temperature of the heating/cooling bath or heating block.

Flash column chromatography was performed on a Biotage Selekt system using the indicated solvents. TLC analysis was done on Merck silica gel 60 F_{254} aluminum sheets, and compounds were visualized with a UV lamp (254 nm or 365 nm).

Analysis:

NMR:

Full characterization of the newly synthesized compounds (including ^{1}H, ^{13}C, and 2D NMR experiments) was performed using a Bruker Avance Neo 600 (600 MHz) spectrometer. Chemical shifts (δ) are given in parts per million (ppm) relative to TMS, using the solvent residual peak as internal standard ($CDCl_3$: δ = 7.26 for ^{1}H, δ = 77.16 for ^{13}C; CD_2Cl_2: δ = 5.32 for ^{1}H, δ = 53.84 for ^{13}C). Data is reported as follows: chemical shifts (δ) in ppm, multiplicity (s = singlet, d = doublet, dd = doublet of doublets, ddd = doublet of doublets of doublets, td = triplet of doublets, t = triplet, q = quartet, br. = broad, m = multiplet), coupling constants *J* (Hz), and integration. Signals were assigned with the help of 2D NMR experiments. Variable temperature NMR and in-situ irradiation experiments were performed using a Varian Inova 500 (500 MHz) spectrometer. NMR irradiation experiments were performed at the indicated temperature with a fiber-coupled LED and a 1500 µm optical fiber (FT1500UMT) to guide the light directly into the NMR tube inside the NMR spectrometer. Relaxation experiments at various temperatures were performed by equilibrating the sample at the given temperature, tuning, locking, and shimming the NMR, irradiating until a stationary state was reached, stopping the irradiation, waiting until the lock signal stabilized and finally starting to record the decay.

High-resolution mass (HMRS) spectra were recorded on a Thermofisher LTQ Orbitrap XL.

HPLC analysis was performed using a Shimadzu SPD M10AVP diode array detector using Chiralcel columns with mixtures of HPLC-grade *n*-heptane and 2-propanol as the eluent and a column temperature of 40 °C.

Optical rotations were measured on a Schmidt + Haensch Polartronic MH8 polarimeter in a 10 cm cell at 20 °C and a wavelength of 589 nm. The samples were measured in triplicates and the average value was used. The concentration c is given in g/100 mL.

UV-vis absorption spectra were recorded on a Agilent Cary 8454 spectrophotometer in 1 cm quartz cuvettes.

CD spectra were recorded on a Jasco J-715 spectropolarimeter. The LEDs were attached via a 1500 µm optical fiber (M93L01).

Low temperature spectroscopic experiments were performed using an Unisoku Cryostat (CoolSpek) which was coupled to the spectrophotometer or spectropolarimeter.

Irradiation experiments were performed using fiber-coupled LEDs (M365F1, M420F1) powered with a T-Cube™ LEDD18 driver obtained from Thorlabs Inc.

1.2. Computational methods

All calculations were performed using the Orca 5.0.4 package.[3] Geometries were optimized with the composite functional r^{2}SCAN-3c,[4] using the conductor-like polarizable continuum CPCM(CH_2Cl_2) solvent model.[5] The thermochemical data were calculated at 20 °C at the same level of theory. The minima and transition states had no or one imaginary frequency, respectively.

1.3. X-ray analysis

Motors (2R,2'R,3R,3'R)-(P,P)-*E*-**M1** and (3R,3'R)-(M,M)-*E*-**M2** were crystallized by slow evaporation of a concentrated solution of the compounds in methanol/CH_2Cl_2. A single crystal was mounted on a cryoloop and analyzed on a Bruker-AXS D8 Venture diffractometer. The data collection was done at room temperature under ambient conditions. The Bruker APEX4 software suite was used for data collection and processing, and the structure was solved using SHELXT.[6] Refinement was performed using SHELXL[7] in the OLEX2 software package.[8]

1.4. Synthetic procedures

(*R*)-1-(naphthalen-2-yl)ethan-1-ol (*R*-1)

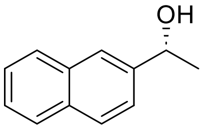

Triethylamine (26.9 mL, 0.194 mol, 2.2 eq.) was added dropwise to formic acid (18.3 mL, 0.485 mol, 5.5 eq.) at 0 °C and the mixture was degassed with N_2 for 10 min. RuCl[(*R,R*)-TsDPEN](mesitylene) (274 mg, 0.440 mmol, 5.0 mol%) and 2-acetonaphthone (15.0 g, 88.1 mmol, 1.0 eq.) were then added under a nitrogen flow. The reaction mixture was stirred at 28 °C for 24 h, quenched with water (50 mL) and the aqueous phase was extracted with EtOAc (3 x 50 mL). The combined organic layers were washed with a saturated aqueous solution of $NaHCO_3$ (50 mL), brine (50 mL), dried over $MgSO_4$ and the solvent was removed *in vacuo*. The crude product was filtered over a plug of silica and eluted with CH_2Cl_2 to afford *R*-**1** as an off-white solid (13.8 g, 80.2 mmol, 91%).

^{1}H NMR (600 MHz, $CDCl_3$, 25 °C): δ = 7.89 – 7.79 (m, 4H), 7.55 – 7.42 (m, 3H), 5.07 (q, *J* = 6.5 Hz, 1H), 1.95 – 1.84 (m, 1H), 1.59 (d, *J* = 6.4 Hz, 3H) ppm.
HPLC (Chiralcel OB-H, *n*-heptane/2-propanol 3:97.5, 1.0 mL/min): retention times (min) 15.00 (*S*-**1**, minor), 17.05 (*R*-**1**, major), *ee* = 95%.

The NMR data match those reported in the literature.[1]

(*R*)-3-(naphthalen-2-yl)butanoic acid (*R*-4)

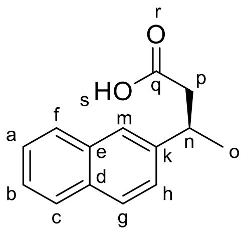

R-**1** (4.50 g, 26.1 mmol, 1.0 eq.) was dissolved in anhydrous toluene (45 mL) and cooled to 0 °C. Trimethylphosphine (1 M in toluene, 52.3 mL, 52.3 mmol, 2.0 eq.), diisopropyl azodicarboxylate (10.3 mL, 52.3 mmol, 2.0 eq.) and triethyl methanetricarboxylate (11.1 mL, 52.2 mmol, 2.0 eq.) were added dropwise at 0 °C and the reaction mixture was stirred for 16 h at room temperature. EtOAc (80 mL) was added to the orange solution and the organic layer was washed with water (150 mL), 1 M aqueous HCl (150 mL) and brine (150 mL). The combined organic layers were dried over Na_2SO_4 and concentrated *in vacuo*. The obtained crude triester *S*-**2** was dissolved in EtOH (40 mL), a 6 M aqueous NaOH (180 mL) was added, and the reaction mixture was stirred at 120 °C for 16 h. EtOAc (90 mL) was then added, and the mixture was acidified with 1 M aqueous HCl. The aqueous phase was extracted with EtOAc (2 x 90 mL) and the combined organic layers were washed with brine (150 mL), dried over Na_2SO_4 and concentrated *in vacuo*. The obtained crude tri-acid *S*-**3** was dissolved in AcOH (150 mL) and the mixture was stirred at 120 °C for 16 h. The reaction mixture was concentrated *in vacuo* and the crude product was purified by column chromatography (SiO_2, pentane/EtOAc, 85:15) to afford *R*-**4** as a colorless solid (3.85 g, 18.0 mmol, 68% over three steps).

$\boldsymbol{R_f}$ = 0.45 (SiO_2, pentane/EtOAc 80:20).
^{1}H NMR (600 MHz, $CDCl_3$, 25 °C): δ = 7.84 – 7.77 (m, 3H, $H_c/H_f/H_g$), 7.66 (s, 1H, H_m), 7.50 – 7.41 (m, 2H, H_a/H_b), 7.37 (dd, *J* = 8.5, 1.8 Hz, 1H, H_h), 3.45 (sextet, *J* = 7.1 Hz, 1H, H_n), 2.78 (dd, *J* = 15.6, 6.9 Hz, 1H, H_p), 2.67 (dd, *J* = 15.6, 8.1 Hz, 1H, H_p), 1.40 (d, *J* = 6.9 Hz, 3H, H_o) ppm. The labile acid proton was not observed.
^{13}C{^{1}H} NMR (151 MHz, $CDCl_3$, 25 °C): δ = 178.3 (C^q), 143.0 (C^k), 133.7 (C^e), 132.5 (C^d), 128.4 (C^g), 127.8 (C^a/C^b), 127.7 (C^a/C^b), 126.2 (C^h), 125.6 (C^c/C^f), 125.5 (C^c/C^f), 125.1 (C^m), 42.6 (C^p), 36.4 (C^n), 22.0 (C^o) ppm.
HRMS (ESI-): calculated for $[M - H]^-$ 213.09210, found 213.09226.
$\boldsymbol{[\alpha]_D^{20}}$: –37 (c 0.065, CH_2Cl_2).
The NMR data match those reported in the literature for *S*-**4**.[2]
S-**4** was obtained in a similar yield starting from *S*-**1**.
S-**4**: $\boldsymbol{[\alpha]_D^{20}}$: +34 (c 0.097, CH_2Cl_2).

(*R*)-3-methyl-2,3-dihydro-1H-cyclopenta[a]naphthalen-1-one (*R*-5)

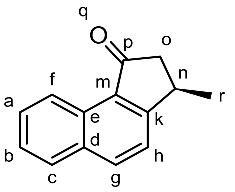

Trifluoromethanesulfonic acid (20 mL) was added to a solution of *R*-**4** (4.18 g, 19.5 mmol, 1.0 eq.) in CH_2Cl_2 (25 mL) at 0 °C. The reaction mixture was stirred for 45 min at 0 °C and quenched by the addition of ice/water. EtOAc was added (120 mL) and the aqueous phase was extracted with EtOAc (120 mL). The combined organic layers were washed with a saturated aqueous solution of $NaHCO_3$ (2 x 150 mL), brine (150 mL), dried over Na_2SO_4 and the solvent was removed *in vacuo*. The crude product was purified by column chromatography (SiO_2, pentane/EtOAc, 90:10) to afford *R*-**5** as a colorless solid (2.61 g, 13.3 mmol, 68%).

***R*$_f$** = 0.30 (SiO_2, pentane/CH_2Cl_2 70:30).
^{1}H NMR (600 MHz, $CDCl_3$, 25 °C): δ = 9.16 (d, *J* = 8.3 Hz, 1H, H_f), 8.06 (d, *J* = 8.4 Hz, 1H, H_g), 7.89 (d, *J* = 8.1 Hz, 1H, H_c), 7.67 (ddd, *J* = 8.3, 6.9, 1.3 Hz, 1H, H_a), 7.56 (m, 2H, H_b/H_h), 3.51 (td, *J* = 7.2, 2.9 Hz, 1H, H_n), 3.06 (dd, *J* = 18.7, 7.1 Hz, 1H, H_o), 2.41 (dd, *J* = 18.7, 3.0 Hz, 1H, H_o), 1.46 (d, *J* = 7.2 Hz, 3H, H_r) ppm.
^{13}C{^{1}H} NMR (151 MHz, $CDCl_3$, 25 °C): δ = 207.0 (C^p), 163.0 (C^k), 136.0 (C^g), 132.7 (C^d), 130.4 (C^e), 129.4 (C^m), 129.1 (C^a), 128.2 (C^c), 126.7 (C^h), 124.3 (C^f), 122.7 (C^b), 46.1 (C^o), 32.9 (C^n), 21.4 (C^r) ppm.
HRMS (ESI+): calculated for $[M + H]^+$ 197.09609, found 197.09597.
HPLC (Chiralcel OJ-H, *n*-heptane/2-propanol 95:5, 1.0 mL/min): retention times (min) 6.69 (*R*-**5**, major), 7.63 (*S*-**5**, minor), *ee* = 81%.
$[\alpha]_D^{20}$: +21 (c 0.260, CH_2Cl_2)

The NMR data match those reported in the literature for *S*-**5**.[2]
S-**5** was obtained in a similar yield starting from *S*-**4**.
As the initial enantiopure precursor was changed compared to the reported procedure, *ee* was also determined for *S*-**5**.
HPLC (Chiralcel OJ-H, *n*-heptane/^{i}PrOH 95:5, 1.0 mL/min): retention times (min) 6.39 (*R*-**5**, minor), 7.03 (*S*-**5**, major), *ee* = 84%.
$[\alpha]_D^{20}$: –25 (c 0.395, CH_2Cl_2)

(2*R*,3*R*)-2,3-dimethyl-2,3-dihydro-1H-cyclopenta[*a*]naphthalen-1-one (*R*-6)

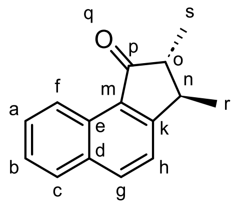

n-BuLi (1.6 M in hexanes, 1.60 mL, 2.62 mmol, 1.0 eq.) was added dropwise to a solution of diisopropylamine (0.37 mL, 2.62 mmol, 1.0 eq.) in anhydrous THF (8 mL) at 0 °C. After stirring at this temperature for 30 min the mixture was cooled to –78 °C and *R*-**5** (500 mg, 2.55 mmol, 1.0 eq.) in anhydrous THF (8 mL) was added dropwise. The reaction mixture was stirred for 30 min at –78 °C before HMPA (0.45 mL, 2.55 mmol, 1.0 eq.) and MeI (0.56 mL, 8.92 mmol, 3.5 eq.) were added. The cooling bath was then removed and the reaction mixture was stirred for 16 h at room temperature, before quenching with a saturated aqueous solution of NH_4Cl. The aqueous phase was extracted with EtOAc (3 x 20 mL) and the combined organic layers were washed with brine (3 x 20 mL), dried over Na_2SO_4 and concentrated *in vacuo*. The crude product was purified by column chromatography (SiO_2, pentane/EtOAc, 95:5) to afford *R*-**6** as a yellow solid (407 mg, 1.94 mmol, 76%, de > 99% by NMR).

R_f = 0.41 (SiO_2, pentane/CH_2Cl_2 70:30).
^{1}H NMR (600 MHz, $CDCl_3$, 25 °C): δ = 9.15 (d, *J* = 8.4 Hz, 1H, H_f), 8.07 (d, *J* = 8.4 Hz, 1H, H_g), 7.89 (d, *J* = 8.2 Hz, 1H, H_c), 7.67 (ddd, *J* = 8.3, 6.9, 1.3 Hz, 1H, H_a), 7.58 – 7.52 (m, 2H, H_b/H_k), 3.03 (qd, *J* = 7.1, 3.7 Hz, 1H, H_n), 2.35 (qd, *J* = 7.4, 3.8 Hz, 1H, H_o), 1.50 (d, *J* = 7.2 Hz, 3H, H_r), 1.38 (d, *J* = 7.4 Hz, 3H, H_s) ppm.
^{13}C{^{1}H} NMR (151 MHz, $CDCl_3$, 25 °C): δ = 209.1 (C^p), 160.7 (C^k), 136.0 (C^g), 132.8 (C^d), 129.7 (C^e), 129.4 (C^m), 129.1 (C^a), 128.2 (C^c), 126.7 (C^h), 124.4 (C^f), 122.4 (C^b), 51.9 (C^o), 41.9 (C^n), 19.5 (C^r), 14.8 (C^s) ppm.
HRMS (ACPI+): calculated for $[M + H]^+$ 211.11174, found 211.11094.
HPLC (Chiralcel OD-H, *n*-heptane/2-propanol 99.4:0.6, 0.6 mL/min): retention times (min) 18.41 (*R*-**6**, major), 21.06 (*S*-**6**, minor), *ee* = 84%.
$[\alpha]_D^{20}$: –25 (c 0.111, CH_2Cl_2).

The NMR data match those reported in the literature for *S*-**6**.[2]
S-**6** was obtained in a similar yield starting from *S*-**5**.
As the initial enantiopure precursor was changed compared to the reported procedure, *ee* was also determined for *S*-**6**.
HPLC (Chiralcel OD-H, *n*-heptane/2-propanol 99.4:0.6, 0.6 mL/min): retention times (min) 18.29 (*R*-**6**, minor), 19.50 (*S*-**6**, major), *ee* = 87%.
$[\alpha]_D^{20}$: +20 (c 0.098, CH_2Cl_2).

(2*S*,2'*S*,3*S*,3'*S*)-2,2',3,3'-tetramethyl-2,2',3,3'-tetrahydro-1,1'-bi(cyclopenta[a]naphthalenylidene) (*S*-M1)

Zn powder (404 mg, 6.18 mmol, 6.5 eq.) was stirred using a magnetic stirring bar under vacuum in a Schlenk flask for 30 min to scratch the passivation layer. Then, under an N_2 atmosphere, anhydrous THF (2 mL) was added, and the reaction mixture was cooled down to 0 °C. Titanium(IV)chloride (0.21 mL, 1.90 mmol, 2.0 eq.) was added dropwise. The reaction mixture was heated at reflux for 3 h, cooled down to room temperature and a solution of *S*-**6** (200 mg, 0.95 mmol, 1.0 eq.) in anhydrous THF (1 mL) was added. The flask was placed in an oil bath preheated at 68 °C and the mixture was stirred for 72 h. After cooling to room temperature, the reaction mixture was filtered through celite, concentrated *in vacuo,* and quenched with 1 M aqueous HCl (20 mL). The aqueous phase was extracted with Et_2O (4 x 20 mL). The combined organic layers were washed with brine (30 mL), dried over $MgSO_4$, and concentrated *in vacuo*. The crude product was purified by column chromatography (SiO_2, pentane/CH_2Cl_2, 95:5) to afford a mixture of *S*-*E*-**M1** and *S*-*Z*-**M1** as a yellow solid (86 mg, 0.22 mmol, 46%). Pure *S*-*E*-**M1**, consisting of stable *S*-E_s-**M1** and metastable *S*-E_{ms}-**M1** in a 2:1 ratio was obtained by selective precipitation from a CH_2Cl_2/MeOH solution of the *E*/*Z* mixture induced by rotary evaporation of the CH_2Cl_2.

***R*$_f$** = 0.23 (SiO_2, pentane).
HRMS (ESI+): calculated for $[M]^+$ 388.21855, found 388.21783.

S-E_s-**M1**:

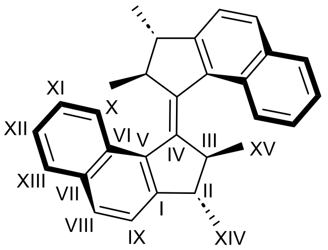

^{1}H NMR (600 MHz, $CDCl_3$, 25 °C): δ = 8.25 (d, *J* = 8.4 Hz, 2H, H_X), 7.91 – 7.88 (m, 2H, H_{XIII}), 7.76 (d, *J* = 8.1 Hz, 2H, H_{VIII}), 7.53 (ddd, *J* = 8.3, 6.7, 1.4 Hz, 2H, H_{XI}), 7.49 – 7.41 (m, 2H, H_{XII}), 7.37 (d, *J* = 8.1 Hz, 2H, H_{IX}), 2.69 (q, *J* = 6.5 Hz, 2H, H_{II}), 2.66 (q, *J* = 7.1 Hz, 2H, H_{III}), 1.37 (d, *J* = 6.5 Hz, 6H, H_{XIV}), 0.95 (d, *J* = 7.0 Hz, 6H, H_{XV}) ppm.
^{13}C{^{1}H} NMR (151 MHz, $CDCl_3$, 25 °C): δ = 147.1 (C^{I}), 142.0 (C^{IV}), 137.6 (C^{V}), 133.3 (C^{VII}), 130.5 (C^{VI}), 128.6 (C^{XIII}), 128.3 (C^{VIII}), 127.1 (C^{X}), 125.0 (C^{XI}), 124.8 (C^{XII}), 123.6 (C^{IX}), 49.8 (C^{II}), 47.9 (C^{III}), 20.3 (C^{XIV}), 20.0 (C^{XV}) ppm.
HPLC (Chiralcel OD-H, *n*-heptane, 0.5 mL/min): retention times (min) 20.93 (*S*-E_{ms}-**M1**, minor), 23.01 (*S*-E_s-**M1**, major), 25.51 (*S*-*Z*-**M1**, minor), *ee* >99%.
$[\alpha]_D^{20}$: –606 (c 0.035, CH_2Cl_2).

S-E_{ms}-**M1**

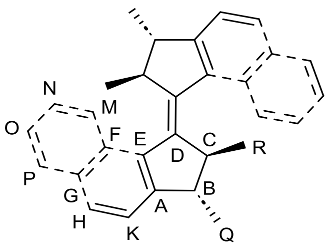

^{1}H NMR (600 MHz, $CDCl_3$, 25 °C): δ = 7.95 (d, *J* = 8.3 Hz, 2H, H_J), 7.91 – 7.88 (m, 2H, H_M), 7.82 (d, *J* = 8.2 Hz, 2H, H_H), 7.49 – 7.41 (m, 6H, $H_K/H_N/H_O$), 3.12 – 3.08 (m, 2H, H_B), 3.08 – 3.02 (m, 2H, H_C), 1.44 (d, *J* = 6.6 Hz, 6H, H_Q), 0.64 (d, *J* = 5.8 Hz, 6H, H_R) ppm.
^{13}C{^{1}H} NMR (151 MHz, $CDCl_3$, 25 °C): δ = 149.5 (C^A), 142.0 (C^D), 139.7 (C^E), 132.7 (C^G), 129.8 (C^F), 128.8 (C^K or C^N or C^O), 128.6 (C^H), 126.5 (C^K or C^N or C^O), 125.1 (C^M), 124.9 (C^P), 121.2 (C^K or C^N or C^O), 56.7 (C^C), 48.9 (C^B), 17.9 (C^Q), 17.3 (C^R) ppm.

S-*Z*-**M1**:

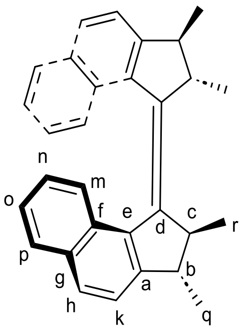

^{1}H NMR (600 MHz, $CDCl_3$, 25 °C): δ = 7.72 (d, *J* = 8.1 Hz, 2H, H_h), 7.65 (dd, *J* = 8.3, 1.3 Hz, 2H , H_p), 7.49 – 7.41 (m, 2H, H_k) 6.96 (ddd, *J* = 8.1, 6.7, 1.2 Hz, 2H, H_o), 6.60 (d, *J* = 8.5, 2H, H_m), 6.37 (ddd, *J* = 8.3, 6.7, 1.3 Hz, 2H, H_n), 3.13 (q, *J* = 6.8 Hz, 2H, H_b), 2.96 (q, *J* = 7.0 Hz, 2H, H_c), 1.50 (d, *J* = 7.1 Hz, 6H, H_q), 1.23 (d, *J* = 6.9 Hz, 6H, H_r) ppm.
^{13}C{^{1}H} NMR (151 MHz, $CDCl_3$, 25 °C): δ = 149.2 (C_a), 139.7 (C_d), 136.0 (C_e), 132.6 (C_g), 130.1 (C_f), 128.8 (C_h), 127.8 (C_p), 126.9 (C_m), 124.3 (C_n), 124.3 (C_o), 123.0 (C_k), 50.6 (C_b), 47.1 (C_c), 20.7 (C_q), 20.2 (C_r) ppm.

(2*R*,2'*R*,3*R*,3'R)-2,2',3,3'-tetramethyl-2,2',3,3'-tetrahydro-1,1'-bi(cyclopenta[a]naphthalenylidene) (*R*-M1)

Zn powder (404 mg, 6.18 mmol, 6.5 eq.) was stirred using a magnetic stirring bar under vacuum in a Schlenk flask for 30 min to scratch the passivation layer. Then, under an N_2 atmosphere, anhydrous THF (2 mL) was added, and the reaction mixture was cooled down to 0 °C. Titanium(IV)chloride (0.21 mL, 1.90 mmol, 2.0 eq.) was added dropwise. The reaction mixture was heated at reflux for 3 h, cooled down to room temperature and a solution of *R*-**6** (200 mg, 0.95 mmol, 1.0 eq.) in anhydrous THF (1 mL) was added. The flask was placed an oil bath preheated at 68 °C and the mixture was stirred for 72 h. After cooling to room temperature, the reaction mixture was filtered through celite, concentrated *in vacuo* and quenched with 1 M aqueous hydrochloric acid (20 mL). The aqueous phase was extracted with Et_2O (4 x 20 mL). The combined organic layers were washed with brine (30 mL), dried over $MgSO_4$ and concentrated *in vacuo*. The crude product was purified by column chromatography (SiO_2, pentane/CH_2Cl_2, 95:5) to afford a mixture of *R*-*E*-**M1** and *R*-*Z*-**M1** as a yellow solid (89 mg, 0.23 mmol, 48%). Pure *R*-*E*-**M1**, consisting of *R*-E_s-**M1** and *R*-E_{ms}-**M1** in a 2:1 ratio was obtained by selective precipitation from a CH_2Cl_2/MeOH solution of the *E*/*Z* mixture induced by rotary evaporation of the CH_2Cl_2.

$\boldsymbol{R_f}$ = 0.23 (SiO_2, pentane).
^{1}H and ^{13}C NMR are identical to *S*-*E*-**M1**.
HRMS (ESI+): calculated for $[M]^+$ 388.21855, found 388.21790.
HPLC (Chiralcel OD-H, *n*-heptane, 0.5 mL/min): retention times (min) 27.01 (*R*-Z-**M1**, minor), 90.94 (*R*-E_s-**M1**, major), *ee* >99%.
$[\alpha]_D^{20}$: +608 (c 0.009, CH_2Cl_2).

(3*S*,3'*S*)-3,3'-dimethyl-2,2',3,3'-tetrahydro-1,1'-bi(cyclopenta[a]naphthalenylidene) (*S*-M2)

Zn powder (433 mg, 6.62 mmol, 6.5 eq.) was stirred using a magnetic stirring bar under vacuum in a Schlenk flask for 30 min to scratch the passivation layer. Then, under an N_2 atmosphere, anhydrous THF (2 mL) was added, and the reaction mixture was cooled down to 0 °C. Titanium(IV)chloride (0.22 mL, 2.04 mmol, 2.0 eq.) was added dropwise. The reaction mixture was heated at reflux for 3 h, cooled to room temperature and a solution of *S*-**5** (200 mg, 1.02 mmol, 1.0 eq.) in anhydrous THF (1 mL) was added. The flask was placed in an oil bath preheated at 68 °C and the mixture was stirred for 72 h. After cooling to room temperature, the reaction mixture was filtered through celite, concentrated *in vacuo* and quenched with 1 M aqueous hydrochloric acid (20 mL). The aqueous phase was extracted with Et_2O (4 x 20 mL). The combined organic layers were washed with brine (30 mL), dried over $MgSO_4$ and concentrated *in vacuo*. The crude product was purified by column chromatography (SiO_2, pentane/CH_2Cl_2, 95:5) to afford a mixture of *S*-*E*-**M2** and *S*-*Z*-**M2** as a yellow solid (133 mg, 0.369 mmol, 72%). Pure *S*-*E*-**M2** was obtained by selective precipitation from a CH_2Cl_2/MeOH solution of the *E*/*Z* mixture induced by rotary evaporation of the CH_2Cl_2.

$\boldsymbol{R_f}$ = 0.26 (SiO_2, pentane).
HRMS (ACPI+): calculated for $[M + H]^+$ 361.19508, found 361.19418.

S-*E*-**M2**:

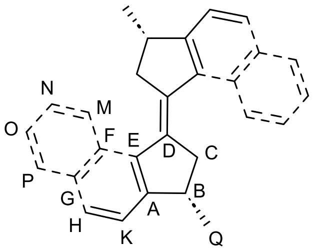

^{1}H NMR (600 MHz, $CDCl_3$, 25 °C): δ = 8.04 (d, *J* = 8.4 Hz, 2H, H_M), 7.91 (d, *J* = 8.2 Hz, 2H, H_P), 7.84 (d, *J* = 8.2 Hz, 2H, H_H), 7.54 (apparent t, *J* = 7.5 Hz, 2H, H_N), 7.51 – 7.39 (m, 4H, H_K/H_O), 3.23 (br. s, 2H, H_B), 2.89 – 2.66 (m, 4H, H_C), 1.35 (br. s, 6H, H_Q) ppm.
^{13}C{^{1}H} NMR (151 MHz, $CDCl_3$, 25 °C): δ = 149.0 (C^A), 139.4 (C^E), 135.2 (C^D), 133.3 (C^G), 128.8 (C^H), 128.7 (C^P), 127.0 (C^M), 125.6 (C^N), 124.9 (C^O), 121.6 (C^K), 48.5 (C^C), 39.3 (C^B), 19.5 (C^Q) ppm.
HPLC (Chiralcel OD-H, *n*-heptane/2-propanol 99.5:0.5, 0.5 mL/min): retention times (min) 13.41 (*R*-*E*-**M2**, minor), 14.67 (*S*-*E*-**M2**, major), *ee* = 98%.
$[\alpha]_D^{20}$: +38 (c 0.123, CH_2Cl_2).

The ^{13}C NMR signal for C^F is overlapping with the signals of C^H and C^P as assigned by analysis of the ^{1}H–^{13}C–HMBC NMR spectrum.

S-*Z*-**M2**:

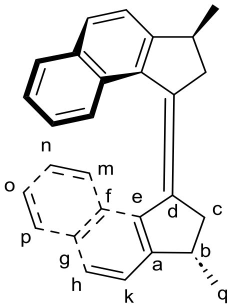

^{1}H NMR (600 MHz, $CDCl_3$, 25 °C): δ = 7.74 (d, *J* = 8.2 Hz, 2H, H_h), 7.66 (d, *J* = 8.1 Hz, 2H, H_p), 7.51 – 7.43 (m, 2H, H_k), 7.01 – 6.95 (m, 2H, H_o), 6.71 (d, *J* = 8.5 Hz, 2H, H_m), 6.37 – 6.44 (m, 2H, H_n), 3.64 (q, *J* = 7.1 Hz, 2H, H_b), 3.37 (dd, *J* = 12.7, 6.3 Hz, 2H, $H_{c''}$), 2.84 – 2.77 (m, 2H, $H_{c'}$), 1.49 (d, *J* = 6.7 Hz, 6H, H_q) ppm.
^{13}C{^{1}H} NMR (151 MHz, $CDCl_3$, 25 °C): δ = 150.3 (C^a), 138.4 (C^d), 134.1 (C^e), 132.4 (C^h), 129.1 (C^k), 128.6 (C^g), 127.8 (C^m), 126.9 (C^p), 124.5 (C^o), 124.4 (C^n), 121.3 (C^f), 48.0 (C^a), 38.5 (C^b), 19.8 (C^q) ppm.

(3*R*,3'*R*)-3,3'-dimethyl-2,2',3,3'-tetrahydro-1,1'-bi(cyclopenta[a]naphthalenylidene) (*R*-M2)

Zn powder (433 mg, 6.62 mmol, 6.5 eq.) was stirred using a magnetic stirring bar under vacuum in a Schlenk flask for 30 min to scratch the passivation layer. Then, under an N_2 atmosphere, anhydrous THF (2 mL) was added, and the reaction mixture was cooled down to 0 °C. Titanium(IV)chloride (0.22 mL, 2.04 mmol, 2.0 eq.) was added dropwise. The reaction mixture was heated at reflux for 3 h, cooled down to room temperature and a solution of *R*-**5** (200 mg, 1.02 mmol, 1.0 eq.) in anhydrous THF (1 mL) was added. The flask was placed in an oil bath preheated at 68 °C and the mixture was stirred for 72 h. After cooling to room temperature, the reaction mixture was filtered through celite, concentrated *in vacuo* and quenched with 1 M aqueous hydrochloric acid (20 mL). The aqueous phase was extracted with Et_2O (4 x 20 mL). The combined organic layers were washed with brine (30 mL), dried over $MgSO_4$ and concentrated *in vacuo*. The crude product was purified by column chromatography (SiO_2, pentane/CH_2Cl_2, 95:5) to afford a mixture of *R*-*E*-**M2** and *R*-*Z*-**M2** as a yellow solid (140 mg, 0.39 mmol, 76%). Pure *R*-*E*-**M2** was obtained by selective precipitation from a CH_2Cl_2/MeOH solution of the *E*/*Z* mixture induced by rotary evaporation of the CH_2Cl_2.

$\boldsymbol{R_f}$ = 0.26 (SiO_2, pentane).
^{1}H and ^{13}C NMR are identical to *S*-*E*-**M2**.
HRMS (ESI+): calculated for $[M]^{+\cdot}$ 360.18725, found 360.18744.
HPLC (Chiralcel OD-H, *n*-heptane/2-propanol 99.5:0.5, 0.5 mL/min): retention times (min) 13.27 (*R*-*E*-**M2**, major), 14.88 (*S*-*E*-**M2**, minor), *ee* 94%.
$[\alpha]_D^{20}$: –41 (c 0.094, CH_2Cl_2).

2. In-situ NMR irradiation and relaxation experiments

2.1. ^{1}H NMR study of M1

The following experiments were all performed with the same enantiomer of **M1**. For clarity, the stereodescriptors corresponding to point chirality and helicity will be omitted in the legends and the following notation will be used:

E_s-**M1**	(2*S*,2'*S*,3*S*,3'*S*)-(*M*,*M*)-*E*-**M1**
E_{ms}-**M1**	(2*S*,2'*S*,3*S*,3'*S*)-(*P*,*P*)-*E*-**M1**
Z_s-**M1**	(2*S*,2'*S*,3*S*,3'*S*)-(*M*,*M*)-*Z*-**M1**
Z_{ms}-**M1**	(2*S*,2'*S*,3*S*,3'*S*)-(*P*,*P*)-*Z*-**M1**

The exact same behavior is expected from the opposite enantiomer, with an inversion of the helicity and point chirality stereodescriptors.

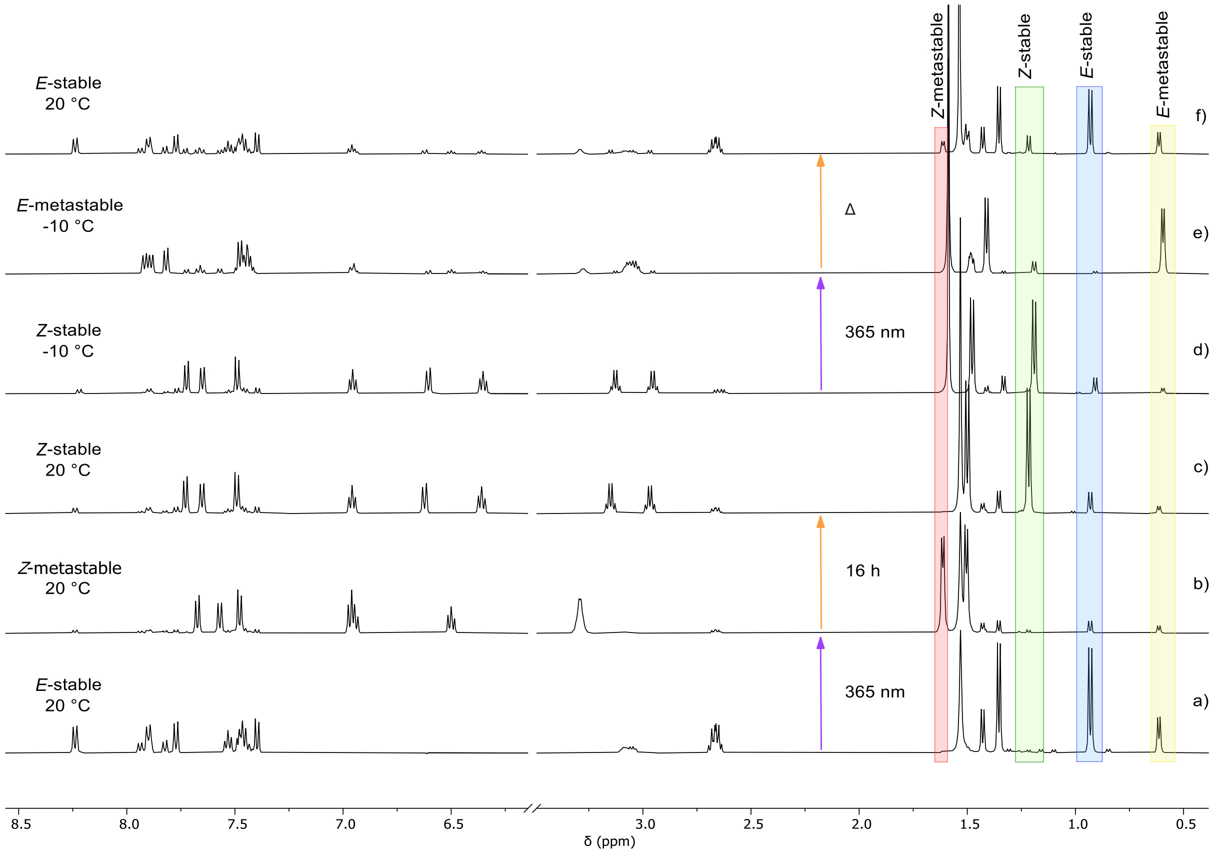

Figure S1. Full rotational cycle of **M1** monitored by variable temperature ^{1}H NMR spectroscopy (500 MHz, CH_2Cl_2). a) Initial sample of E_s-**M1** at 20 °C, b) PSS reached after in-situ irradiation with 365 nm showing a majority of Z_{ms}-**M1**, c) Mixture obtained after complete relaxation at 20 °C over 16 h composed of a majority of Z_s-**M1**, d) Same mixture at –10 °C, e) PSS reached after in-situ irradiation with 365 nm showing a majority of E_{ms}-**M1**, d) Mixture obtained at 20 °C after complete relaxation composed of a majority of E_s-**M1** after a complete four-step unidirectional rotation cycle.

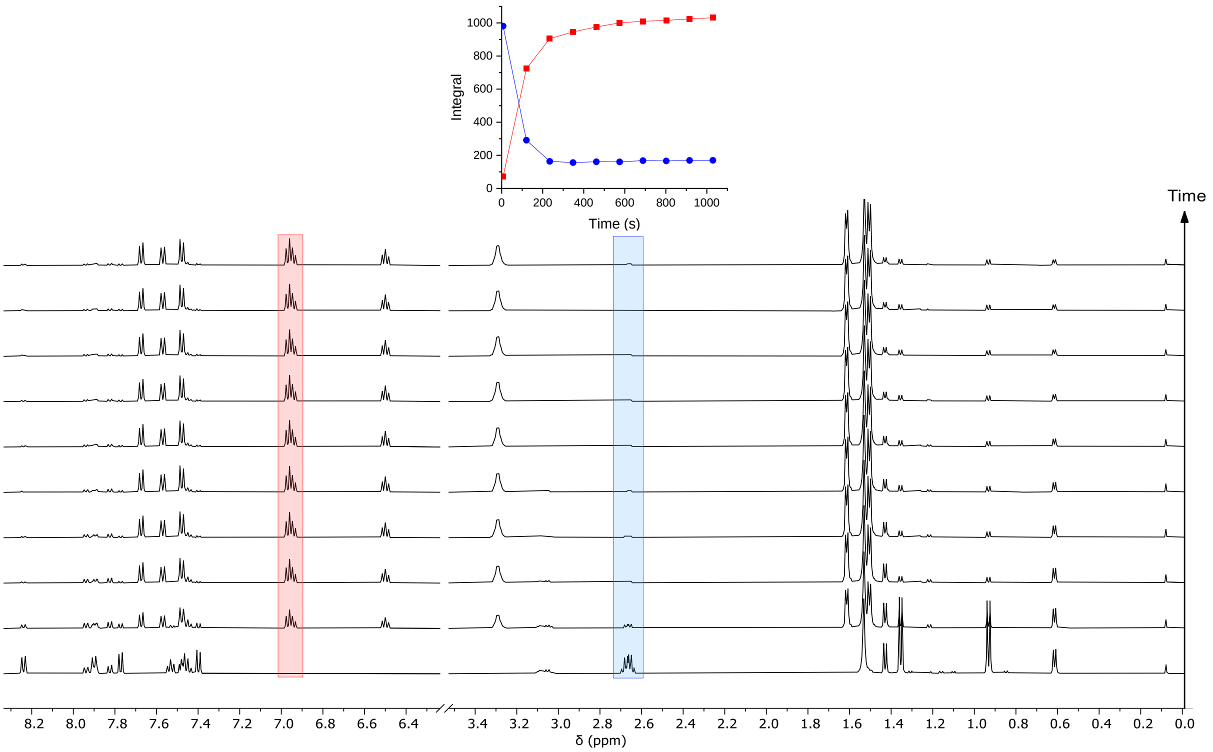

Figure S2. ^{1}H NMR (500 MHz, CH_2Cl_2, 20 °C) monitoring of the conversion of E_s-**M1** to Z_{ms}-**M1** upon in-situ irradiation with 365 nm UV light.

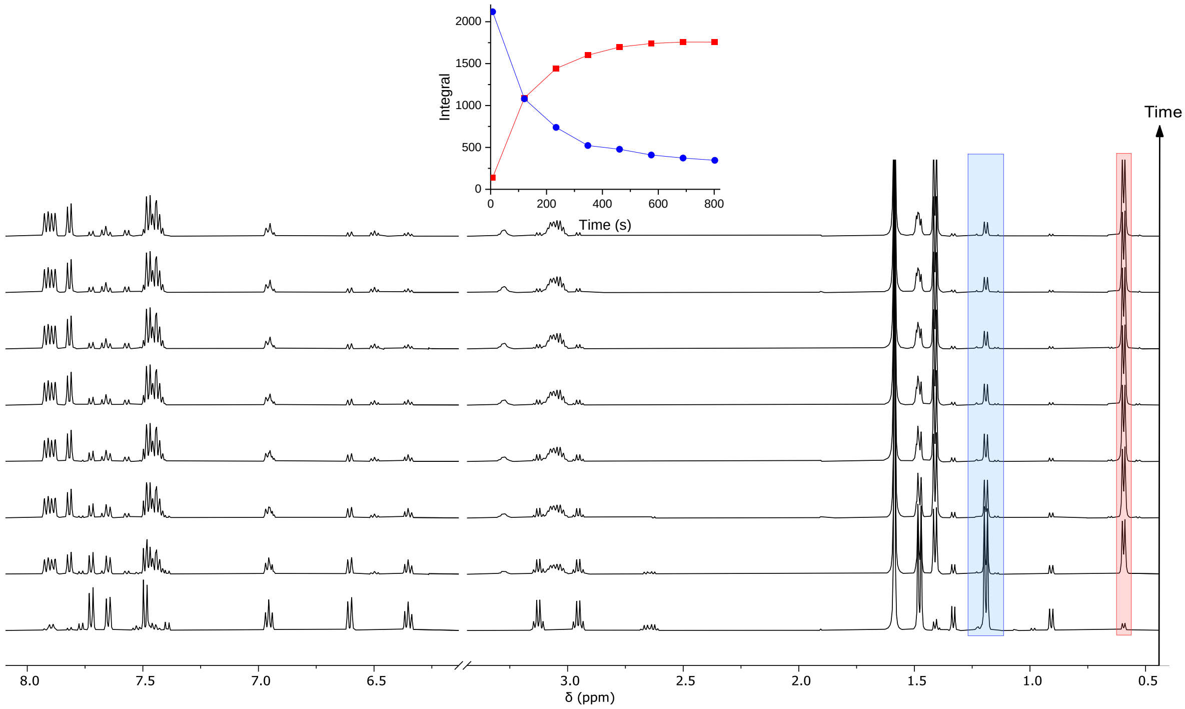

Figure S3. ^{1}H NMR (500 MHz, CH_2Cl_2, –10 °C) monitoring of the conversion of Z_s-**M1** to E_{ms}-**M1** upon in-situ irradiation with 365 nm UV light.

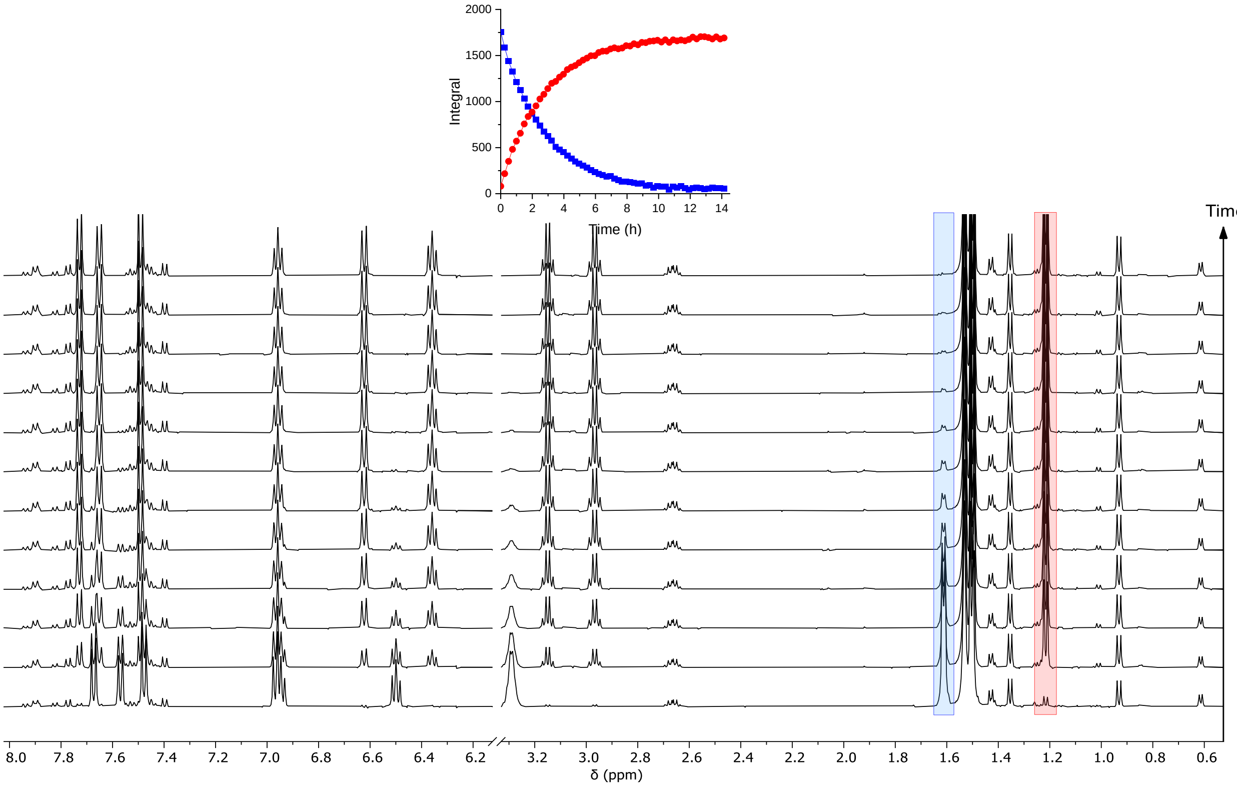

Figure S4. ^{1}H NMR (500 MHz, CH_2Cl_2, 20 °C) monitoring of the thermal helix inversion of Z_{ms}-**M1** to Z_s-**M1** at 20 °C.

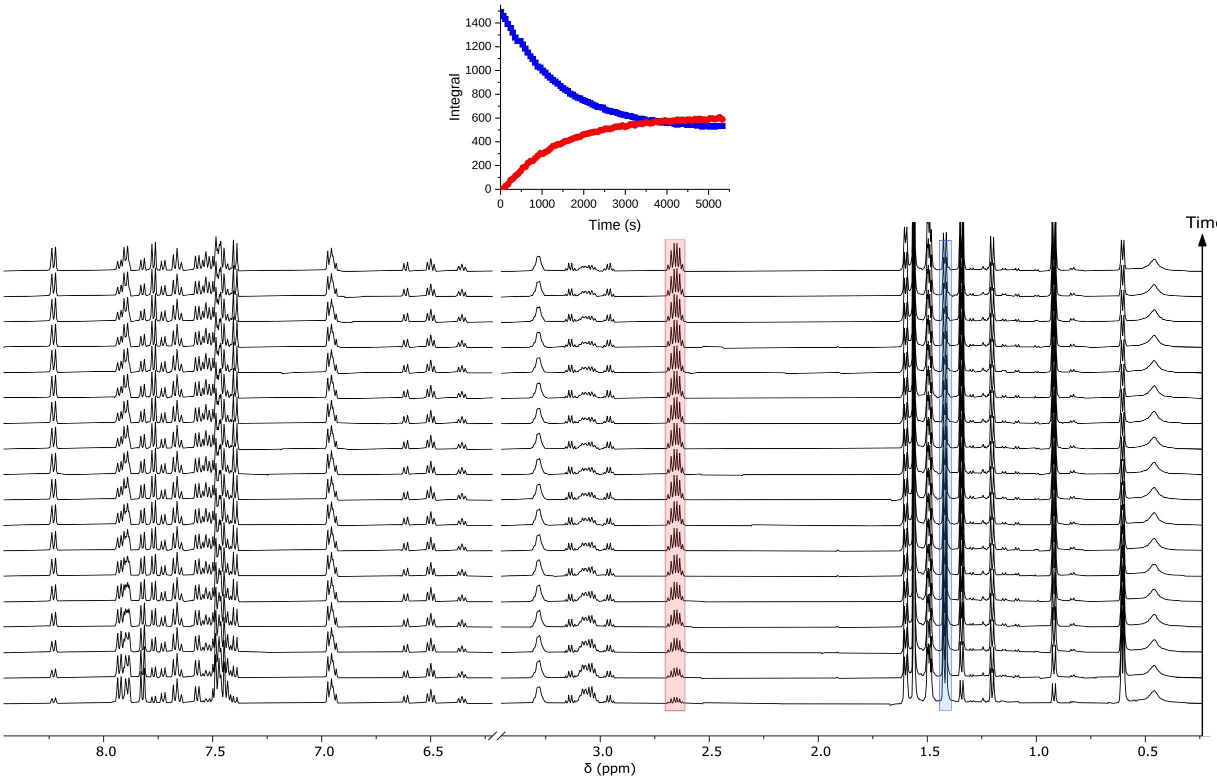

Figure S5. ^{1}H NMR (500 MHz, CH_2Cl_2, 5 °C) monitoring of the thermal helix inversion of E_{ms}-**M1** to stable E_s-**M1** at 5 °C.

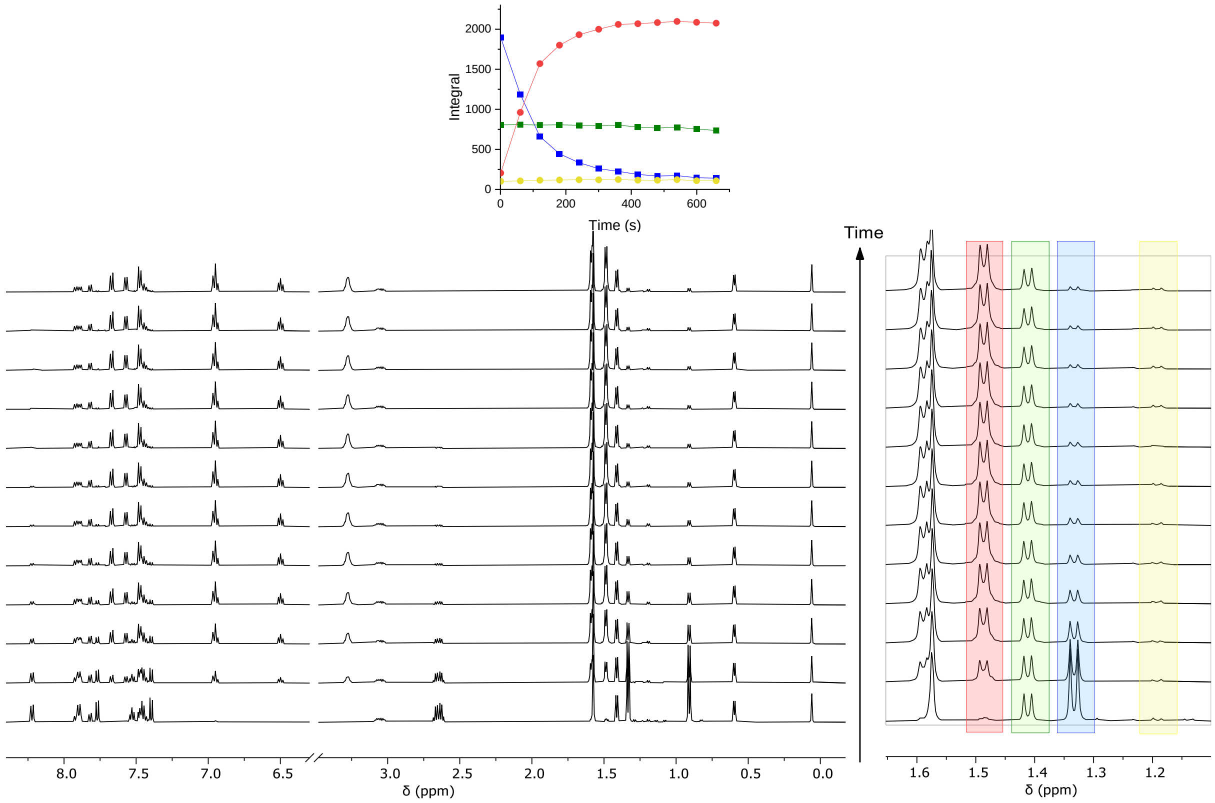

Figure S6. ^{1}H NMR (500 MHz, CH_2Cl_2, –30 °C) evidencing the fully unidirectional rotation mechanism of **M1** despite an initial 72:28 ratio of E_s-**M1** and E_{ms}-**M1**. In situ irradiation with 365 nm UV light resulted in the conversion of E_s-**M1** (blue) into Z_{ms}-**M1** (red) whereas metastable E_{ms}-**M1** (green) was not converted into Z_s-**M1** (yellow) by rotation in the opposite direction.

2.2. ^{1}H NMR study of M2

The following experiments were all performed with the same enantiomer of **M2**. For clarity, the stereodescriptors corresponding to point chirality and helicity will be omitted in the legends and the following notation will be used:

E_s-**M2**	(3*S*,3'*S*)-(*P*,*P*)-*E*-**M2**
E_{ms}-**M2**	(3*S*,3'*S*)-(*M*,*M*)-*E*-**M2**
Z_s-**M2**	(3*S*,3'*S*)-(*P*,*P*)-*Z*-**M2**
Z_{ms}-**M2**	(3*S*,3'*S*)-(*M*,*M*)-*Z*-**M2**

The exact same behavior is expected from the opposite enantiomer, with an inversion of the helicity and point chirality stereodescriptors.

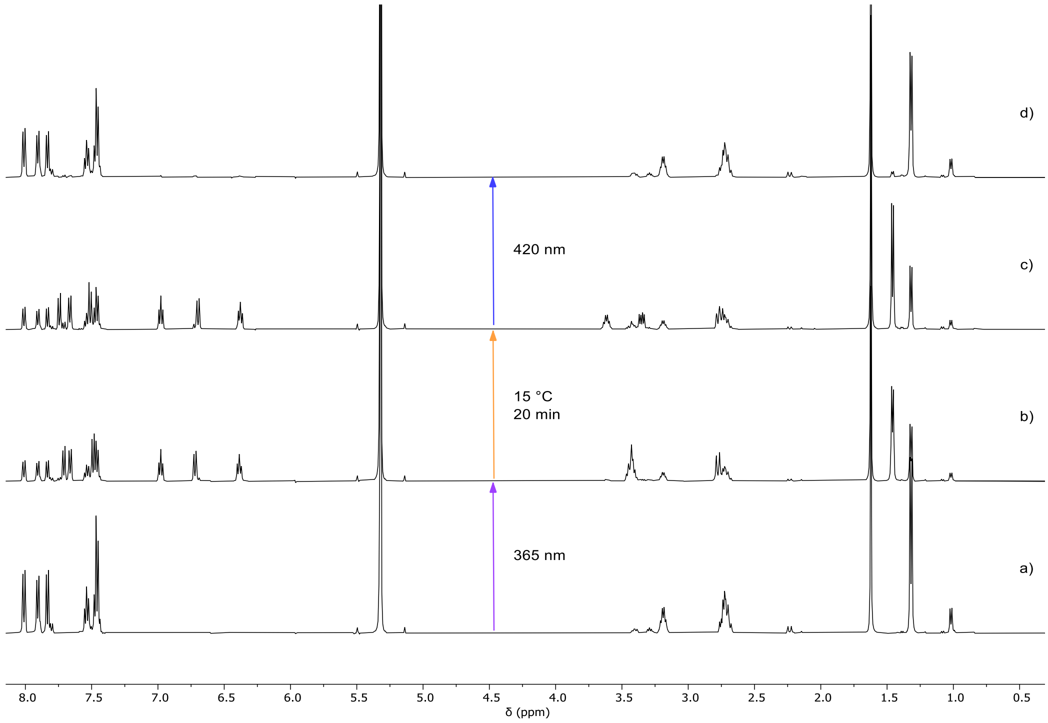

Figure S7. Full rotational cycle of **M2** monitored by ^{1}H NMR spectroscopy (500 MHz, CH_2Cl_2, –30 °C). a) Initial sample of pure E_s-**M2**, b) PSS reached after in-situ irradiation with 365 nm showing a majority of metastable Z_{ms}-**M2**, c) Mixture obtained after complete relaxation at 15 °C over 20 min composed of a majority of Z_s-**M2**, d) E_s-**M2** recovered after a complete rotation cycle.

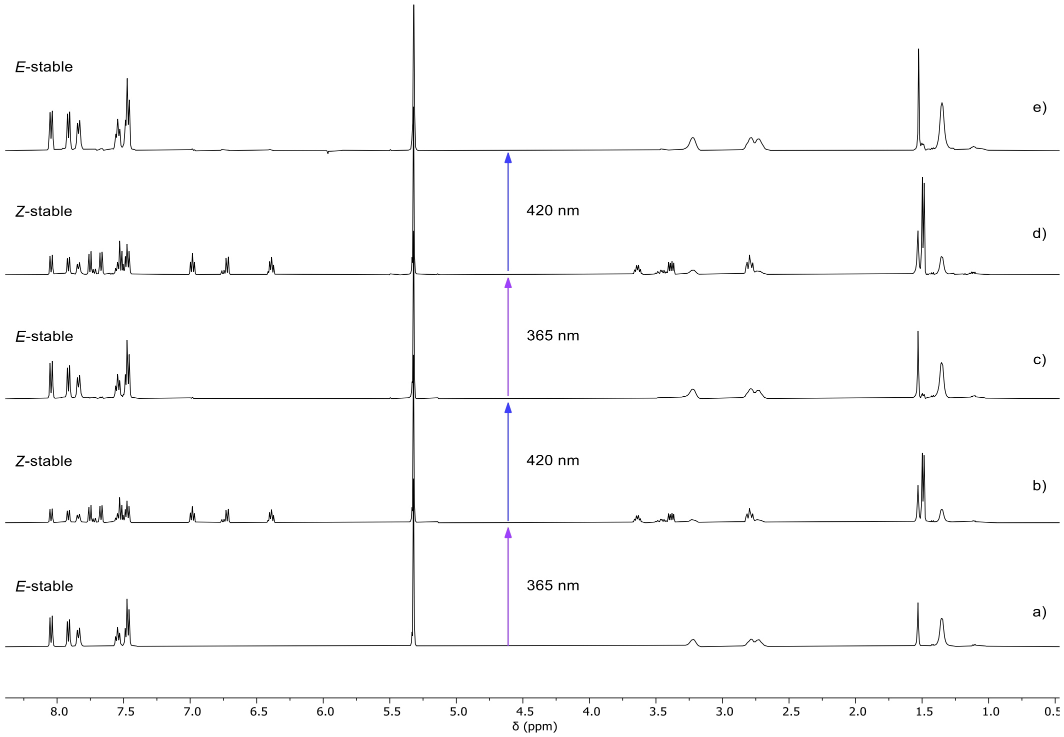

Figure S8. Switch-like behavior of **M2** monitored by ^{1}H NMR spectroscopy (500 MHz, CH_2Cl_2, 20 °C). a) Initial sample of pure E_s-**M2**, b) Mixture obtained ca. 5 min after in situ irradiation with 365 nm UV light composed of a 56:46 ratio of Z_s-**M2** and E_s-**M2**, c) Pure E_s-**M2** regenerated after in situ irradiation with 420 nm light, d), e) Repetition of the process.

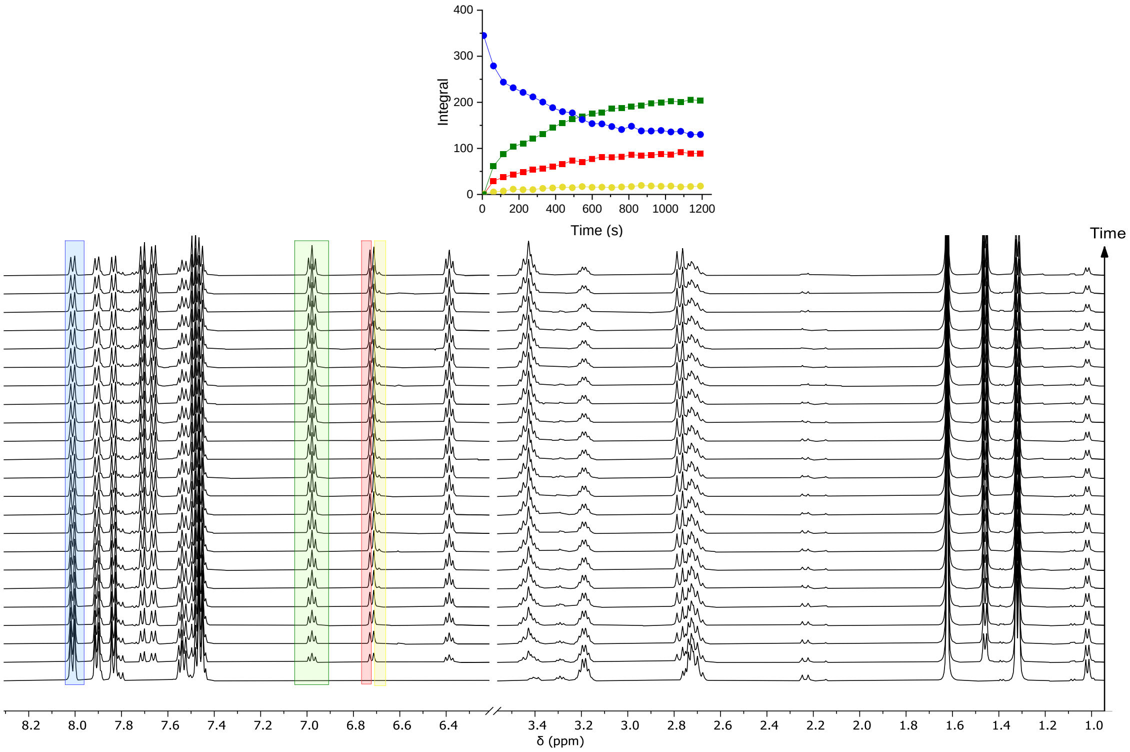

Figure S9. ^{1}H NMR (500 MHz, CH_2Cl_2, –30 °C) monitoring of the conversion of stable *E*s-**M2** (blue) to *Z*ms-**M2** upon in-situ irradiation with 365 nm UV light at –30 °C. Most of the signals of *Z*ms-**M2** are similar to the ones of its stable counterpart *Z*s-**M2** (ex. triplet highlighted in green) but two doublets were observed at 6.72 ppm (red, *Z*ms-**M2**) and 6.70 ppm (yellow, *Z*s-**M2**) as well as distinct CH_2 signals around 2.7 ppm, allowing for the monitoring of the process.

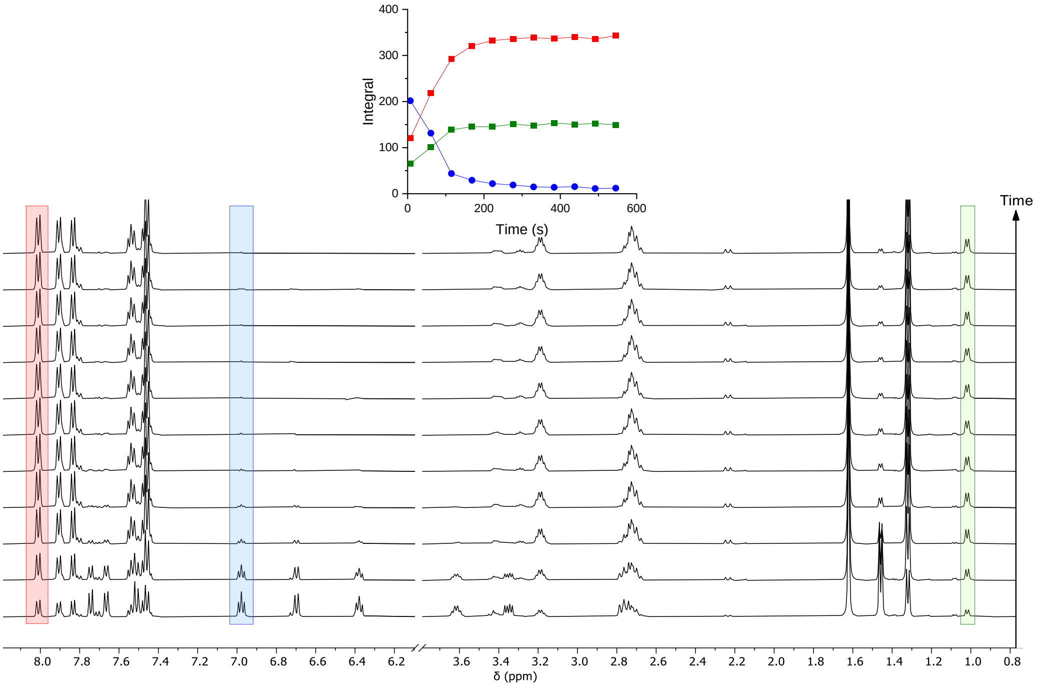

Figure S10. ^{1}H NMR (500 MHz, CH_2Cl_2, –30 °C, spectra every 60 s) monitoring of the conversion of *Z*s-**M2** (blue) to *E*s-**M2** (red) upon in-situ irradiation with 420 nm light at –30 °C. At this temperature, the buildup of metastable intermediate *E*ms-**M2** (green) could not be individually observed due to its fast equilibration with *E*s-**M2** through a fast thermal helix inversion process.

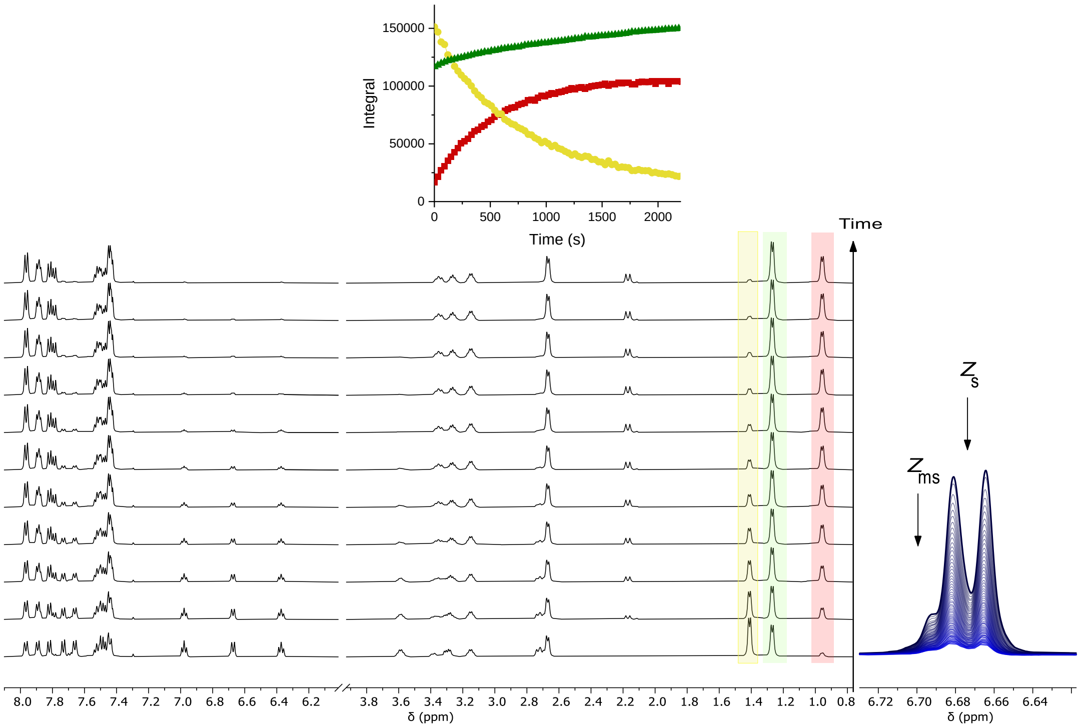

Figure S11. ^{1}H NMR (500 MHz, CH_2Cl_2, –90 °C) monitoring of the isomerization of Z_s-**M2** (yellow) to E_{ms}-**M2** (red) upon in-situ irradiation with 420 nm light at –90 °C showing the partial conversion of Z_{ms}-**M2** to E_s-**M2** (green).

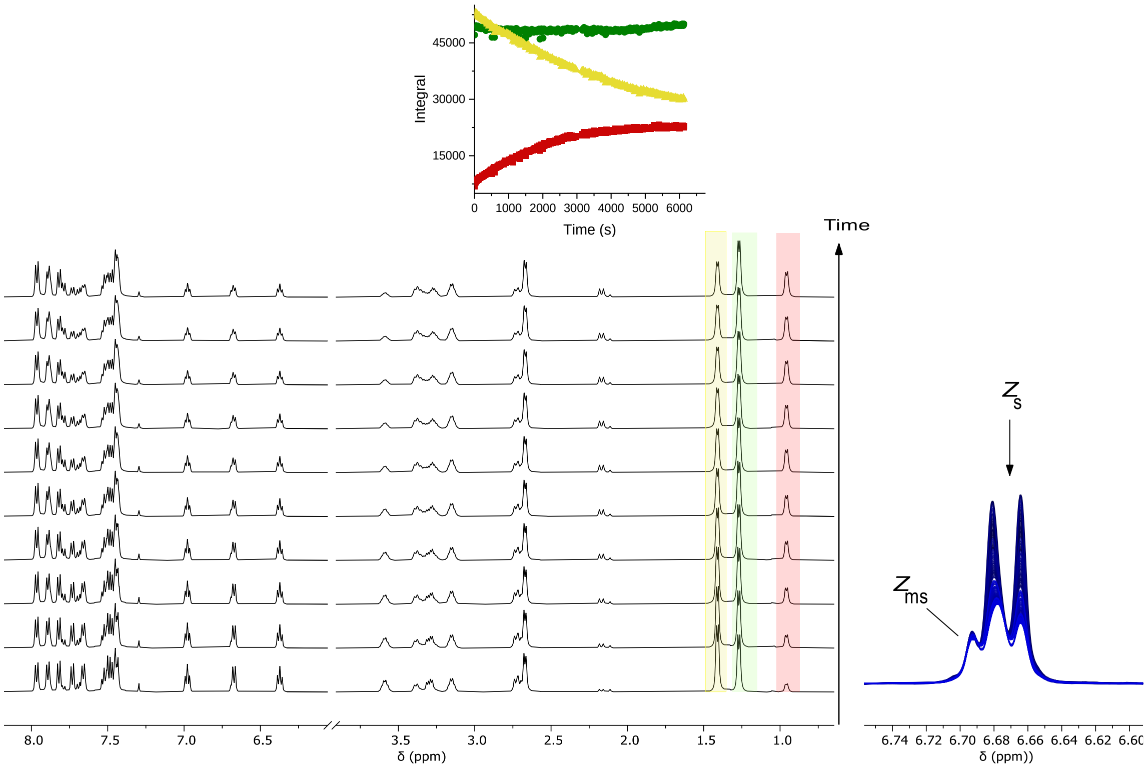

Figure S12. ^{1}H NMR (500 MHz, CH_2Cl_2, –90 °C) monitoring of the isomerization of Z_s-**M2** (yellow) to E_{ms}-**M2** (red) upon in-situ irradiation with 395 nm light at –90 °C showing no conversion of Z_{ms}-**M2** to E_s-**M2** (green).

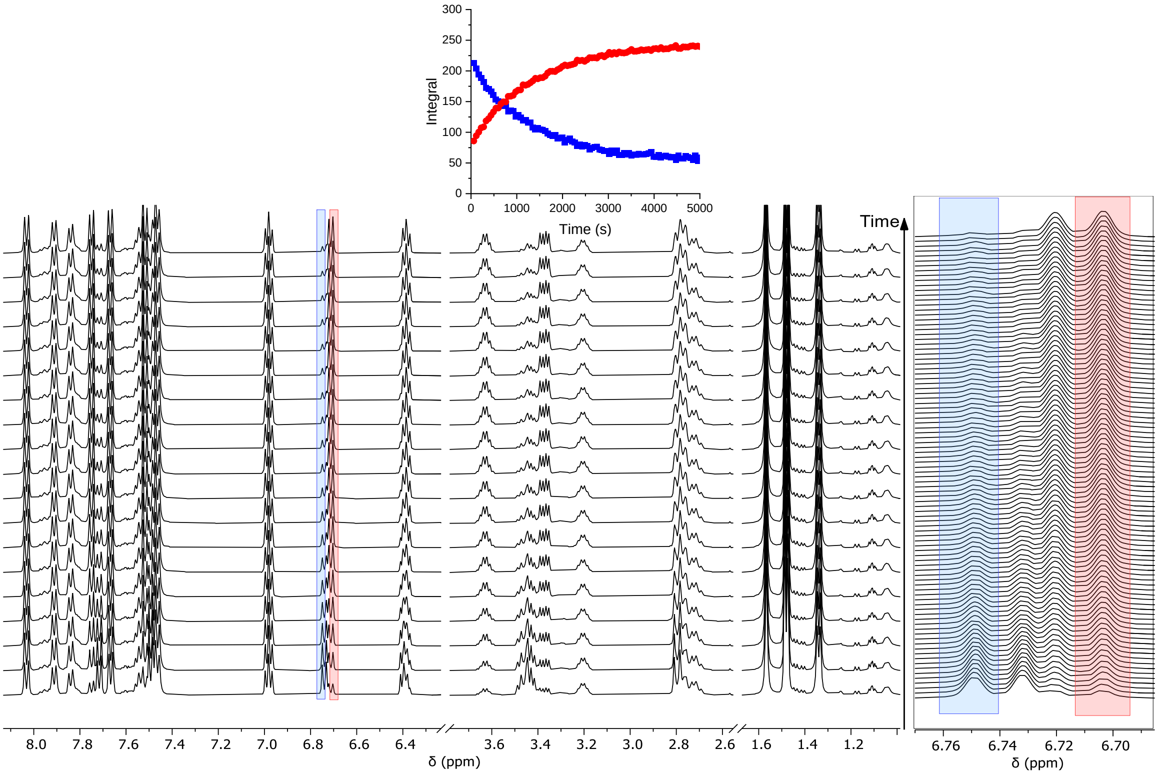

Figure S13. ^{1}H NMR (500 MHz, CH_2Cl_2, 0 °C) monitoring of the relaxation (thermal helix inversion) of Z_{ms}-**M2** (blue) to stable Z_s-**M2** (red) at 0 °C.

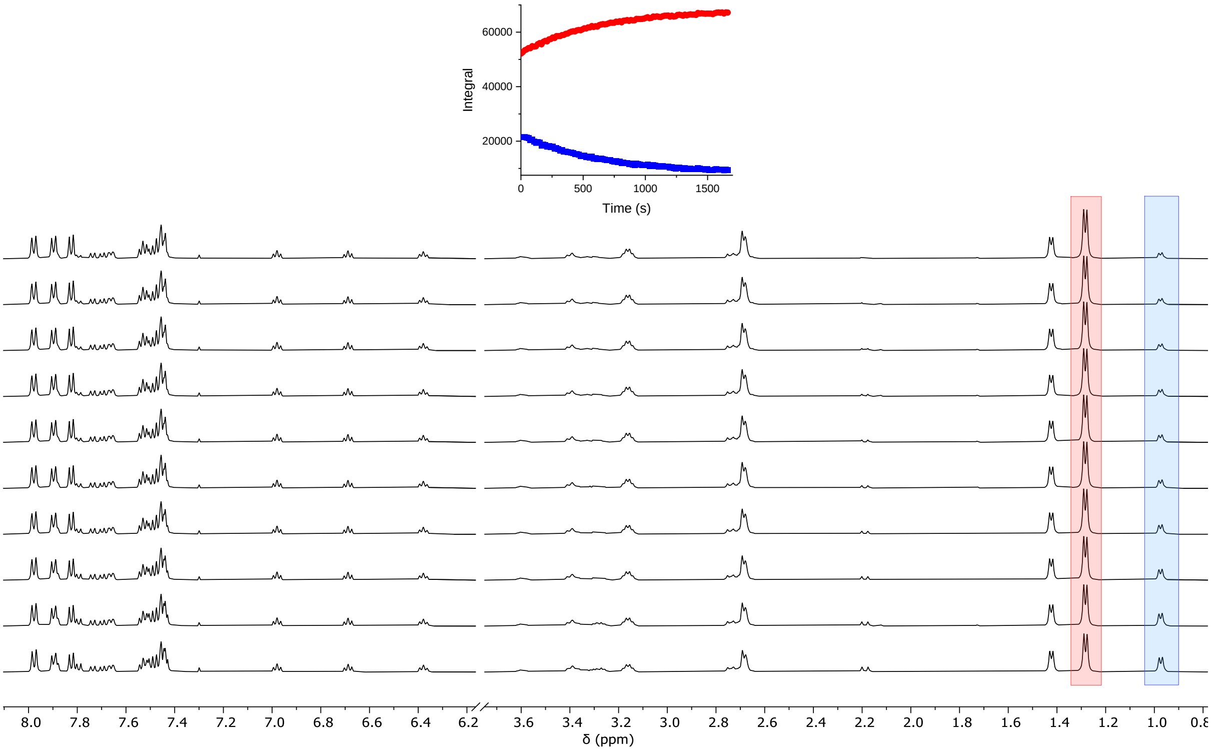

Figure S14. ^{1}H NMR (500 MHz, CH_2Cl_2, –75 °C) monitoring of the relaxation (thermal helix inversion) of E_{ms}-**M2** (blue) to stable E_s-**M2** (red) at –75 °C after irradiation at –90 °C with 395 nm light and increase of the temperature (Figure S12).

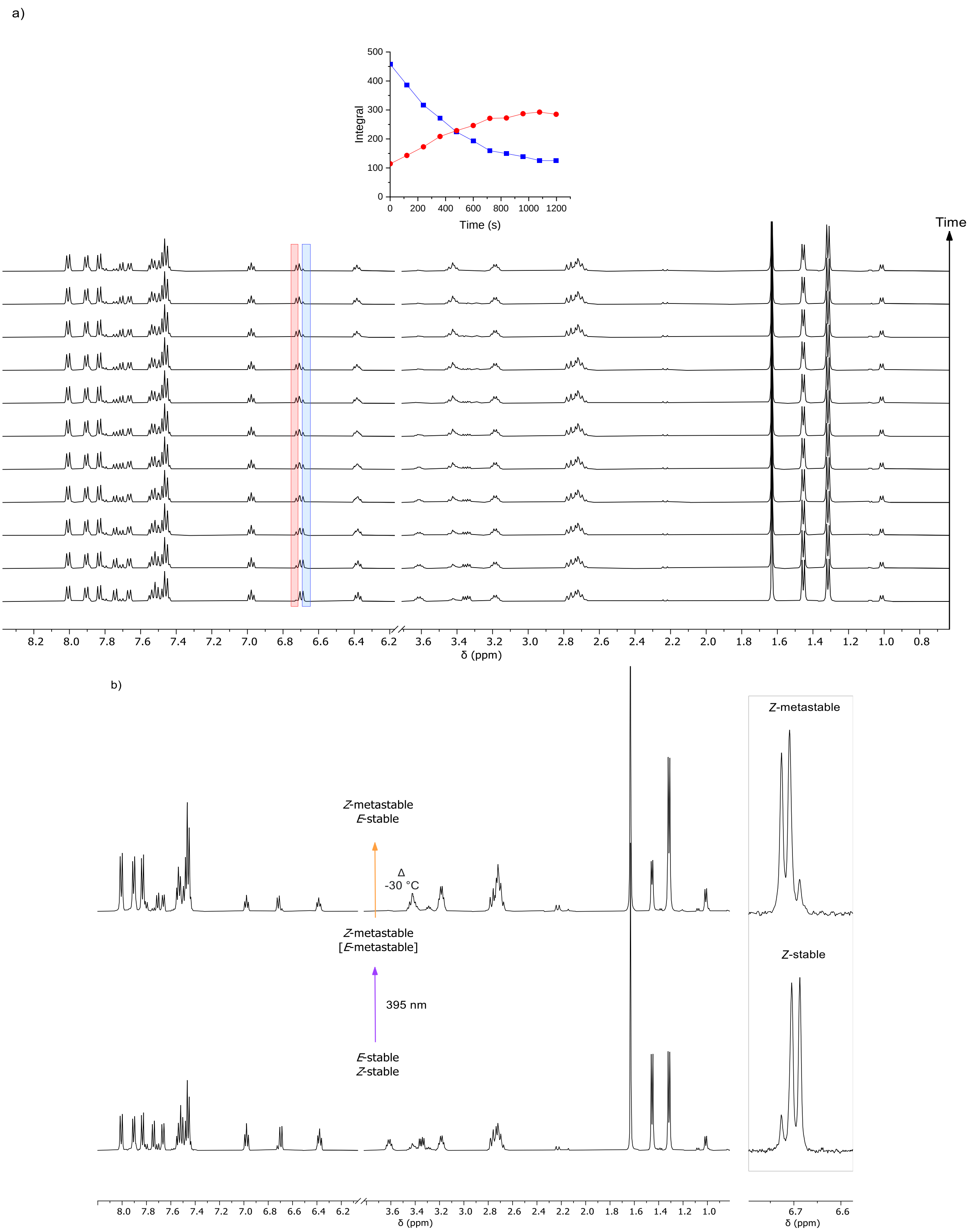

Figure S15. ^{1}H NMR (500 MHz, CH_2Cl_2, –30 °C) evidencing of single-wavelength-induced rotation of **M2**. a) In situ irradiation at –30 °C with 395 nm UV light of an initial mixture composed of E_s-**M2** and Z_s-**M2** resulting in the simultaneous conversion of E_s-**M2** to metastable Z_{ms}-**M2** (red, increasing) and of Z_s-**M2** (blue, decreasing) forming stable E_s-**M2**. Intermediate E_{ms}-**M2** could not be observed at this temperature. b) Enlarged spectra of initial (bottom) and final (top) mixtures.

3. Eyring Analysis

Table S1. Summary of the thermodynamic parameters and half-life times of the thermal helix inversion processes at 20 °C in CD_2Cl_2 determined by variable temperature ^{1}H NMR spectroscopy. $\Delta G^{\ddagger}$ and $\Delta H^{\ddagger}$ are reported in kJ/mol and $\Delta S^{\ddagger}$ in J/(K·mol).

Motor	**ΔG‡** (THI *Z*) [ΔH‡, ΔS‡, $t_{1/2}$]	**ΔG‡** (THI *E*) [ΔH‡, ΔS‡, $t_{1/2}$]
**M1**	94.5±0.5 [65.7, –98.0, 2.1 h]	85.0±0.4 [79.2, –19.8, 2.6 min]
**M2**	84.8±0.7 [64.1, –70.6, 2.4 min]	60.1±0.6 [55.4, –16.1, 6.0 ms]

3.1. Eyring analysis: *Z*-M1

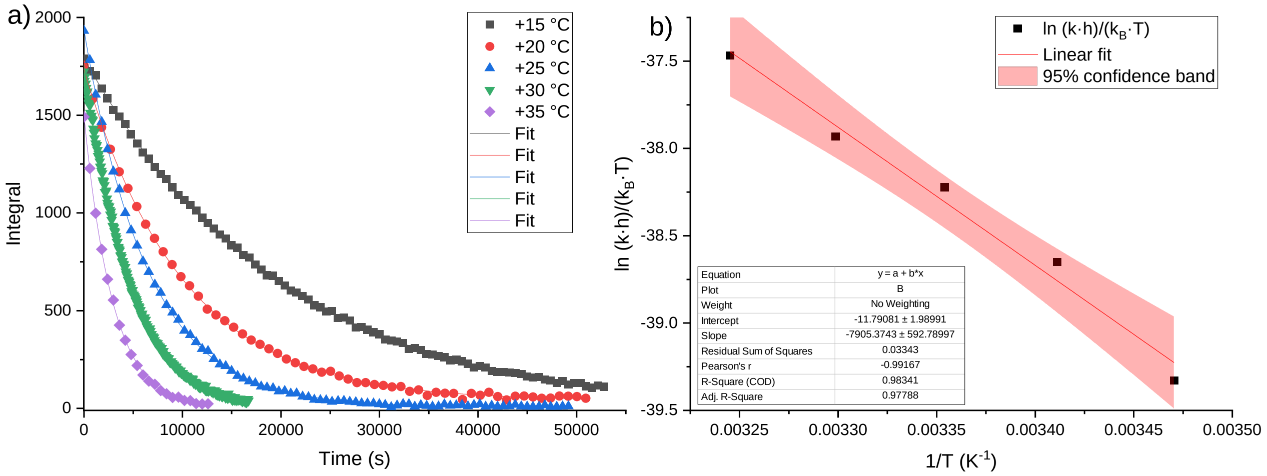

Model	ExpDec1				
Equation	y = A1*exp(-x/t1) + y0				
Plot	+15 °C	+20 °C	+25 °C	+30 °C	+35 °C
y0	-30.51117 ± 3.6530	37.01283 ± 2.3569	5.93795 ± 1.0965	-27.24203 ± 2.211	-7.0008 ± 4.3878
A1	1822.40396 ± 3.362	1697.28047 ± 5.23	1933.01738 ± 3.9	1788.1142 ± 2.96	1510.85635 ± 7.
t1	20043.06925 ± 116.	10007.07856 ± 67.	6404.02595 ± 23.	4710.95369 ± 21.	2913.168 ± 35.1
Reduced Chi-	57.97318	89.57634	48.7964	162.40534	89.14601
R-Square (C	0.99975	0.99955	0.99976	0.99925	0.99956
Adj. R-Squar	0.99974	0.99953	0.99975	0.99924	0.99951

Figure S16. Eyring analysis of thermal helix inversion from metastable Z_{ms}-**M1** to stable Z_s-**M1**. a) Rate constants determination by ^{1}H NMR at various temperatures. The absolute integral value of the doublet at 1.61 ppm was plotted over time and subsequently fitted to an exponential decay model (table). b) Eyring plot.

3.2. Eyring analysis: *E*-M1

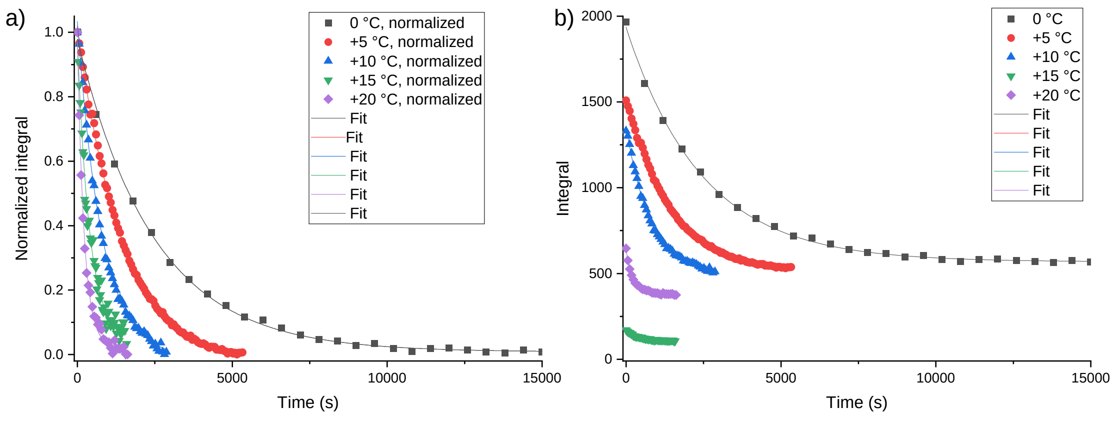

Model	ExpDec1				
Equation	y = A1*exp(-x/t1) + y0				
Plot	0 °C	+5 °C	+10 °C	+15 °C	+20 °C
y0	567.68561 ± 2.17	501.63022 ± 1.92	494.32495 ± 3.72	107.55767 ± 0.61	381.31563 ± 1.3
A1	1369.06153 ± 8.5	1027.78176 ± 2.7	865.86415 ± 5.12	61.97801 ± 1.002	259.21688 ± 4.2
t1	2450.89698 ± 28.	1441.54419 ± 10.	788.39592 ± 12.6	388.99628 ± 15.8	220.60464 ± 7.0
Reduced Chi-	116.60894	44.78521	89.87809	3.56595	25.71863
R-Square (C	0.99886	0.9994	0.9984	0.98743	0.99453
Adj. R-Squar	0.9988	0.99939	0.99833	0.98693	0.99409

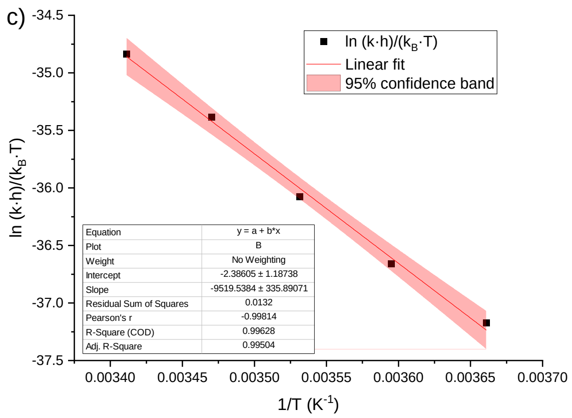

Figure S17. Eyring analysis of thermal helix inversion from metastable E_{ms}-**M1** to stable E_{s}-**M1**. Rate constants determination by ^{1}H NMR at various temperatures. a) Normalized (0 to 1) integral value of the doublet at 1.43 ppm, b) Absolute integral values were plotted over time and subsequently fitted to an exponential decay model (table). c) Eyring plot.

3.3. Eyring analysis: *Z*-M2

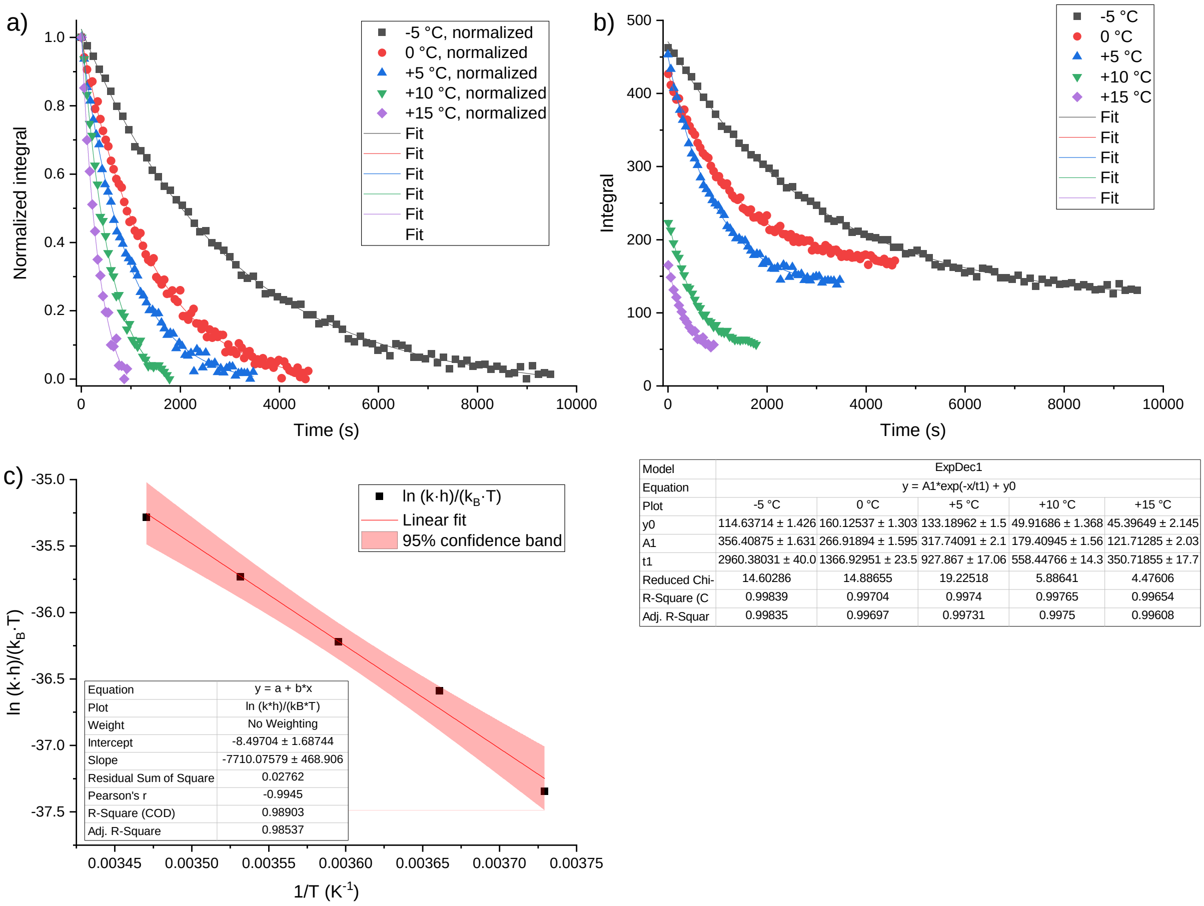

Model	ExpDec1				
Equation	y = A1*exp(-x/t1) + y0				
Plot	-5 °C	0 °C	+5 °C	+10 °C	+15 °C
y0	114.63714 ± 1.426	160.12537 ± 1.303	133.18962 ± 1.5	49.91686 ± 1.368	45.39649 ± 2.145
A1	356.40875 ± 1.631	266.91894 ± 1.595	317.74091 ± 2.1	179.40945 ± 1.56	121.71285 ± 2.03
t1	2960.38031 ± 40.0	1366.92951 ± 23.5	927.867 ± 17.06	558.44766 ± 14.3	350.71855 ± 17.7
Reduced Chi-	14.60286	14.88655	19.22518	5.88641	4.47606
R-Square (C	0.99839	0.99704	0.9974	0.99765	0.99654
Adj. R-Squar	0.99835	0.99697	0.99731	0.9975	0.99608

Figure S18. Eyring analysis of thermal helix inversion from metastable Z_{ms}-**M2** to stable Z_{s}-**M2**. Rate constants determination by ^{1}H NMR at various temperatures. a) Normalized (0 to 1) integral value of the multiplet at 3.45 ppm and b) Absolute integral values were plotted over time and subsequently fitted to an exponential decay model (table). c) Eyring plot.

4. Exchange spectroscopy (VT-EXSY)

4.1. Theoretical model

Consider a two-site exchange between species A and B, given by equation 1

$$A \underset{k_{BA}}{\overset{k_{AB}}{\rightleftharpoons}} B \quad (1)$$

The populations of species A (P_A) and species B (P_B) are given by equations 2 and 3 respectively.

$$P_A = \frac{k_{BA}}{k_{AB}+k_{BA}} \quad (2)$$

$$P_B = \frac{k_{AB}}{k_{AB}+k_{BA}} \quad (3)$$

By solving the Bloch-McConnel equations it follows that the intensities of the auto peaks (diagonal peaks) I_{AA} and I_{BB} and the intensities of the exchange peaks (cross-peaks) I_{AB} and I_{BA} are given by equations 4a-d.[8]

$$I_{AA} = I_A(0)(-(\lambda_2 - a_{11})e^{-\lambda_1\tau_m} + (\lambda_1 - a_{11})e^{-\lambda_2\tau_m})/(\lambda_1 - \lambda_2) \quad (4a)$$

$$I_{BB} = I_B(0)(-(\lambda_2 - a_{22})e^{-\lambda_1\tau_m} + (\lambda_1 - a_{22})e^{-\lambda_2\tau_m})/(\lambda_1 - \lambda_2) \quad (4b)$$

$$I_{AB} = I_A(0)(a_{21}e^{-\lambda_1\tau_m} - a_{21}e^{-\lambda_2\tau_m})/(\lambda_1 - \lambda_2) \quad (4c)$$

$$I_{BA} = I_B(0)(a_{12}e^{-\lambda_1\tau_m} - a_{12}e^{-\lambda_2\tau_m})/(\lambda_1 - \lambda_2) \quad (4d)$$

In these equations $\lambda_{1,2} = \frac{1}{2}\{(a_{11} + a_{22}) \pm [(a_{11} - a_{22})^2 + 4k_{AB}k_{BA}]^{\frac{1}{2}}\}$, $a_{11} = R_A + k_{AB}$, $a_{12} = -k_{BA}$, $a_{21} = -k_{AB}$ and $a_{22} = R_B + k_{BA}$. R_A and R_B are the longitudinal relaxation rates of magnetization for A and B, and $I_A(0)$ and $I_B(0)$ denote the amount of longitudinal magnetization associated with states A and B at the start of the mixing period τ_m.
The activation parameters $\Delta H^‡$ and $\Delta S^‡$ for the exchange reactions can be obtained directly from the temperature dependence of the reaction rates (k) using the following Eyring equation:

$$k = \frac{k_BT}{h}e^{-\frac{\Delta G^‡}{RT}} = \frac{k_BT}{h}e^{-\frac{\Delta H^‡}{RT}+\frac{\Delta S^‡}{R}} \quad (5)$$

Alternatively, this equation can be rewritten in a linearized form, according to equation 6.

$$ln\frac{kh}{k_BT} = -\frac{\Delta H^‡}{R}\frac{1}{T} + \frac{\Delta S^‡}{R} \quad (6)$$

Plotting $ln\frac{kh}{k_BT}$ versus 1/T allows one to perform a linear regression on the data to obtain the enthalpy of activation $\Delta H^‡$ from the negative slope $-\Delta H^‡/R$ and the entropy of activation $\Delta S^‡$ form the y-intercept $\Delta S^‡/R$.

The Gibbs free energy of activation $\Delta G^‡$ at a temperature T can be calculated from the enthalpy and entropy of activation using equation 7.

$$\Delta G^‡ = \Delta H^‡ - T\Delta S^‡ \quad (7)$$

4.2. Exchange spectroscopy

The exchange between the stable and metastable states of motor *E*-**M2** was studied by VT-EXSY spectroscopy.

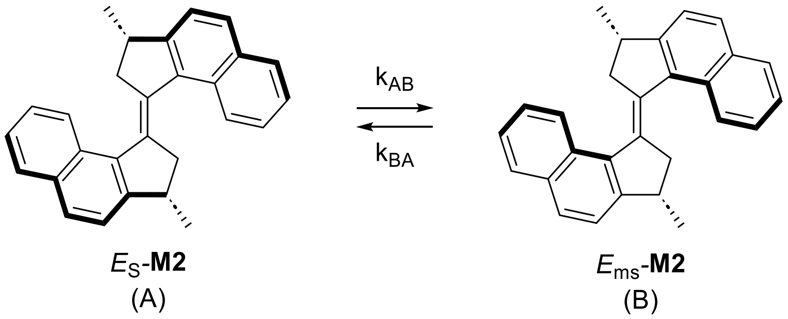

Scheme S1. Exchange reactions between stable and metastable state of motor *E*-**M2**, labeled A and B respectively.

NMR experiments were performed on a Varian Inova 500 MHz (^{1}H NMR frequency) spectrometer. An NMR sample containing solution of motor *E*-**M2** in CD_2Cl_2 was prepared. The chemical exchange between states A and B was monitored by recording a series of 2D homonuclear ^{1}H NOESY spectra with mixing times of 0.01, 0.02, 0.04, 0.08, 0.12, 0.16, 0.20, 0.28, 0.36, 0.44, 0.52, 0.68, 0.84, 1.00, 1.16, 1.48, 1.80 and 2.12 s at 6 different set temperatures of −10, −15, −20, −25, −30 and −35 °C, respectively. A spectral window of 6998 Hz x 1999 Hz was employed, and 4 scans were acquired for each experiment. The chosen relaxation delay was 1.5 s. Figure S19 shows a 1D ^{1}H NMR spectrum of the mixture at −30 °C with the methyl signals of E_s-**M2** and E_{ms}-**M2** that were monitored in the EXSY study labeled A and B, respectively.

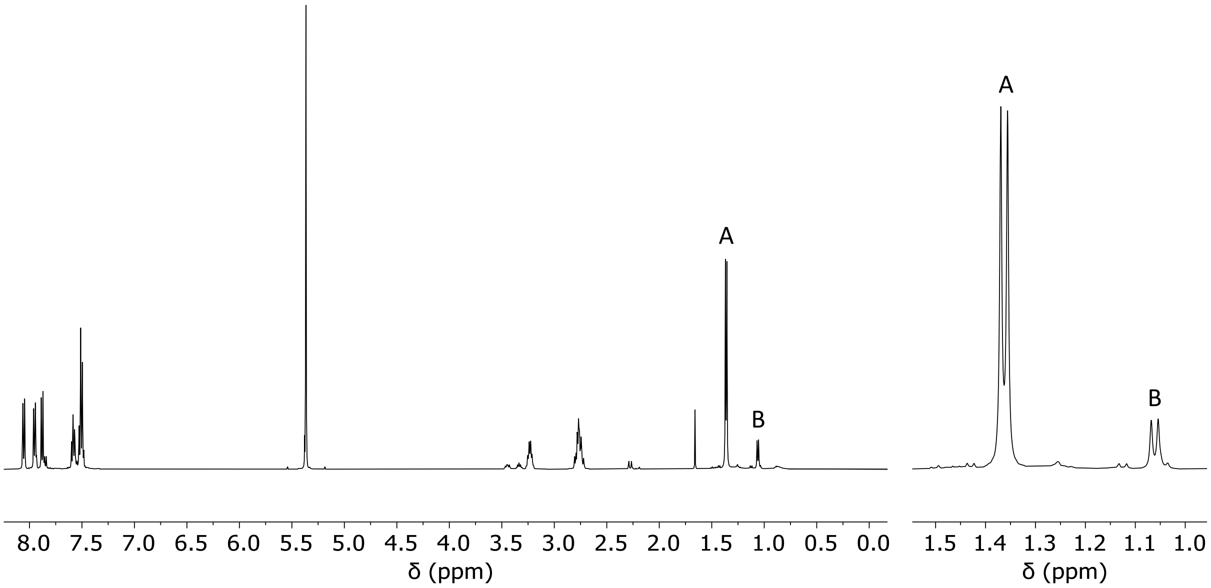

Figure S19. Left: Full ^{1}H NMR spectrum of motor *E*-**M2** (500MHz, CD_2Cl_2, −30 °C). Right: Zoom in on the two exchanging methyl signals that were monitored.

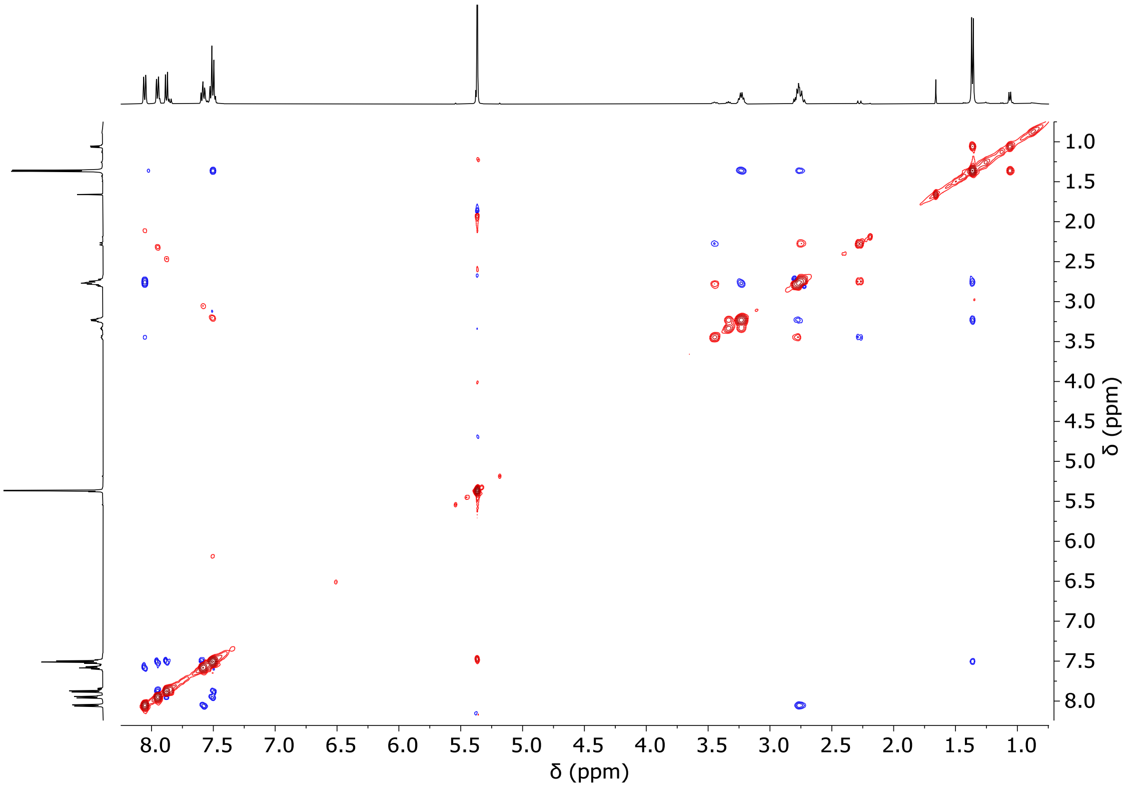

Figure S20. Full 2D NOESY spectrum (500MHz, CD_2Cl_2, −30 °C, 500 ms mixing time) of motor *E*-**M2**, revealing cross-peaks due to exchange (red) and dipolar coupling (blue).

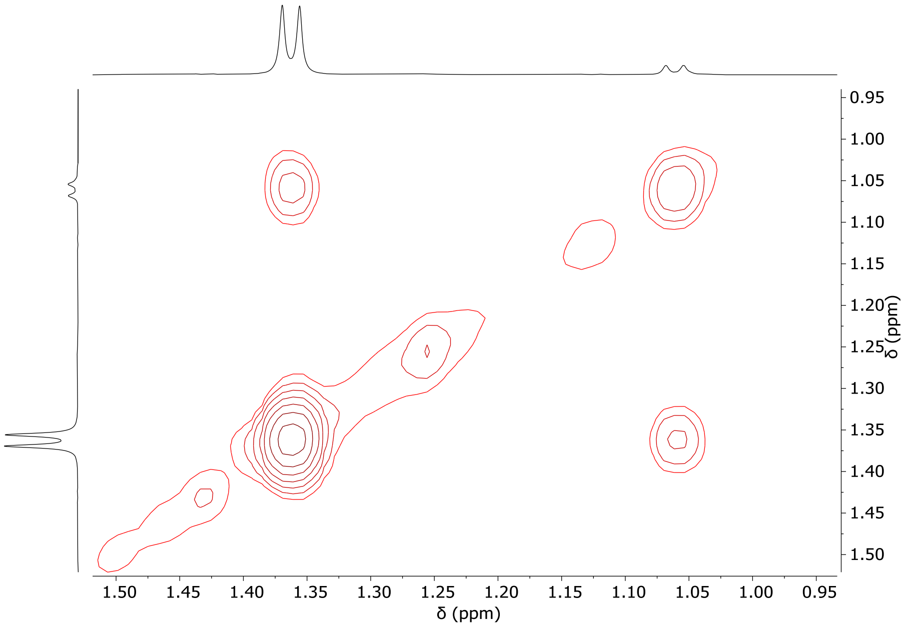

Figure S21. Zoom into the region containing the diagonal peaks and cross-peaks that were integrated in the EXSY study.

The diagonal peaks and the cross peaks were integrated, and their intensity was plotted at each temperature as a function of the mixing time τ_m to obtain the buildup curves shown in Figure S22. These curves were simultaneously fitted using equations 4a-4d. No additional constraints were used in the fitting procedure. Equilibrium constants (i.e. population ratios) were determined from separate 1D ^{1}H NMR spectra. The measured population ratios as well as the calculated rate constants and relaxation rates at each temperature are summarized in Table S2.

Table S2: Summary of the measured population ratios (P_A/P_B) as well as the calculated rate constants (k_{AB} and k_{BA}) and relaxation rates (R_A and R_B) at the different temperatures. Standard errors are given in parentheses.

T (°C)	P_A/P_B	k_{AB} (s^{-1})	k_{BA} (s^{-1})	R_A (s^{-1})	R_B (s^{-1})
−35	6.55	0.076 (0.009)	0.50 (0.05)	2.79 (0.01)	3.09 (0.09)
−30	6.27	0.16 (0.02)	1.02 (0.09)	2.64 (0.02)	2.8 (0.2)
−25	6.13	0.259 (0.009)	1.59 (0.05)	2.32 (0.01)	2.46 (0.08)
−20	6.01	0.48 (0.02)	2.91 (0.09)	2.16 (0.02)	2.0 (0.1)
−15	5.87	0.81 (0.03)	4.8 (0.1)	1.20 (0.02)	1.8 (0.2)
−10	5.89	1.40 (0.04)	8.3 (0.2)	1.92 (0.04)	1.3 (0.2)

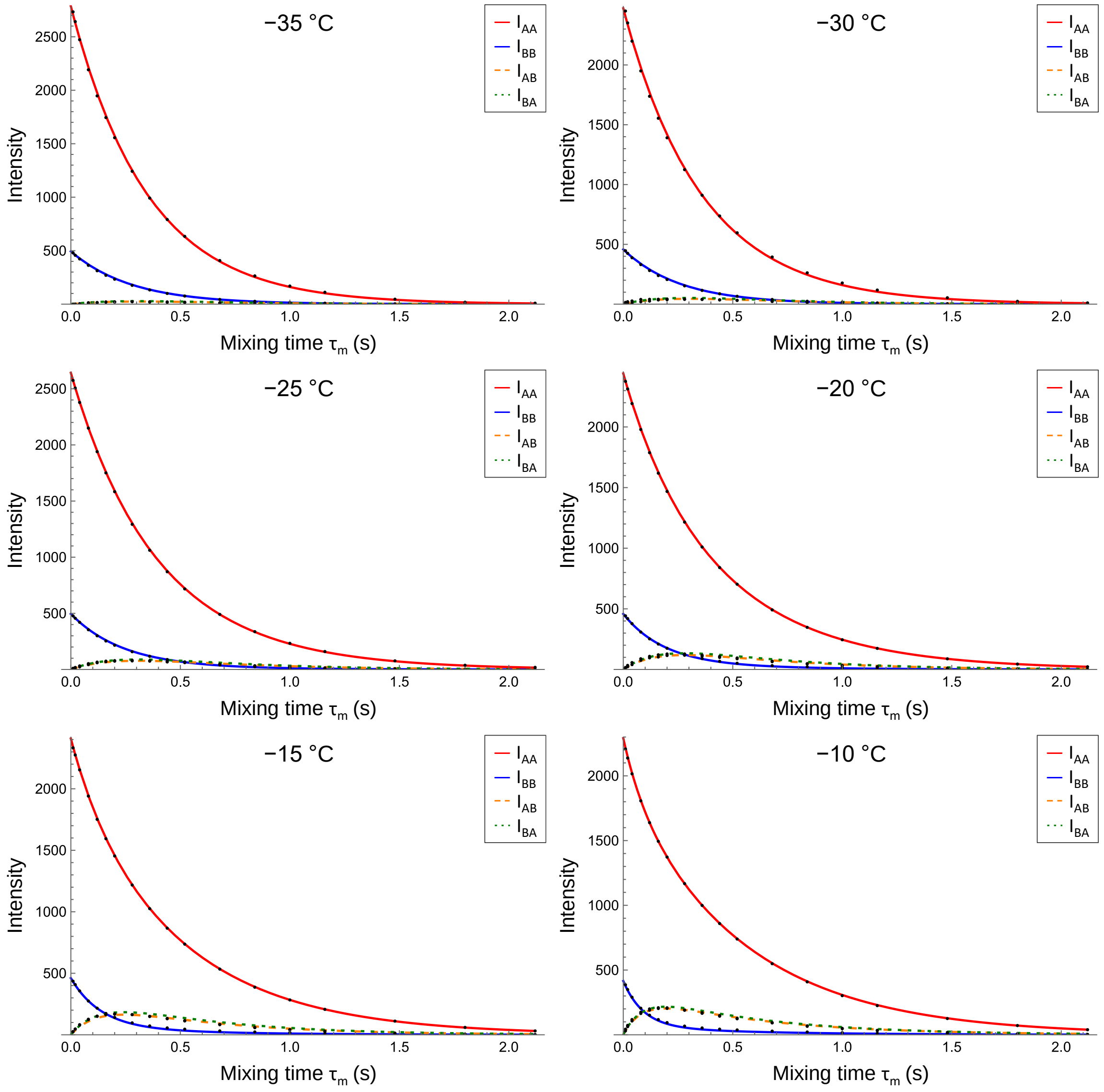

Figure S22. Buildup curves showing the intensities of the diagonal peaks and cross-peaks as a function of mixing time τ_m at the different temperatures.

The obtained rate constants for the forward and backward reactions were plotted against 1/T and fitted using the linearized Eyring equation (equation 6), see Figure S23. The obtained activation parameters and the corresponding values at 20 °C of the Gibbs free energy of activation, half-life and lifetime are summarized in Table S3.

Table S3: Calculated activation parameters corresponding to the exchange reactions A → B and B → A. Standard errors are given in parentheses.

	**ΔH‡ (kJ·mol⁻¹)**	**ΔS‡ (J·mol⁻¹·K⁻¹)**	**ΔG‡ (20 °C) (kJ·mol⁻¹)**	**$t_{1/2}$ (20 °C) (s)**	**τ (20 °C) (s)**
**A → B**	58 (1)	−22 (5)	64.1 (0.2)	3.0 x 10^{-2}	4.4 x 10^{-2}
**B → A**	55 (1)	−16 (5)	60.1 (0.2)	6.0 x 10^{-3}	8.7 x 10^{-3}

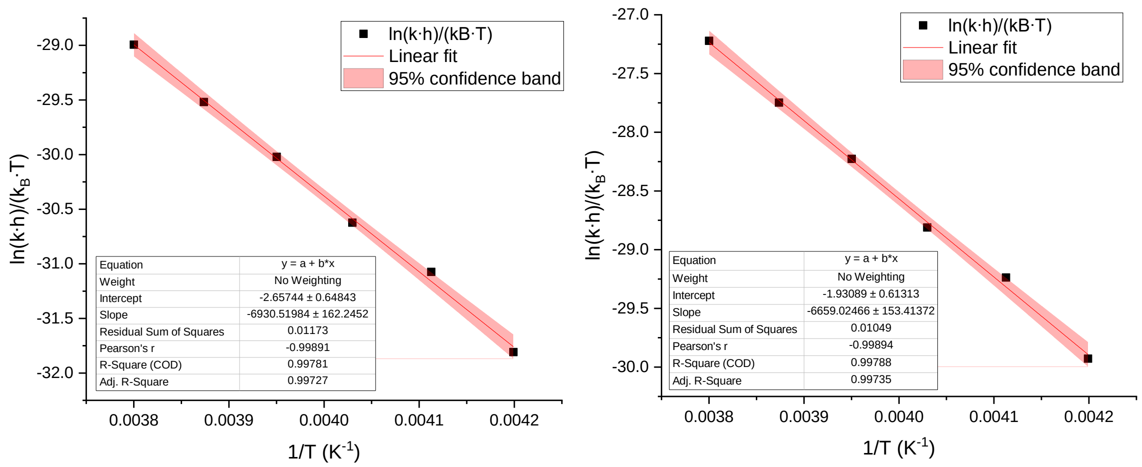

Figure S23. Eyring plots for the exchange reactions A → B (left) and B → A (right).

5. Variable-temperature UV-vis spectroscopy

In the following section, only the main isomers will be included in the legend on the spectra. A more detailed composition of every state can be found in the ^{1}H NMR section.

For both **M1** and **M2**, the same enantiomer as the one used for the NMR section was studied. The same notation omitting stereodescriptors for clarity will be used.

5.1. UV-vis study of M1

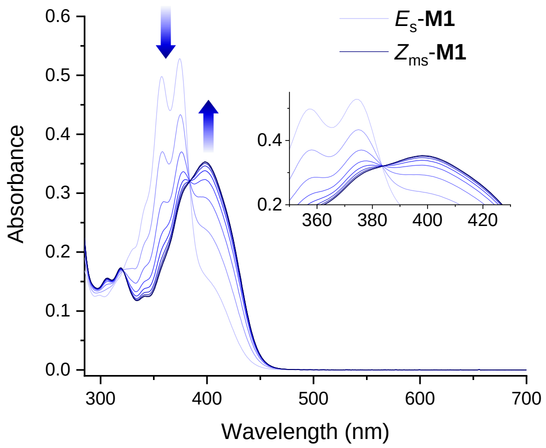

Figure S24. Irradiation of a pure sample of E_s-**M1** with 365 nm UV light, followed by UV-vis spectroscopy (CH_2Cl_2, ~25 µM, 20 °C). The obtained PSS_{365} is composed of a majority of Z_{ms}-**M1**.

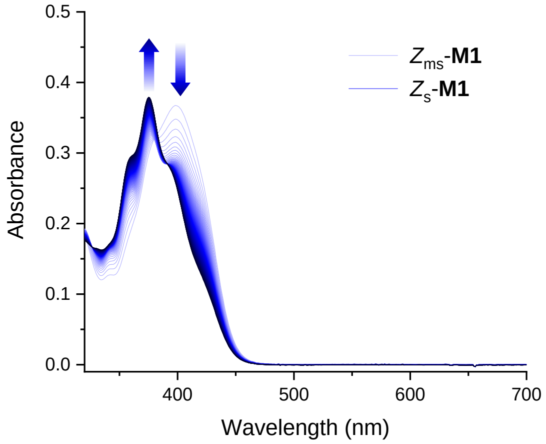

Figure S25. Part of thermal helix inversion of Z_{ms}-**M1** to Z_s-**M1** followed by UV-vis spectroscopy (CH_2Cl_2, ~25 µM, 20 °C, 320 nm band-pass filter). Monitoring of this thermal step over extended periods by UV-vis induced photobleaching and back-switching, attributed to the rather high intensity of the spectrophotometer light source, leading to a complicated observed process as underlined by the absence of a clean isosbestic point even in a degassed solution and when using a 320 nm band-pass filter. ^{1}H NMR was found to be more efficient to monitor this process (see Figure S4).

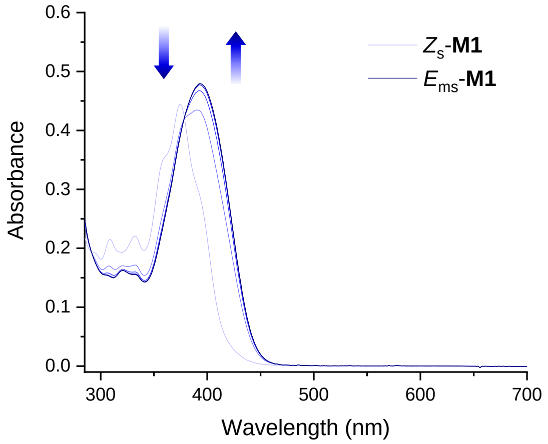

Figure S26. Irradiation of a sample of *Z*s-**M1** with 365 nm UV light, followed by UV-vis spectroscopy (CH_2Cl_2, ~25 µM, 0 °C). The obtained PSS_{365} is composed of a majority of metastable *E*$_{ms}$-**M1**.

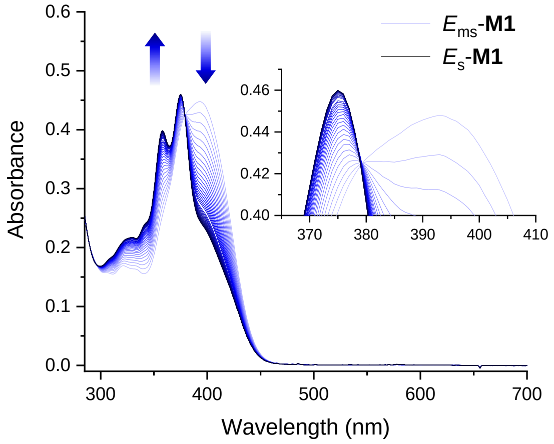

Figure S27. Thermal helix inversion of *E*$_{ms}$-**M1** to stable *E*$_{s}$-**M1** followed by UV-vVis spectroscopy (CH_2Cl_2, ~25 µM, 0 °C).

5.2. UV-vis study of M2

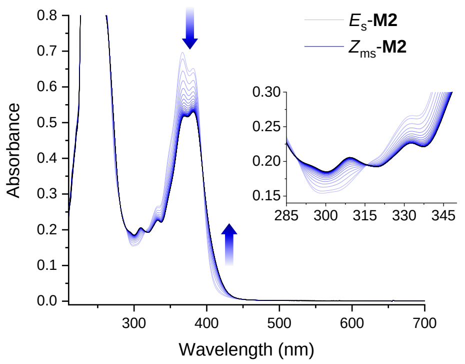

Figure S28. Irradiation of a pure sample of E_s-**M2** with 365 nm UV light, followed by UV-vis spectroscopy (CH_2Cl_2, ~30 µM, 20 °C). The obtained PSS_{365} is composed of a majority of metastable Z_{ms}-**M2**.

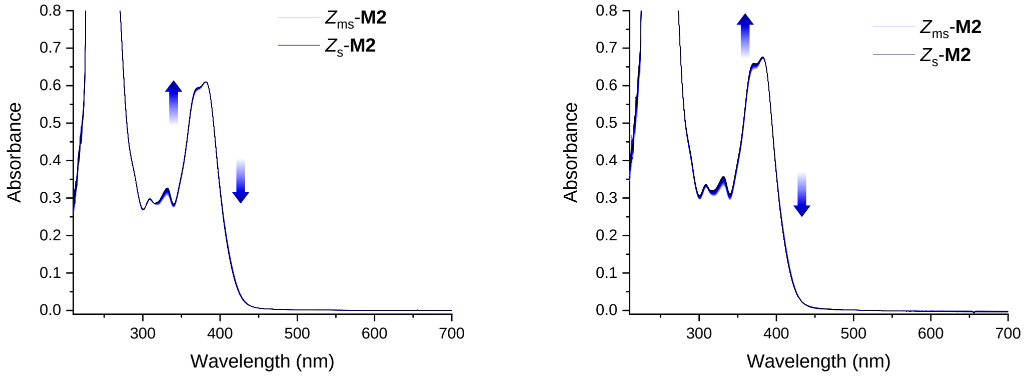

Figure S29. Thermal relaxation of PSS_{365} composed of a majority of Z_{ms}-**M2** to a mixture composed of a majority E_s-**M2** followed by UV-vis spectroscopy (CH_2Cl_2, ~30 µM). Only very small changes were observed at 20 °C (left) or 0 °C (right).

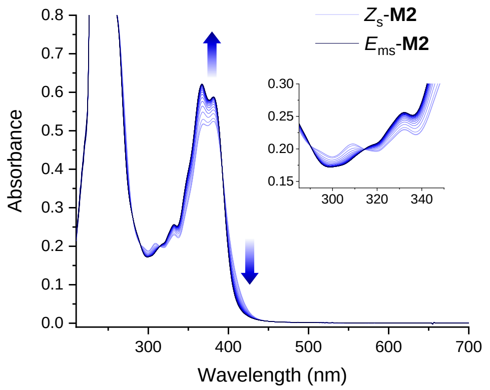

Figure S30. Irradiation of a sample of stable containing a majority of Z_s-**M2** with 420 nm light, followed by UV-vis spectroscopy (CH_2Cl_2, ~30 µM, 20 °C). Due to its rapid decay to E_s-**M2**, metastable E_{ms}-**M2** could not be observed. As clean isosbestic points could be observed the thermal helix inversion step is assumed to result in very small changes in the UV-vis spectrum, as observed for the THI of the Z isomer.

6. Variable-temperature circular dichroism

6.1. CD study of M1

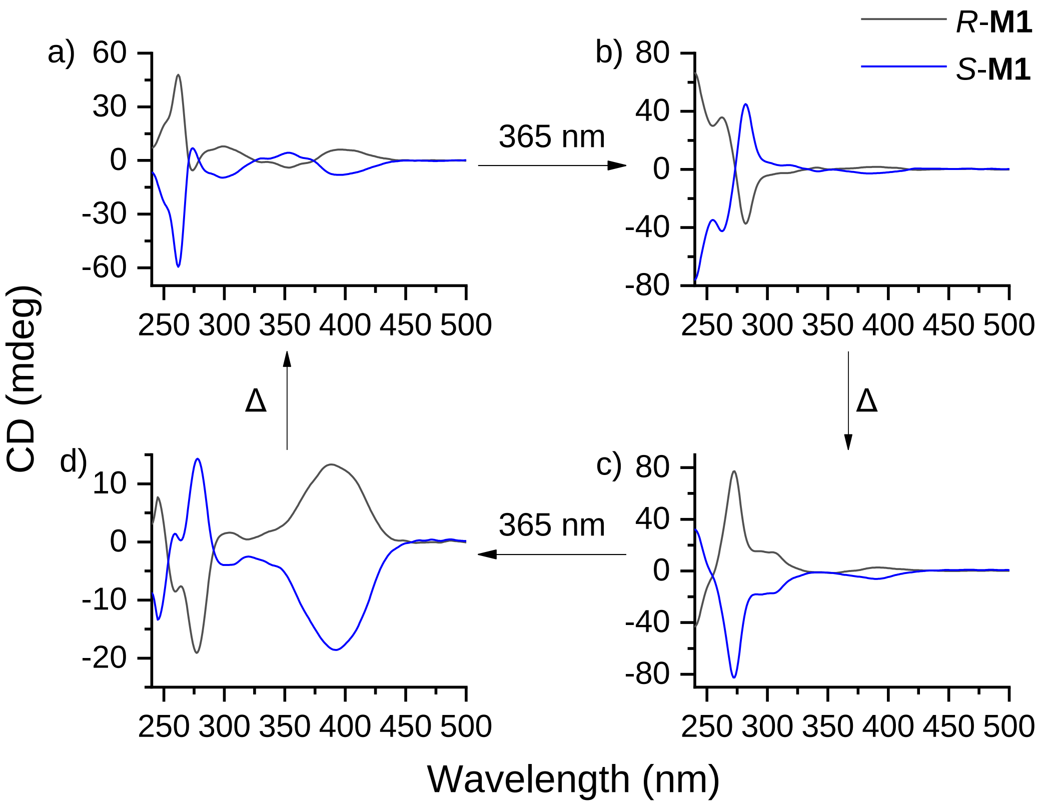

Figure S31. Circular dichroism spectra (CH_2Cl_2, ~30 µM) of the four-step rotation cycle of (2*R*,2'*R*,3*R*,3'*R*)-**M1** (black) and (2*S*,2'*S*,3*S*,3'*S*)-**M1** (blue). a) Initial samples of pure *E*-**M1** at 20 °C, b) PSSs composed of a majority of metastable Z_{ms}-**M1** obtained after irradiation with 365 nm UV light at 20 °C, c) Mixtures composed of a majority of Z_s-**M1** obtained after full relaxation (over ca. 16 h) at 20 °C of the previous samples, measured at –20 °C (identical spectra were obtained at +20 °C), d) PSSs composed of a majority of metastable E_{ms}-**M1** obtained after irradiation with 365 nm UV light at –20 °C.

6.2. CD study of M2

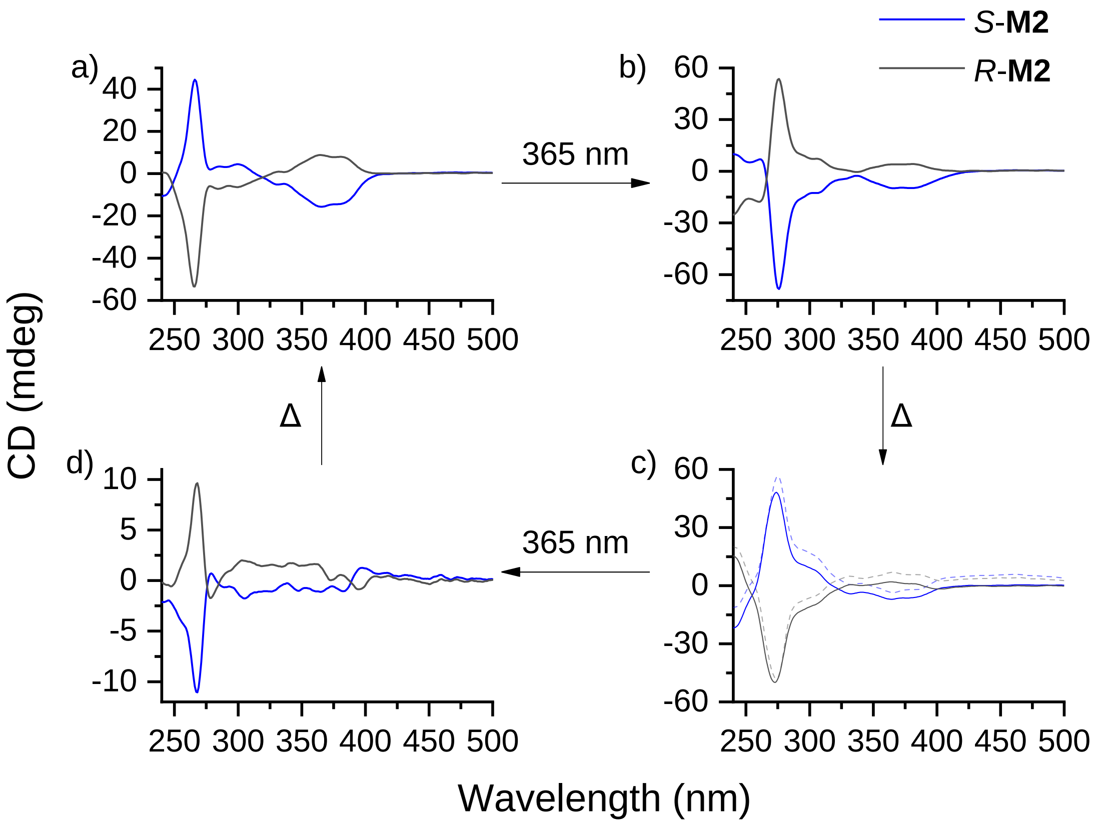

Figure S32. Circular dichroism spectra (CH_2Cl_2, ~30 µM) of the four-step rotation cycle of (3*S*,3'*S*)-**M2** (blue) and (3*R*,3'*R*)-**M2** (black). a) Initial samples of pure *E*-**M2** at –20 °C, b) PSSs composed of a majority of metastable Z_{ms}-**M2** obtained after irradiation with 365 nm UV light at –20 °C, c) Mixtures composed of a majority of stable Z_s-**M2** obtained after full relaxation of the previous samples by heating to +30 °C for 5 min before cooling back down to –20 °C (dashed line: same sample measured at –90 °C), d) PSSs composed of a majority of E_{ms}-**M2** obtained after irradiation with 365 nm UV light at –90 °C. These last samples required to apply a linear baseline correction due to more important thermal drifting and reflects the composition of the mixture after ca. 30 s of irradiation and the time needed to record the spectra (30 s to 1 min).

7. Computational analysis

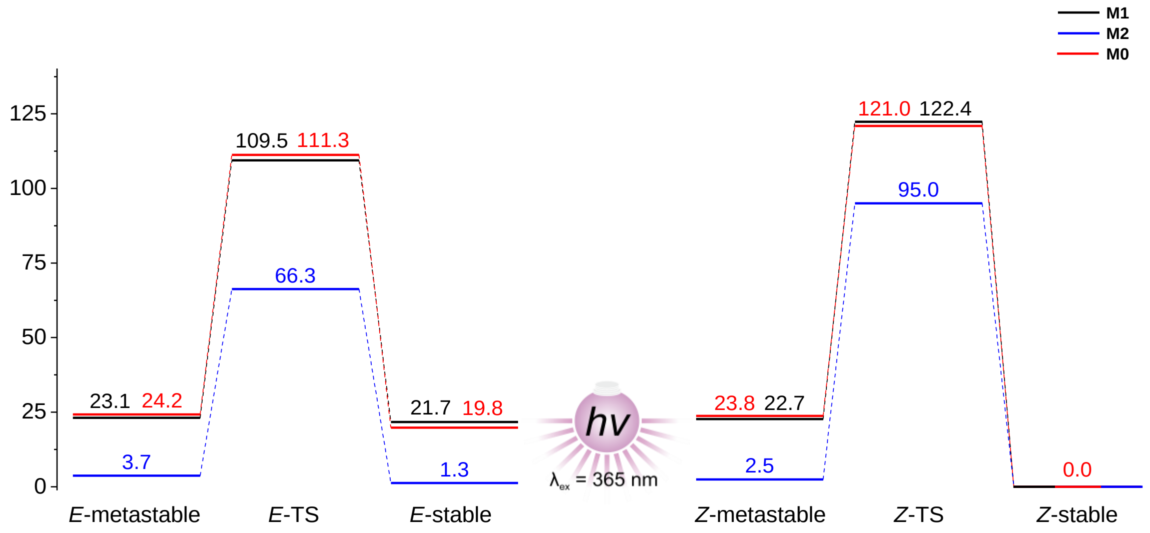

Figure S33. Calculated mechanistic pathways for the thermal helix inversions of molecular motors **M0**, **M1** and **M2** at the r^{2}SCAN-3c/CPCM(CH_2Cl_2) level of theory. Energies are given in kJ/mol.

	S-*E*-**M1**	*S*-*E*-**M2**
Stable	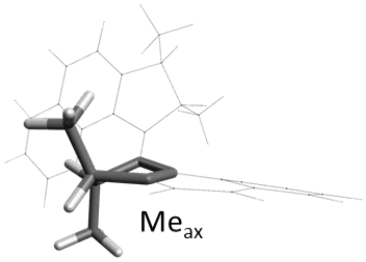	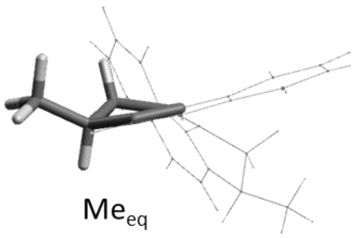
Metastable	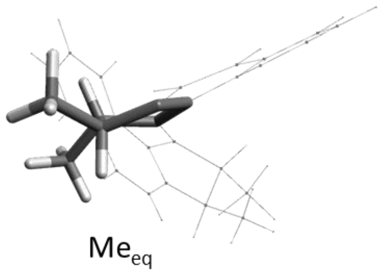	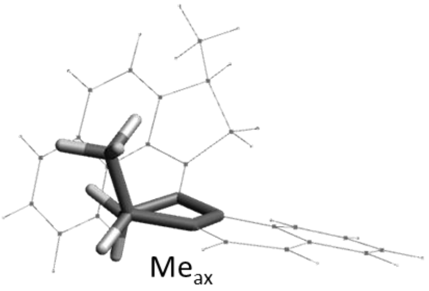

Figure S34. Comparison of the orientation of the stereogenic methyl groups between the stable and metastable *E* isomers of motors *S*-**M1** and *S*-**M2**. The same behavior is observed for the cis isomer.

8. X-ray structural data

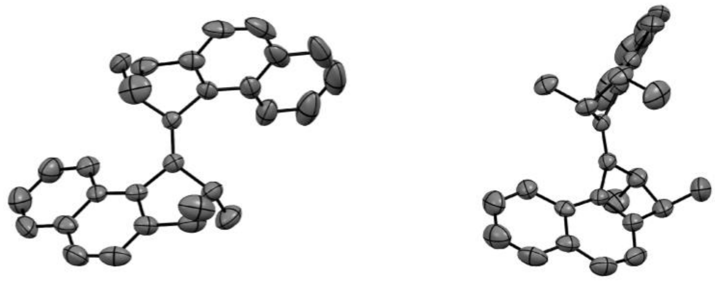

Table S4. Crystal data and structure refinement for (2*R*,2'*R*,3*R*,3'*R*)-(*P*,*P*)-*E*-**M1**.

Identification code	mo_CNS_YG_AG17_0m_a
Empirical formula	$C_{30}H_{28}$
Formula weight	388.52
Temperature/K	293
Crystal system	orthorhombic
Space group	$P2_12_12_1$
a/Å	10.508(2)
b/Å	13.106(3)
c/Å	16.098(4)
α/°	90
β/°	90
γ/°	90
Volume/Å^3	2217.1(10)
Z	4
ρ_{calc}g/cm^3	1.164
μ/mm^{-1}	0.065
F(000)	832.0
Crystal size/mm^3	0.496 × 0.17 × 0.126
Radiation	MoKα (λ = 0.71073)
2Θ range for data collection/°	6.218 to 58.26
Index ranges	-14 ≤ h ≤ 14, -17 ≤ k ≤ 17, -22 ≤ l ≤ 22
Reflections collected	65935
Independent reflections	5956 [R_{int} = 0.1341, R_{sigma} = 0.0445]
Data/restraints/parameters	5956/0/275
Goodness-of-fit on F^2	1.320
Final R indexes [I>=2σ (I)]	R_1 = 0.1143, wR_2 = 0.1812
Final R indexes [all data]	R_1 = 0.1593, wR_2 = 0.2027
Largest diff. peak/hole / e Å^{-3}	0.28/-0.21

Ellipsoids are set at 50% and hydrogen atoms are omitted for clarity.
No A- or B-level alerts were raised by CheckCIF for the fully refined structure.

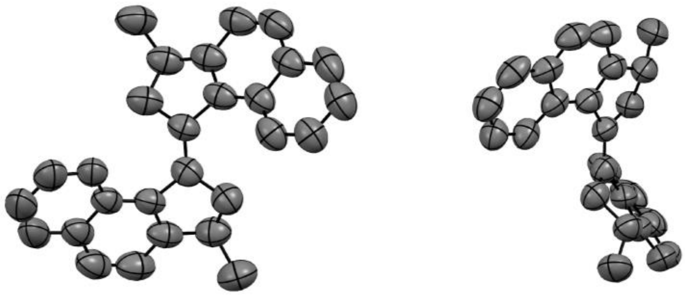

Table S5. Crystal data and structure refinement for (3*R*,3'*R*)-(*M*,*M*)-*E*-**M2**.

Identification code	cu_CNS_YG_AG_28_2_0ma_a
Empirical formula	$C_{28}H_{24}$
Formula weight	360.47
Temperature/K	293
Crystal system	hexagonal
Space group	$P6_1$
a/Å	19.570(4)
b/Å	19.570(4)
c/Å	9.999(3)
α/°	90
β/°	90
γ/°	120
Volume/Å^3	3316.2(18)
Z	6
ρ_{calc}g/cm^3	1.083
μ/mm^{-1}	0.458
F(000)	1152.0
Crystal size/mm^3	0.557 × 0.176 × 0.104
Radiation	CuKα (λ = 1.54178)
2Θ range for data collection/°	9.038 to 145.572
Index ranges	-24 ≤ h ≤ 24, -24 ≤ k ≤ 23, -11 ≤ l ≤ 9
Reflections collected	64235
Independent reflections	4069 [R_{int} = 0.1607, R_{sigma} = 0.0840]
Data/restraints/parameters	4069/1/256
Goodness-of-fit on F^2	0.949
Final R indexes [I>=2σ (I)]	R_1 = 0.0872, wR_2 = 0.1882
Final R indexes [all data]	R_1 = 0.1376, wR_2 = 0.2242
Largest diff. peak/hole / e Å^{-3}	0.57/-0.38

Ellipsoids are set at 50% and hydrogen atoms are omitted for clarity.

The following A- and B-level ALERTS were generated:

Alert level A

PLAT601_ALERT_2_A Unit Cell Contains Solvent Accessible VOIDS of 398 Ang**3

Author response: The compound crystallizes in the highly symmetric hexagonal space group $P6_1$ with large solvent-accessible voids, as a consequence of channels being formed along the screw axis in the *c* direction of the crystal. These voids are likely occupied by disordered solvent molecules.

Alert level B

PLAT340_ALERT_3_B Low Bond Precision on C-C Bonds .... 0.01048 Ang.

Author response: This crystal was not strongly diffracting, and the measurement was performed at room temperature, due to technical limitations. Therefore, the bond precision on the C-C bonds is lower.

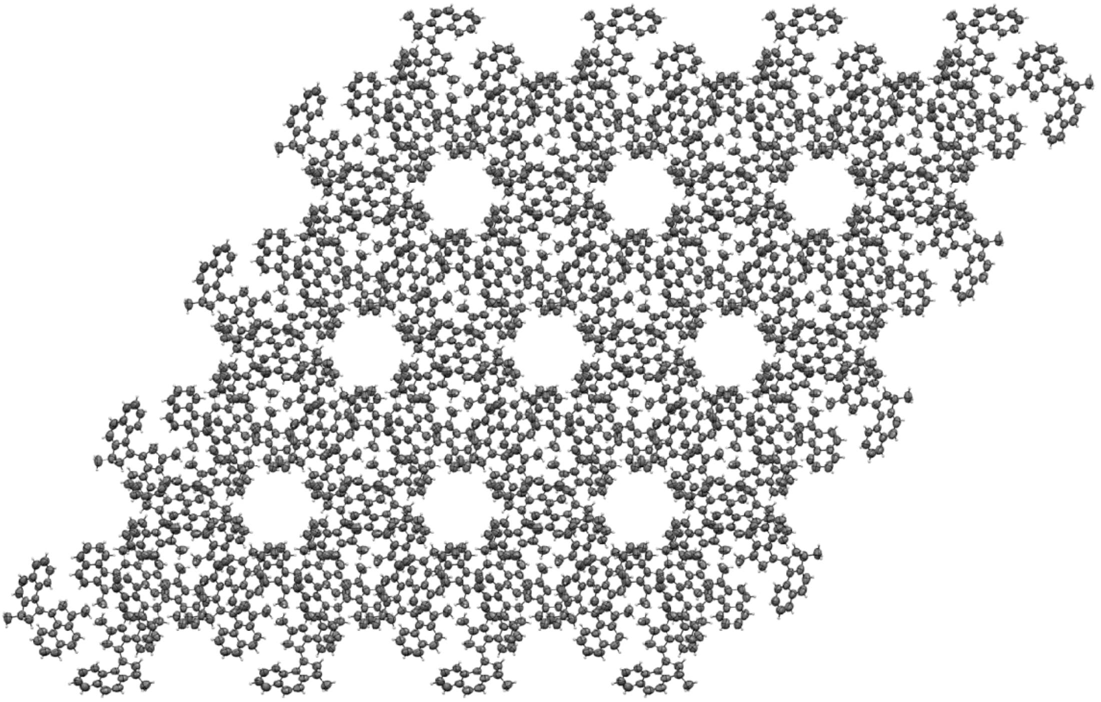

Figure S35. Crystal packing of (3R,3'R)-(M,M)-*E*-**M2** along the *c* axis showing the solvent-accessible channels.

9. HPLC chromatograms

Attributions were performed by injection of *E*/*Z* mixtures which did not allow for baseline separation (and *ee* determination) but allowed to attribute the minor isomeric peaks.

<Chromatogram>

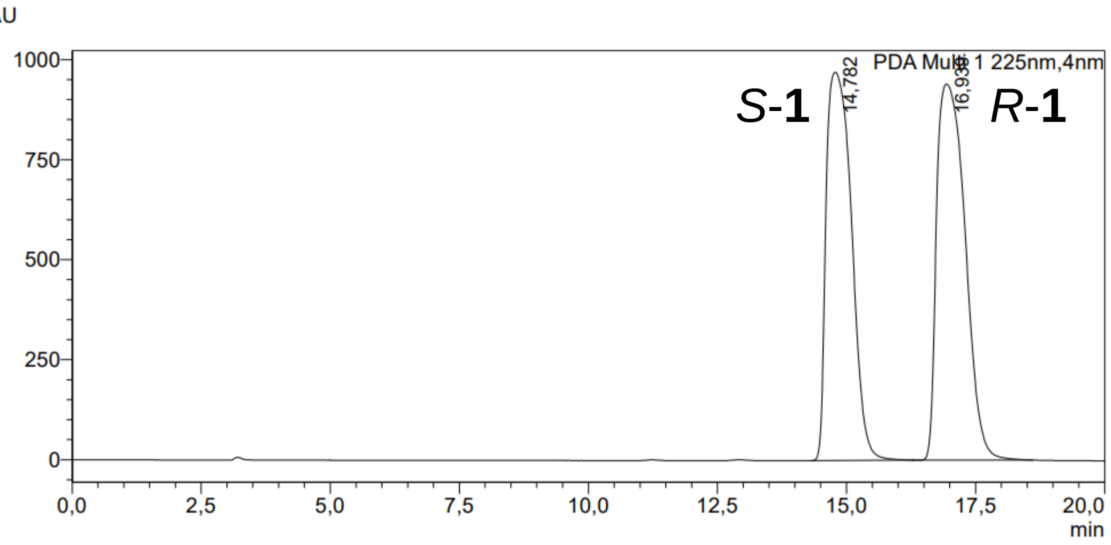

<Peak Table>

PDA Ch1 225nm

Peak#	Ret. Time	Area%
1	14,782	47,918
2	16,939	52,082
Total		100,000

HPLC chromatogram of *rac*-**1** (Chiralcel OB-H, *n*-heptane/2-propanol 97:3, 1.0 mL/min).

<Chromatogram>

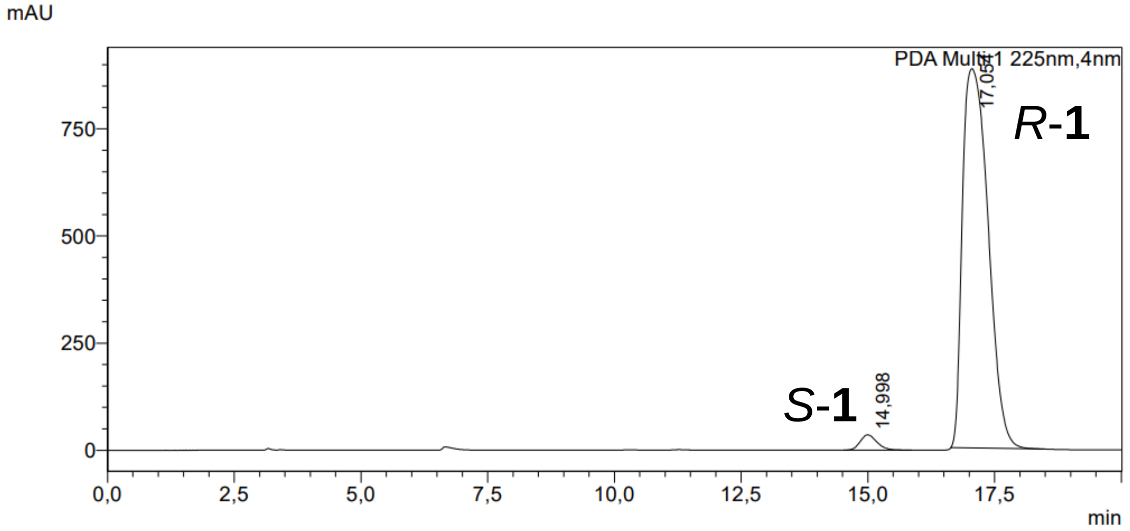

<Peak Table>

PDA Ch1 225nm

Peak#	Ret. Time	Area%
1	14,998	2,544
2	17,054	97,456
Total		100,000

HPLC chromatogram of *R*-**1** (Chiralcel OB-H, *n*-heptane/2-propanol 97:3, 1.0 mL/min).

<Chromatogram>

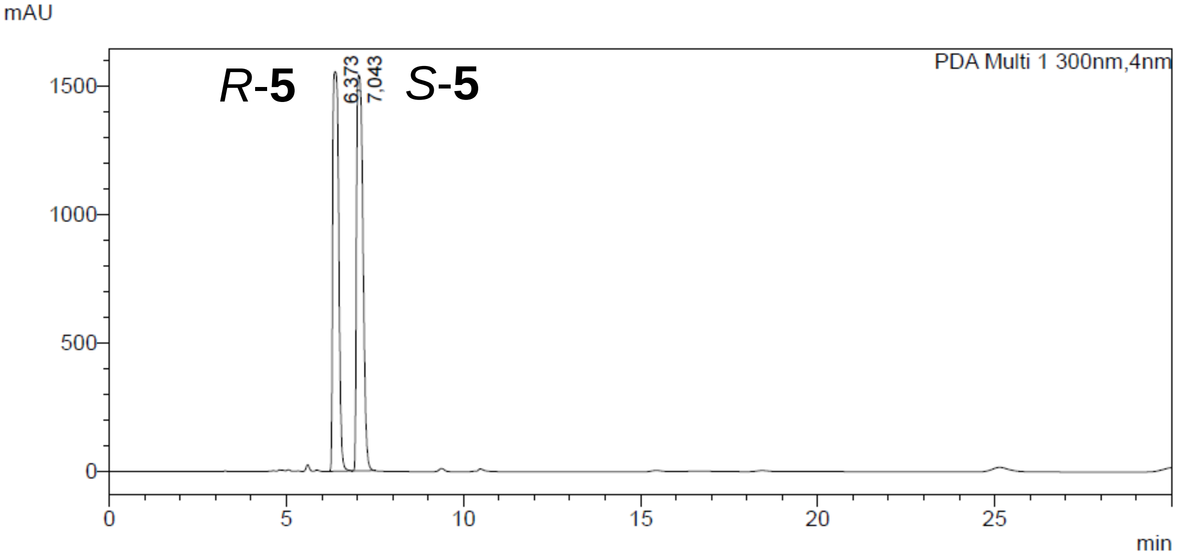

<Peak Table>

PDA Ch1 300nm

Peak#	Ret. Time	Area%
1	6,373	48,371
2	7,043	51,629
Total		100,000

HPLC chromatogram of a mixture of *R*-**5** and *S*-**5** (Chiralcel OJ-H, *n*-heptane/2-propanol 95:5, 1.0 mL/min).

<Chromatogram>

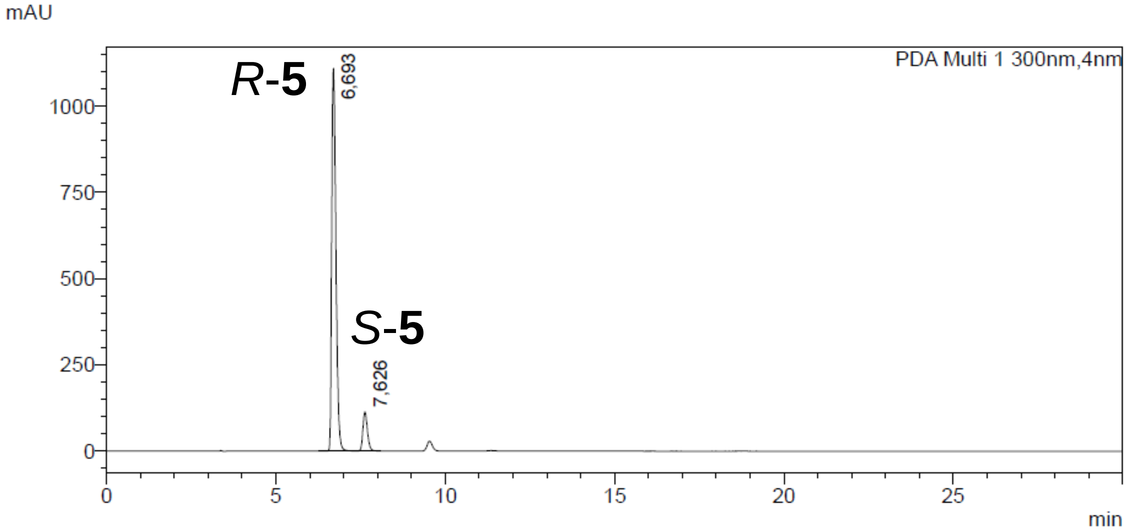

<Peak Table>

PDA Ch1 300nm

Peak#	Ret. Time	Area%
1	6,693	90,084
2	7,626	9,916
Total		100,000

HPLC chromatogram of *R*-**5** (Chiralcel OJ-H, *n*-heptane/2-propanol 95:5, 1.0 mL/min).

<Chromatogram>

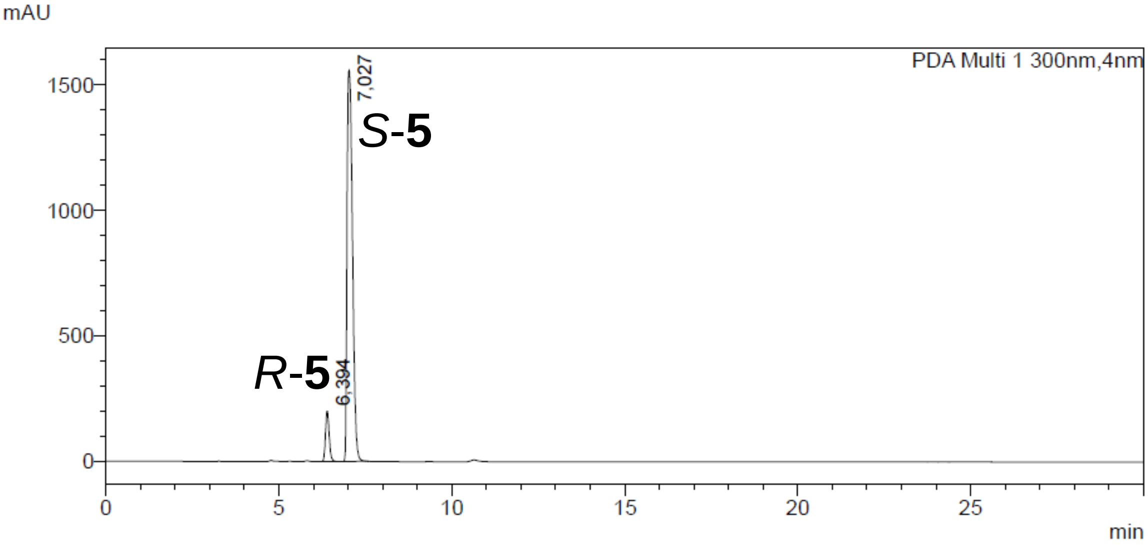

<Peak Table>

PDA Ch1 300nm

Peak#	Ret. Time	Area%
1	6,394	7,955
2	7,027	92,045
Total		100,000

HPLC chromatogram of *S*-**5** (Chiralcel OJ-H, *n*-heptane/2-propanol 95:5, 1.0 mL/min).

<Chromatogram>

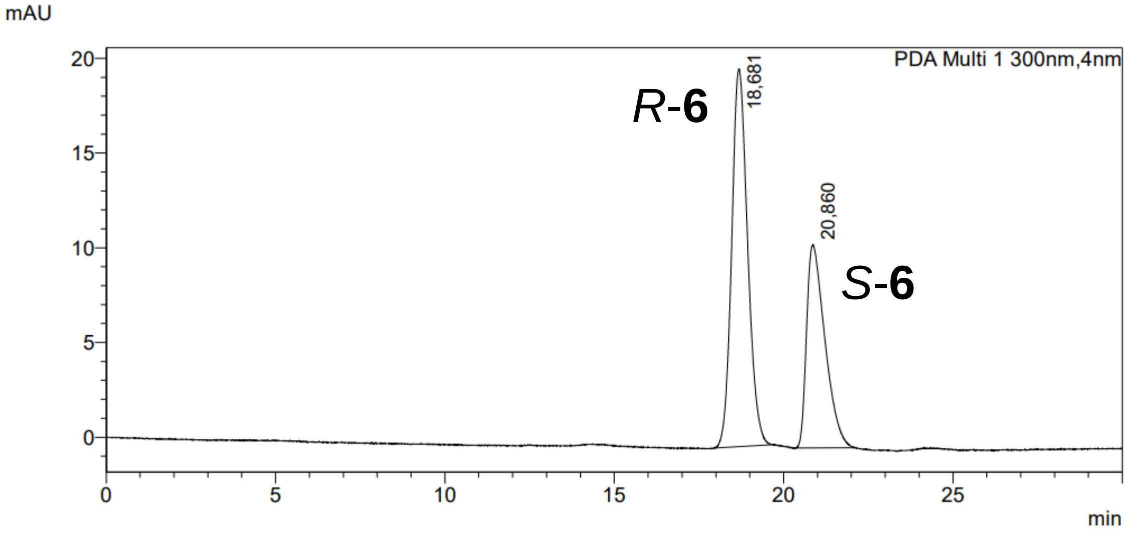

<Peak Table>

PDA Ch1 300nm

Peak#	Ret. Time	Area	Area%
1	18,681	651203	61,655
2	20,860	404994	38,345
Total		1056198	100,000

HPLC chromatogram of a mixture of *R*-**6** and *S*-**6** (Chiralcel OD-H, *n*-heptane/2-propanol 99.4:0.6, 0.6 mL/min).

<Chromatogram>

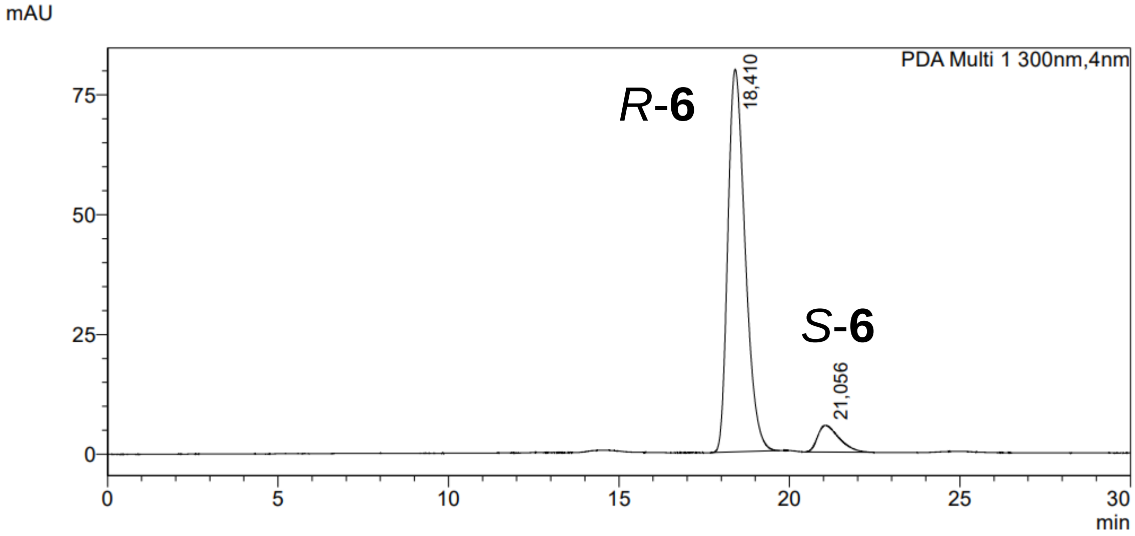

<Peak Table>

PDA Ch1 300nm

Peak#	Ret. Time	Area	Area%
1	18,410	2813124	92,030
2	21,056	243622	7,970
Total		3056746	100,000

HPLC chromatogram of *R*-**6** (Chiralcel OD-H, *n*-heptane/2-propanol 99.4:0.6, 0.6 mL/min).

<Chromatogram>

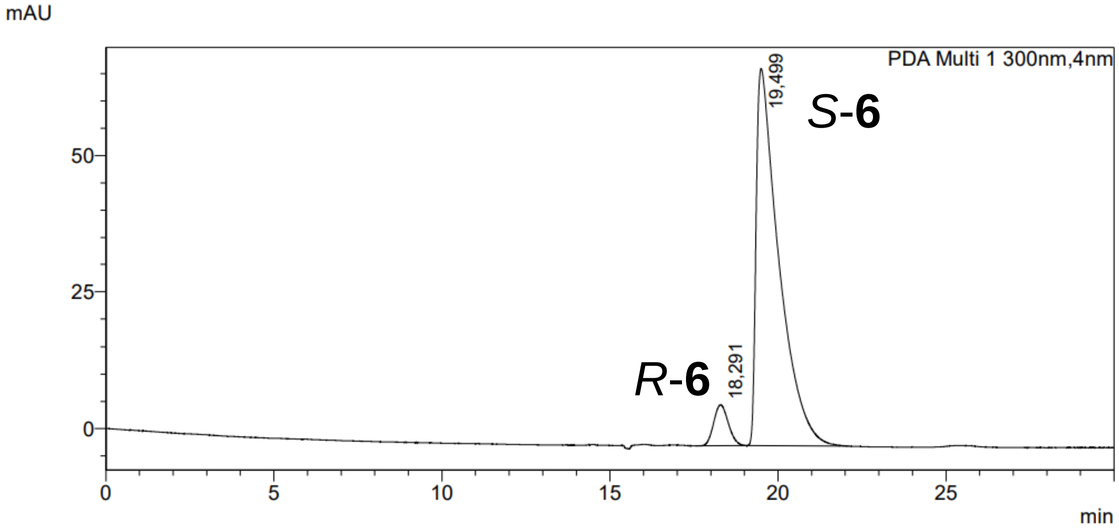

<Peak Table>

PDA Ch1 300nm

Peak#	Ret. Time	Area	Area%
1	18,291	225338	6,502
2	19,499	3240451	93,498
Total		3465790	100,000

HPLC chromatogram of *S*-**6** (Chiralcel OD-H, *n*-heptane/2-propanol 99.4:0.6, 0.6 mL/min).

<Chromatogram>

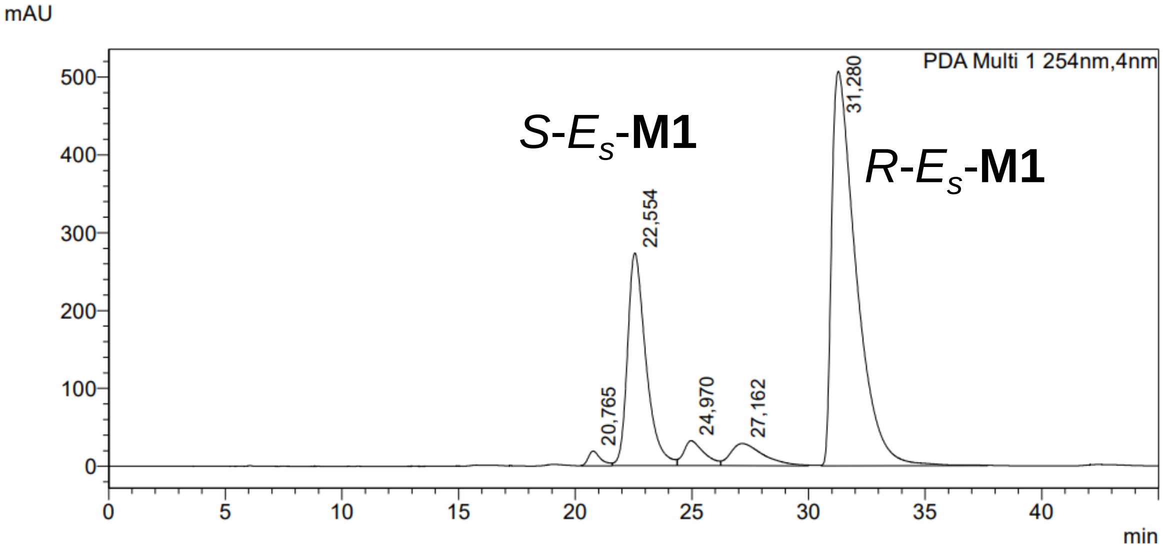

<Peak Table>

PDA Ch1 254nm

Peak#	Ret. Time	Area%
1	20,765	1,229
2	22,554	26,122
3	24,970	3,355
4	27,162	4,787
5	31,280	64,507
Total		100,000

HPLC chromatogram of a mixture of *R*-*E*-**M1** and *S*-*E*-**M1** (Chiralcel OD-H, *n*-heptane, 0.5 mL/min).

<Chromatogram>

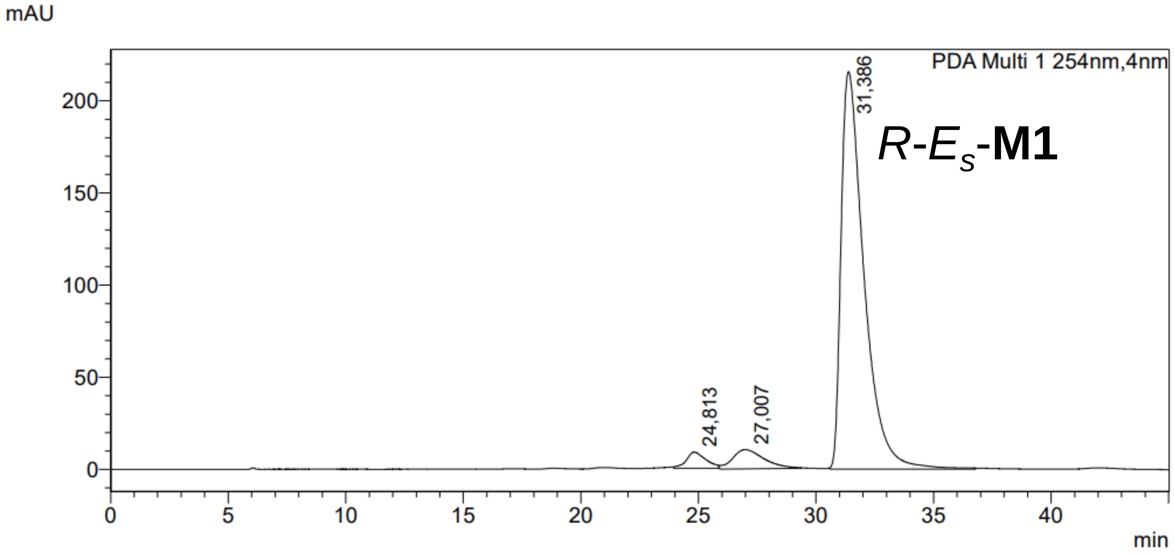

<Peak Table>

PDA Ch1 254nm

Peak#	Ret. Time	Area%
1	24,813	3,130
2	27,007	6,029
3	31,386	90,841
Total		100,000

HPLC chromatogram of *R*-*E*-**M1** (Chiralcel OD-H, *n*-heptane, 0.5 mL/min).

<Chromatogram>

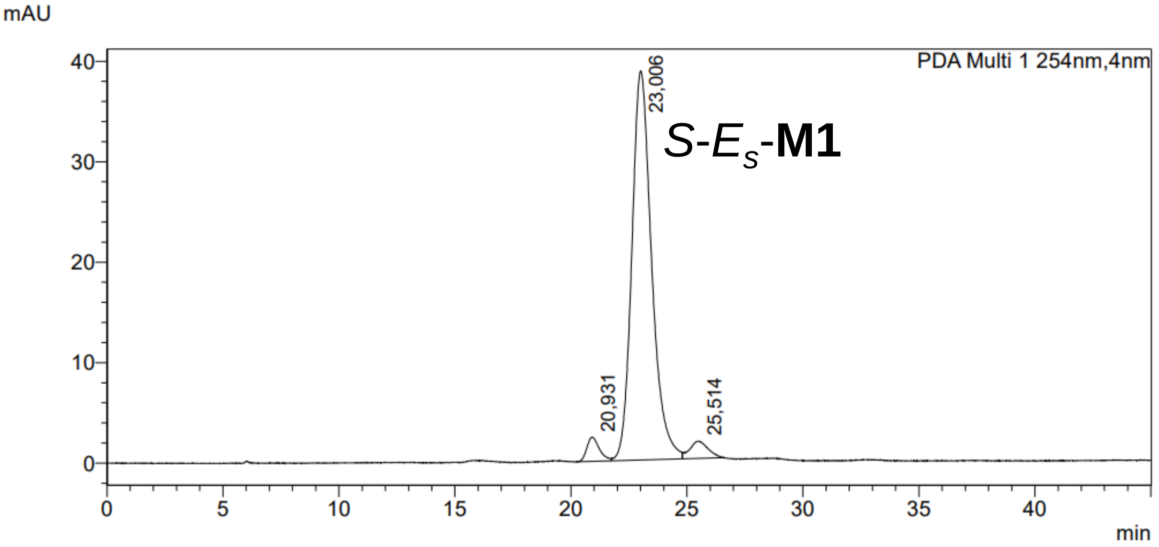

<Peak Table>

PDA Ch1 254nm

Peak#	Ret. Time	Area%
1	20,931	3,616
2	23,006	92,634
3	25,514	3,751
Total		100,000

HPLC chromatogram of *S*-*E*-**M1** (Chiralcel OD-H, *n*-heptane, 0.5 mL/min).

<Chromatogram>

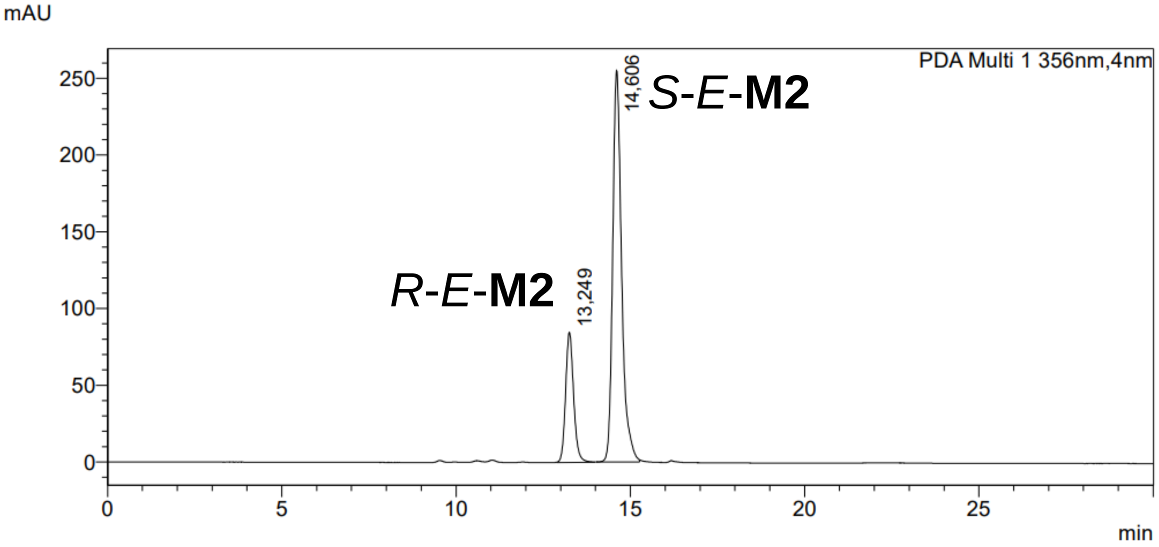

<Peak Table>

PDA Ch1 356nm

Peak#	Ret. Time	Area%
1	13,249	23,042
2	14,606	76,958
Total		100,000

HPLC chromatogram of a mixture of *R*-*E*-**M2** and *S*-*E*-**M2** (Chiralcel OD-H, *n*-heptane/2-propanol 99.5:0.5, 0.5 mL/min).

<Chromatogram>

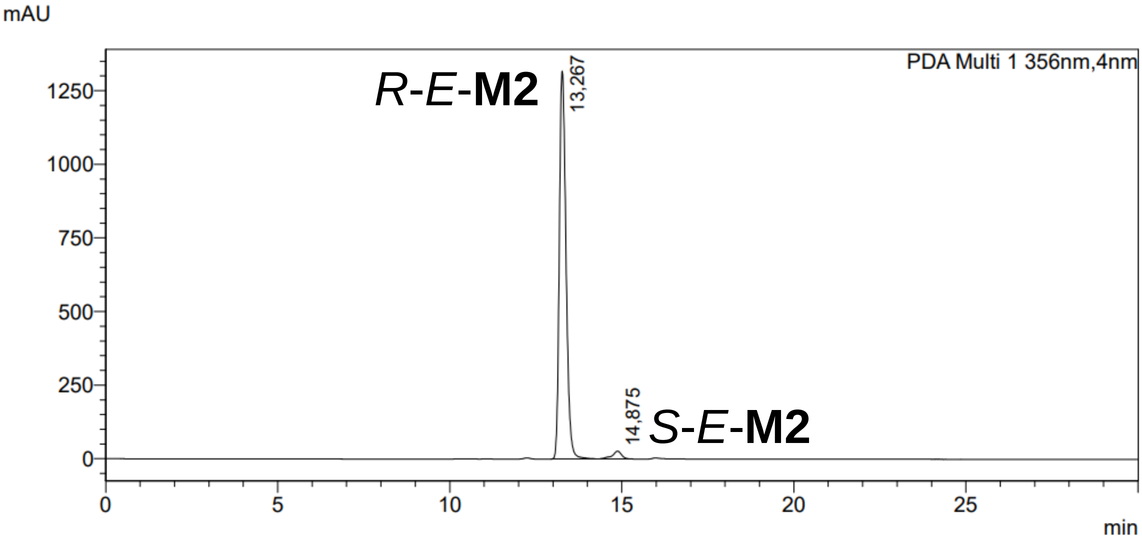

<Peak Table>

PDA Ch1 356nm

Peak#	Ret. Time	Area%
1	13,267	97,225
2	14,875	2,775
Total		100,000

HPLC chromatogram of *R*-*E*-**M2** (Chiralcel OD-H, *n*-heptane/2-propanol 99.5:0.5, 0.5 mL/min).

<Chromatogram>

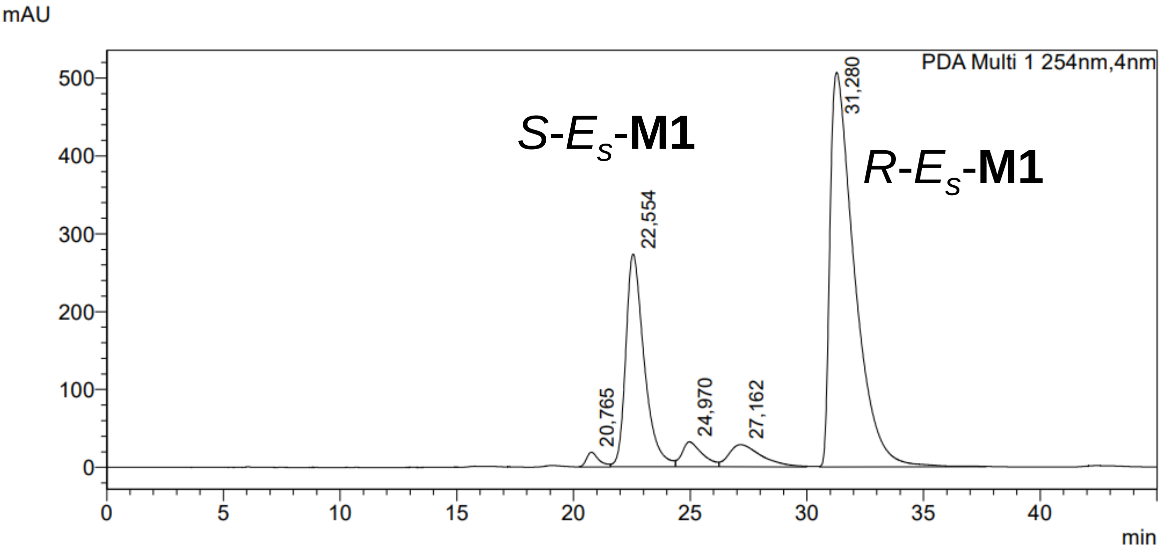

<Peak Table>

PDA Ch1 254nm

Peak#	Ret. Time	Area%
1	20,765	1,229
2	22,554	26,122
3	24,970	3,355
4	27,162	4,787
5	31,280	64,507
Total		100,000

HPLC chromatogram of *S*-*E*-**M2** (Chiralcel OD-H, *n*-heptane/2-propanol 99.5:0.5, 0.5 mL/min).

10. NMR spectra of new compounds

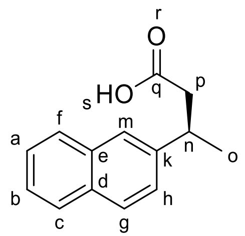

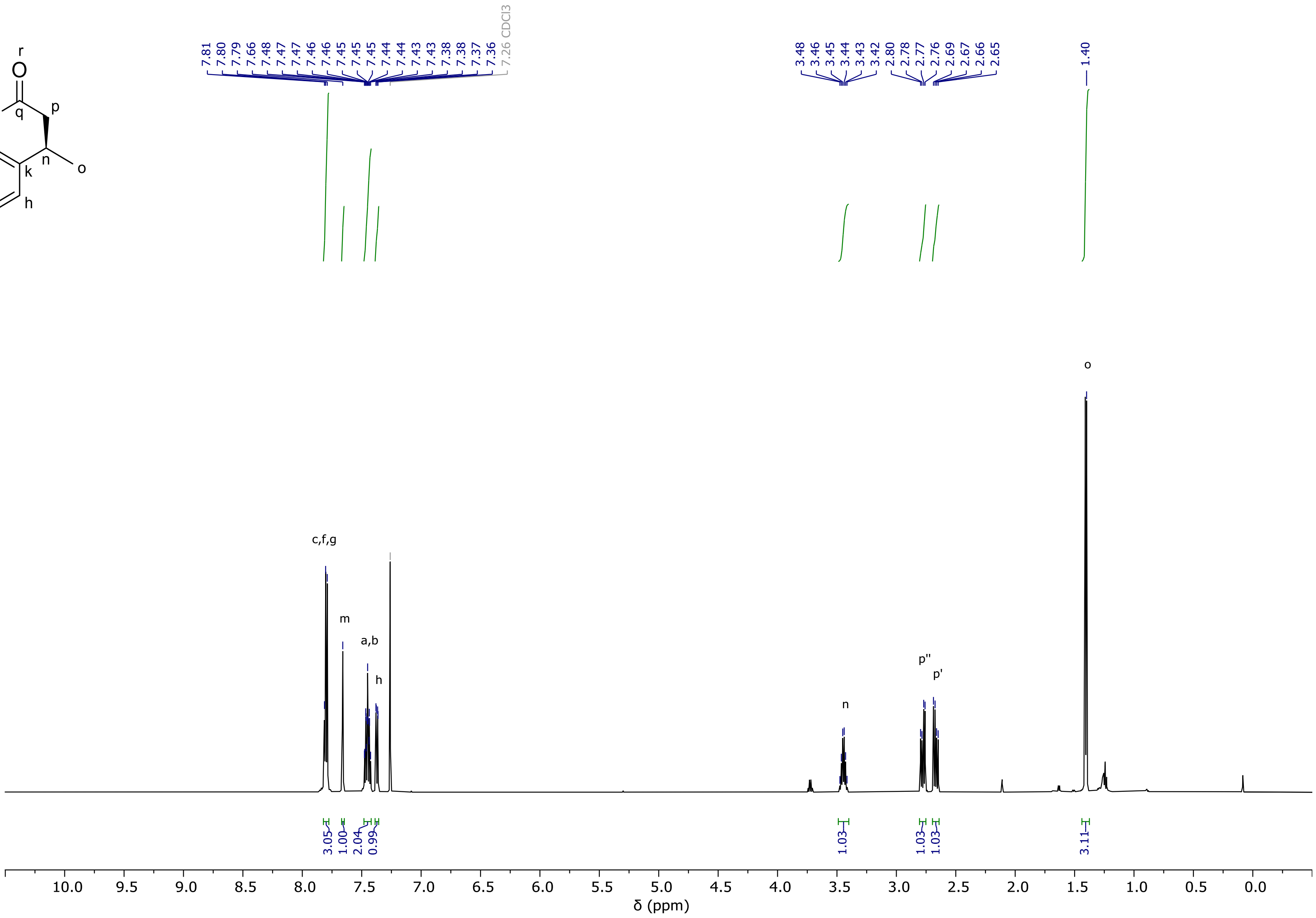

^{1}H-NMR of compound *R*-**4** (600 MHz, $CDCl_3$, 25 °C).

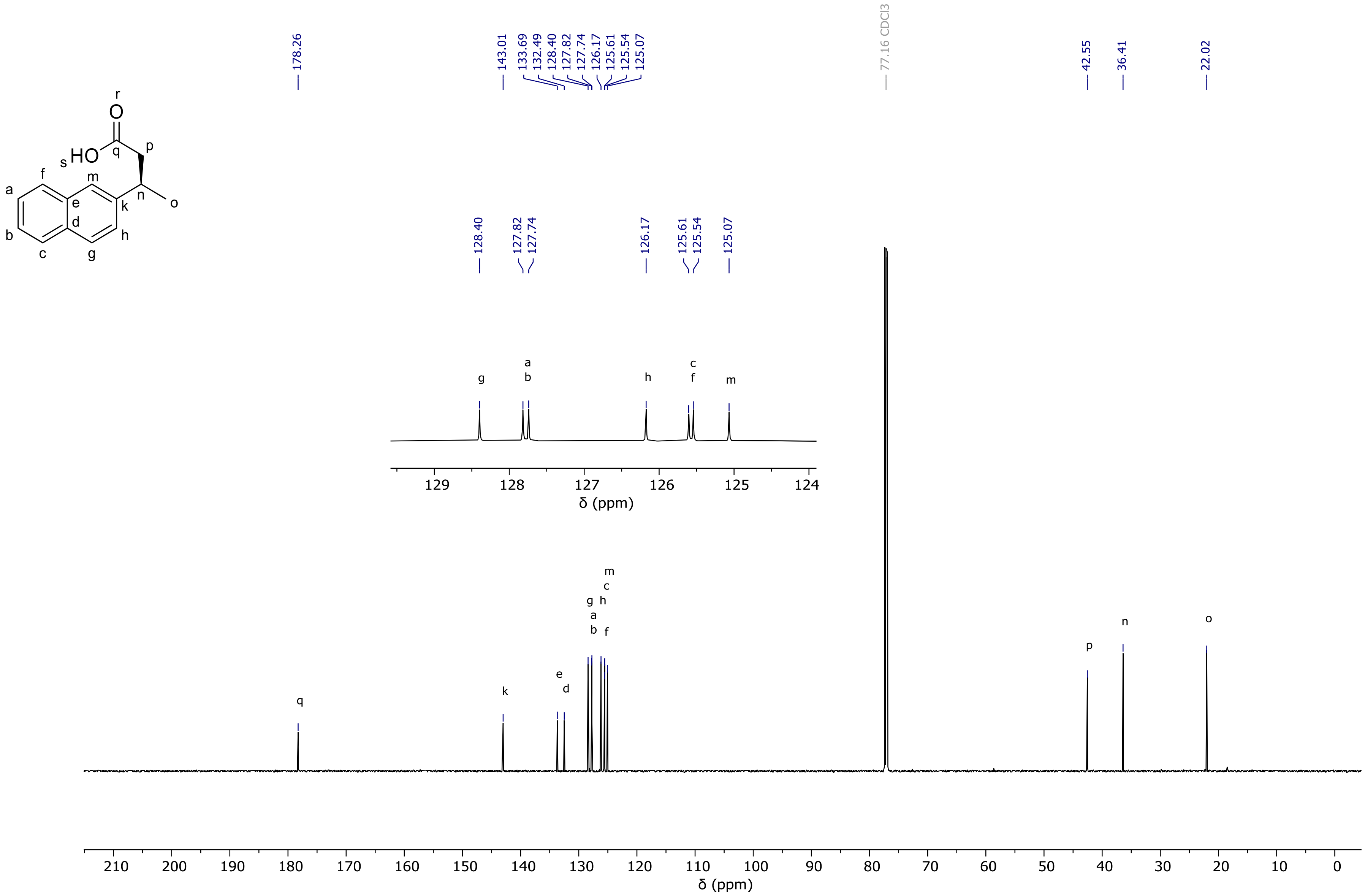

$^{13}C\{^{1}H\}$-NMR of compound *R*-**4** (151 MHz, $CDCl_3$, 25 °C).

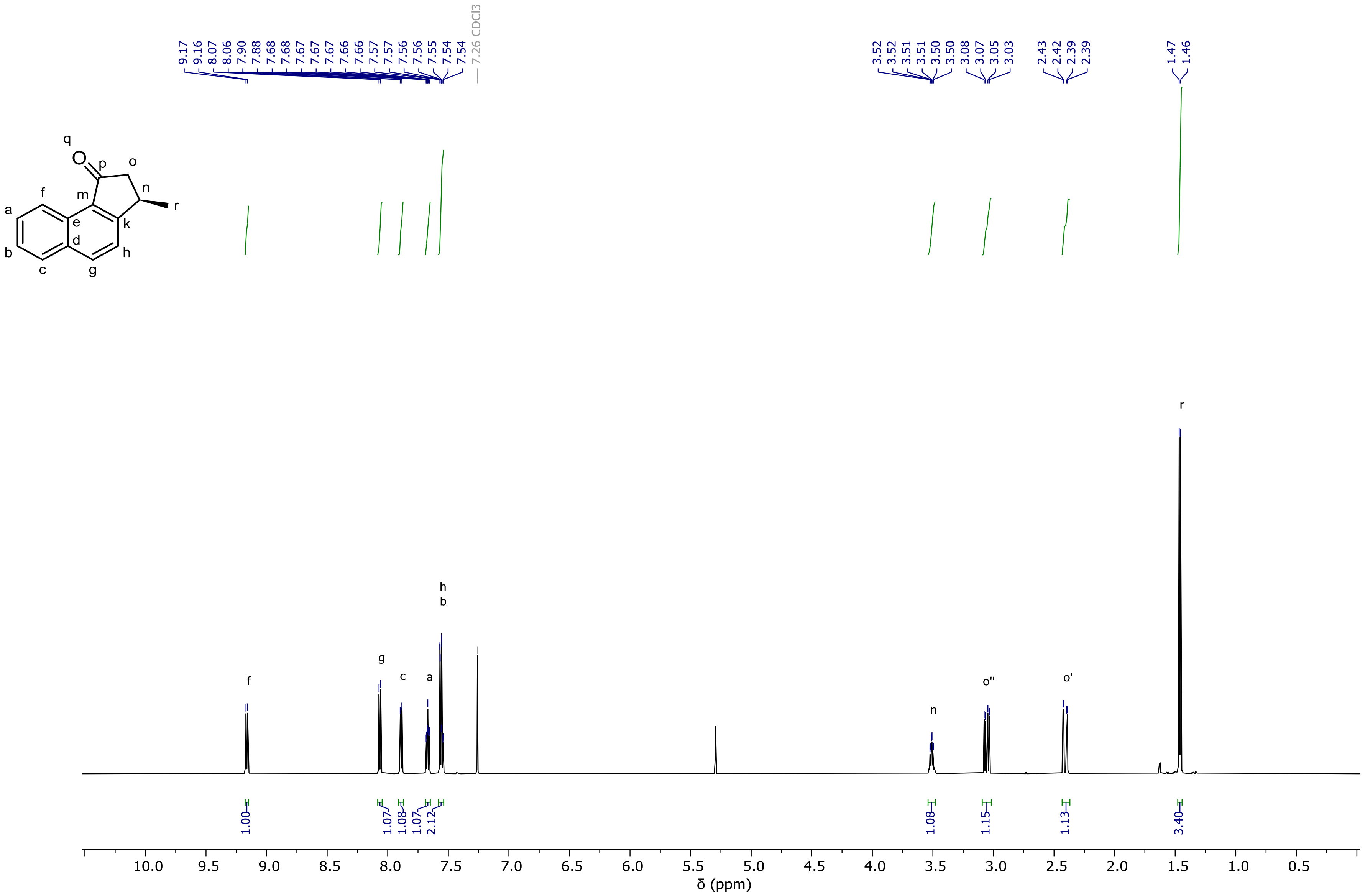

^{1}H-NMR of compound *R*-**5** (600 MHz, $CDCl_3$, 25 °C).

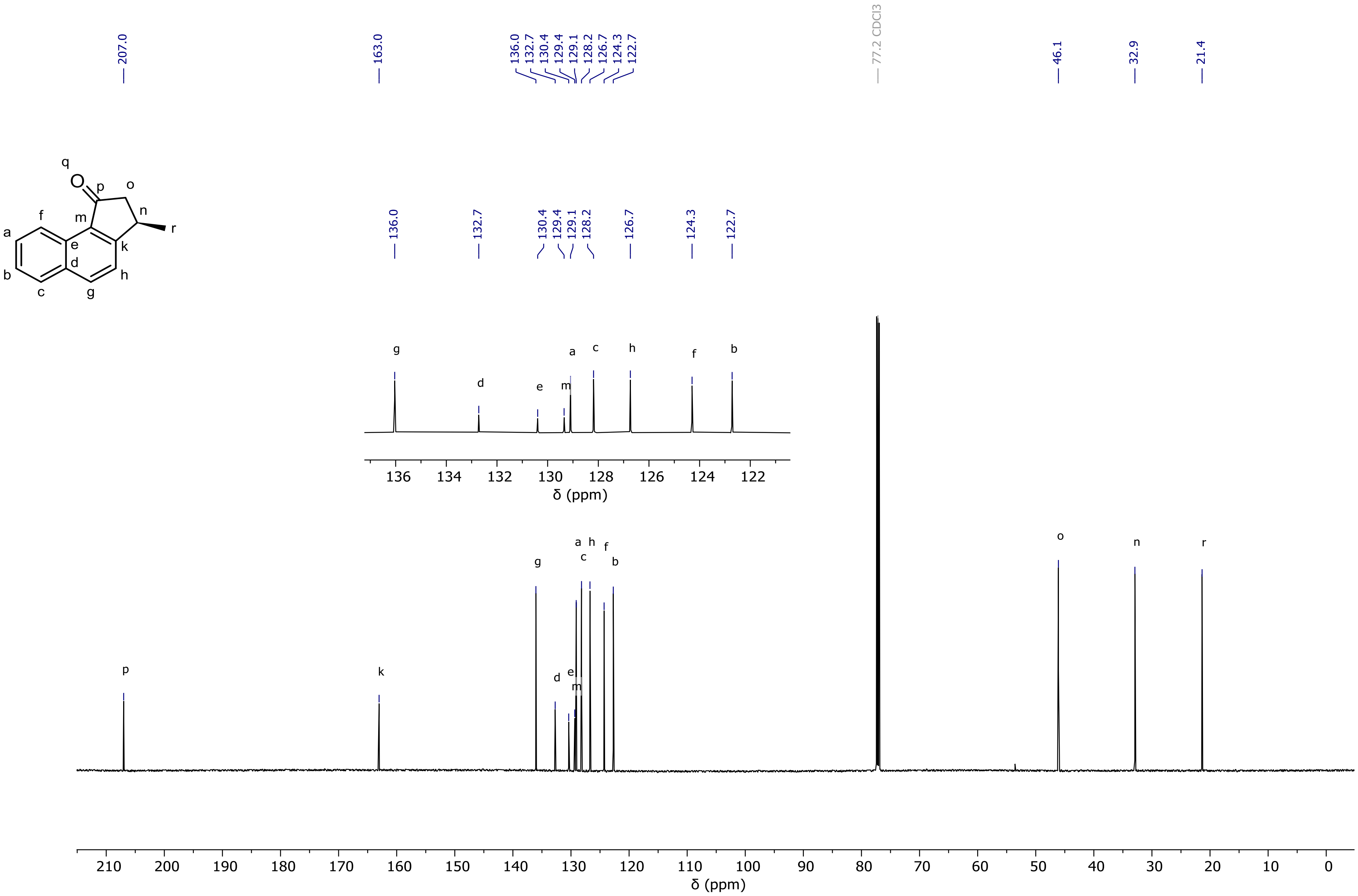

$^{13}C\{^{1}H\}$-NMR of compound *R*-**5** (151 MHz, $CDCl_3$, 25 °C).

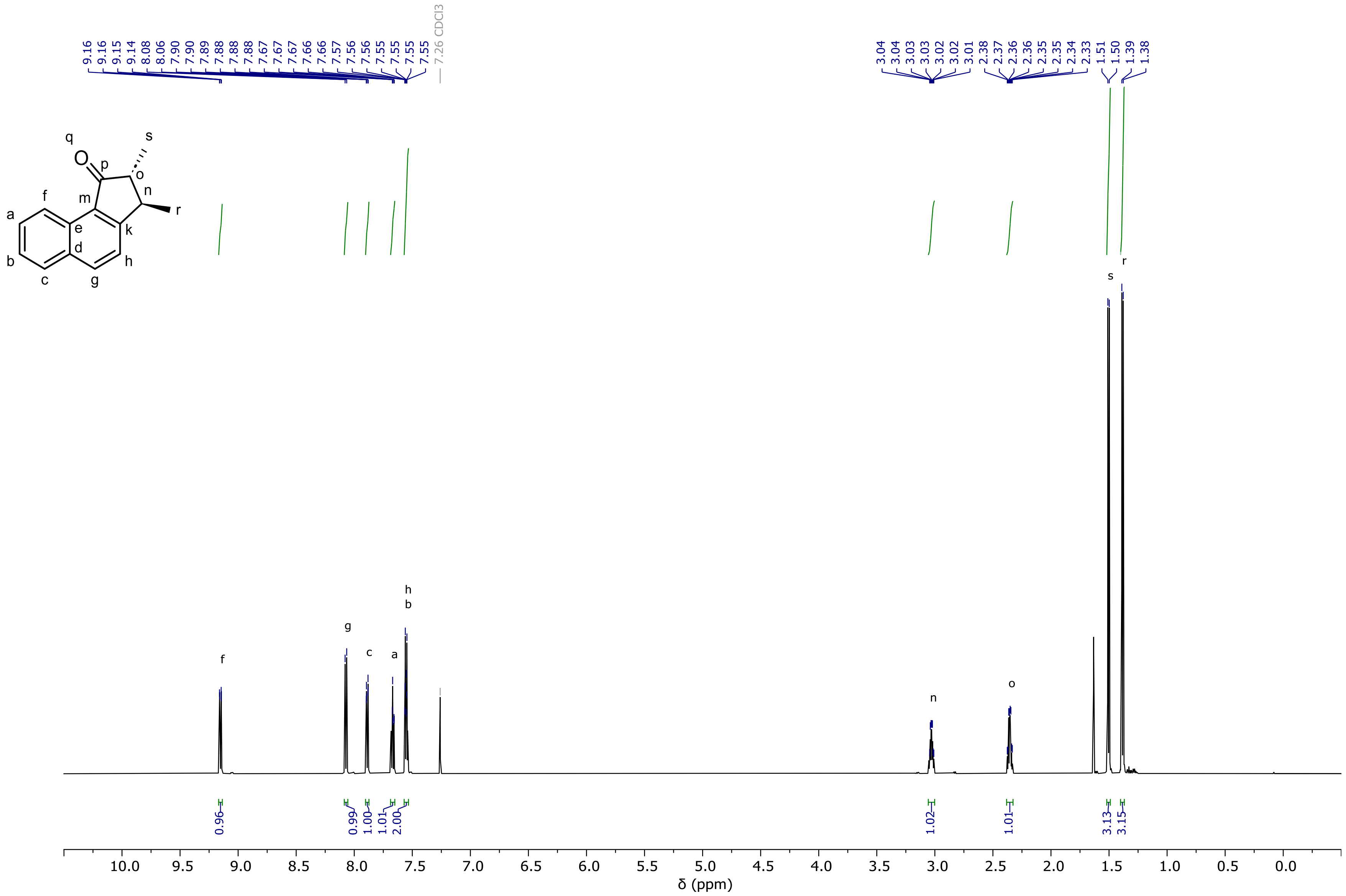

^{1}H-NMR of compound *R*-**6** (600 MHz, $CDCl_3$, 25 °C).

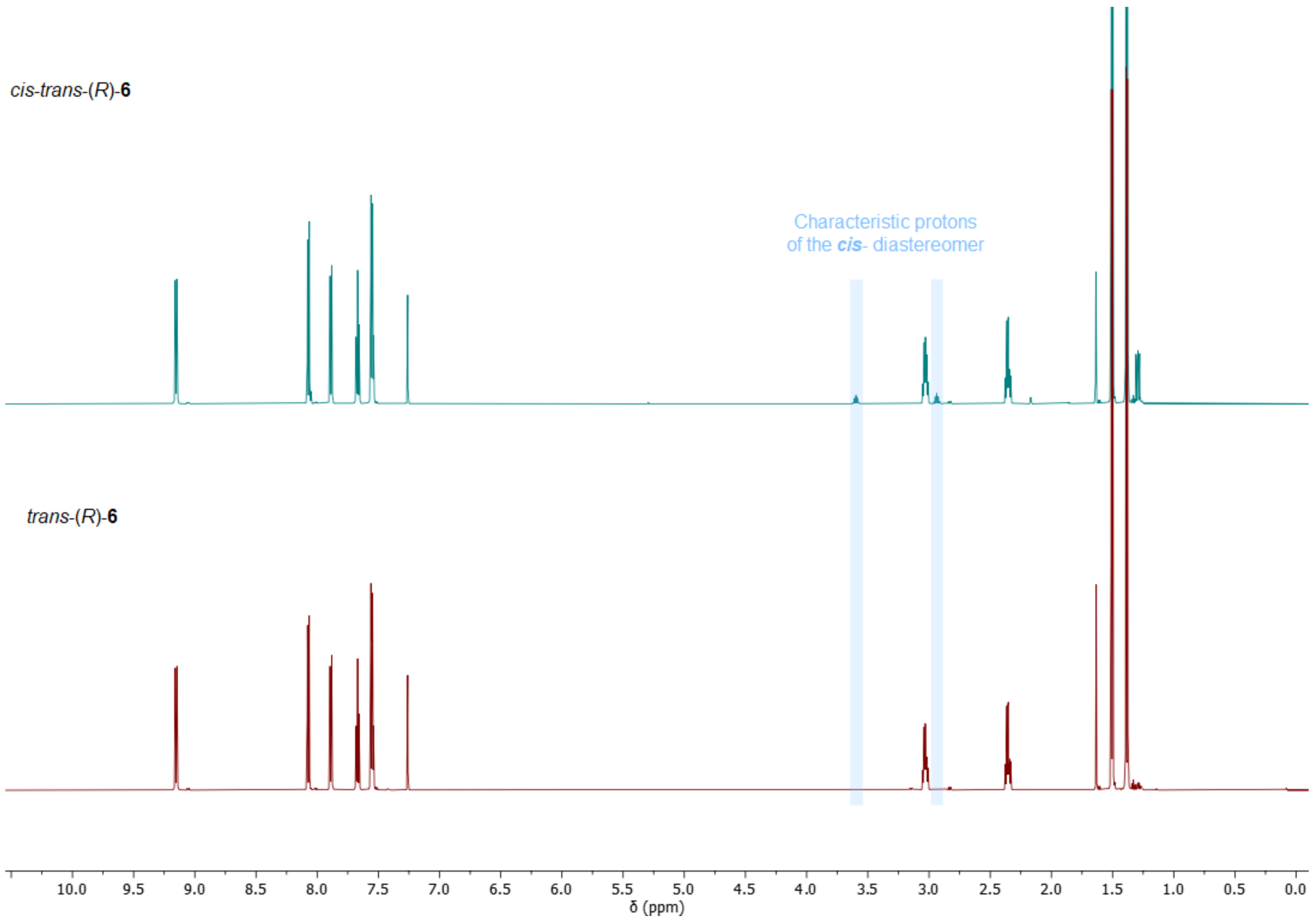

^{1}H-NMR of compound *R*-**6** (bottom) and a partially purify mixture of *cis*- and *trans*- diastereomers[2] showing that *R*-**6** is obtained diastereopure after column chromatography (600 MHz, $CDCl_3$, 25 °C).

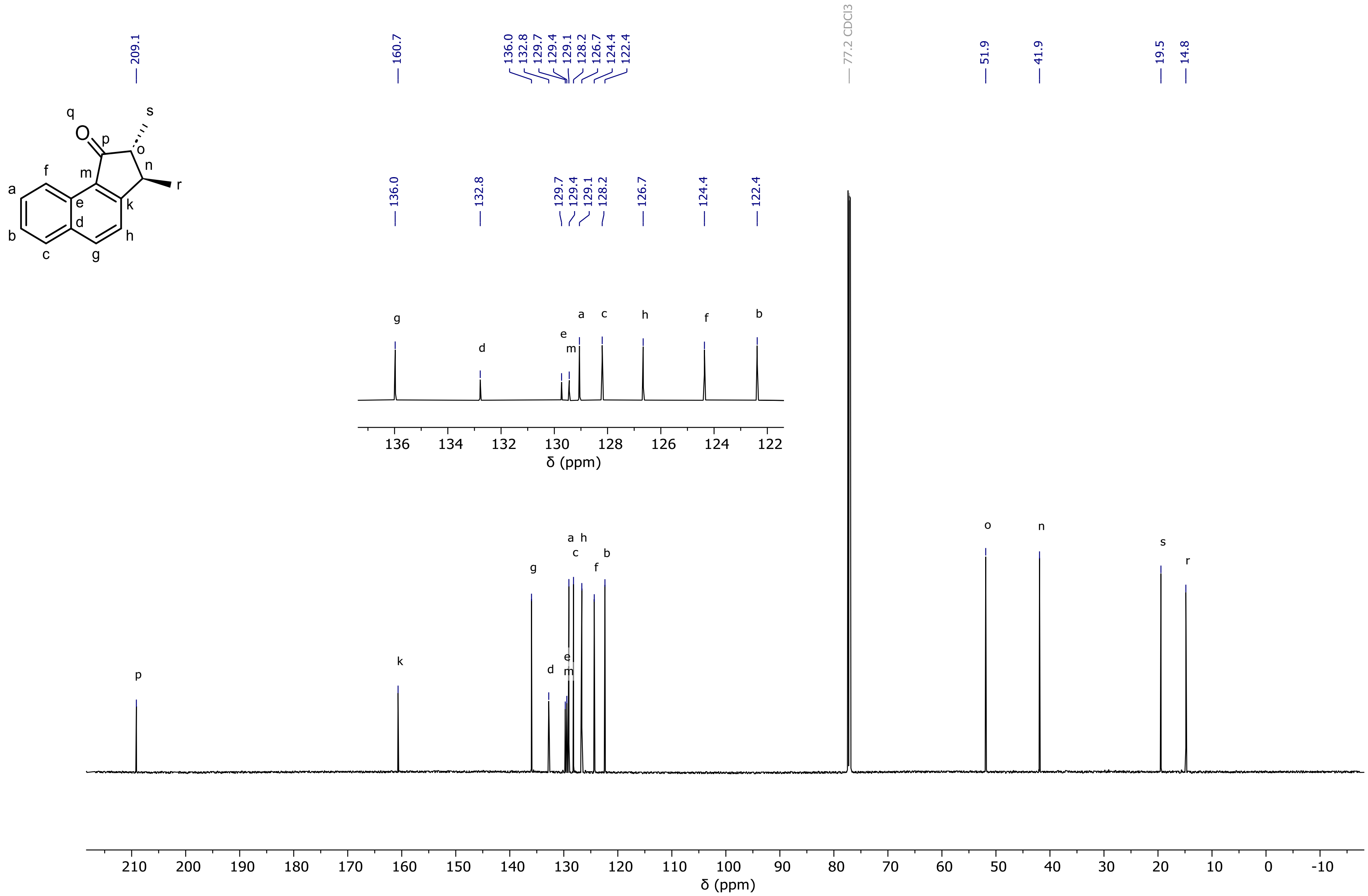

^{13}C{^{1}H}-NMR of compound *R*-**6** (151 MHz, $CDCl_3$, 25 °C).

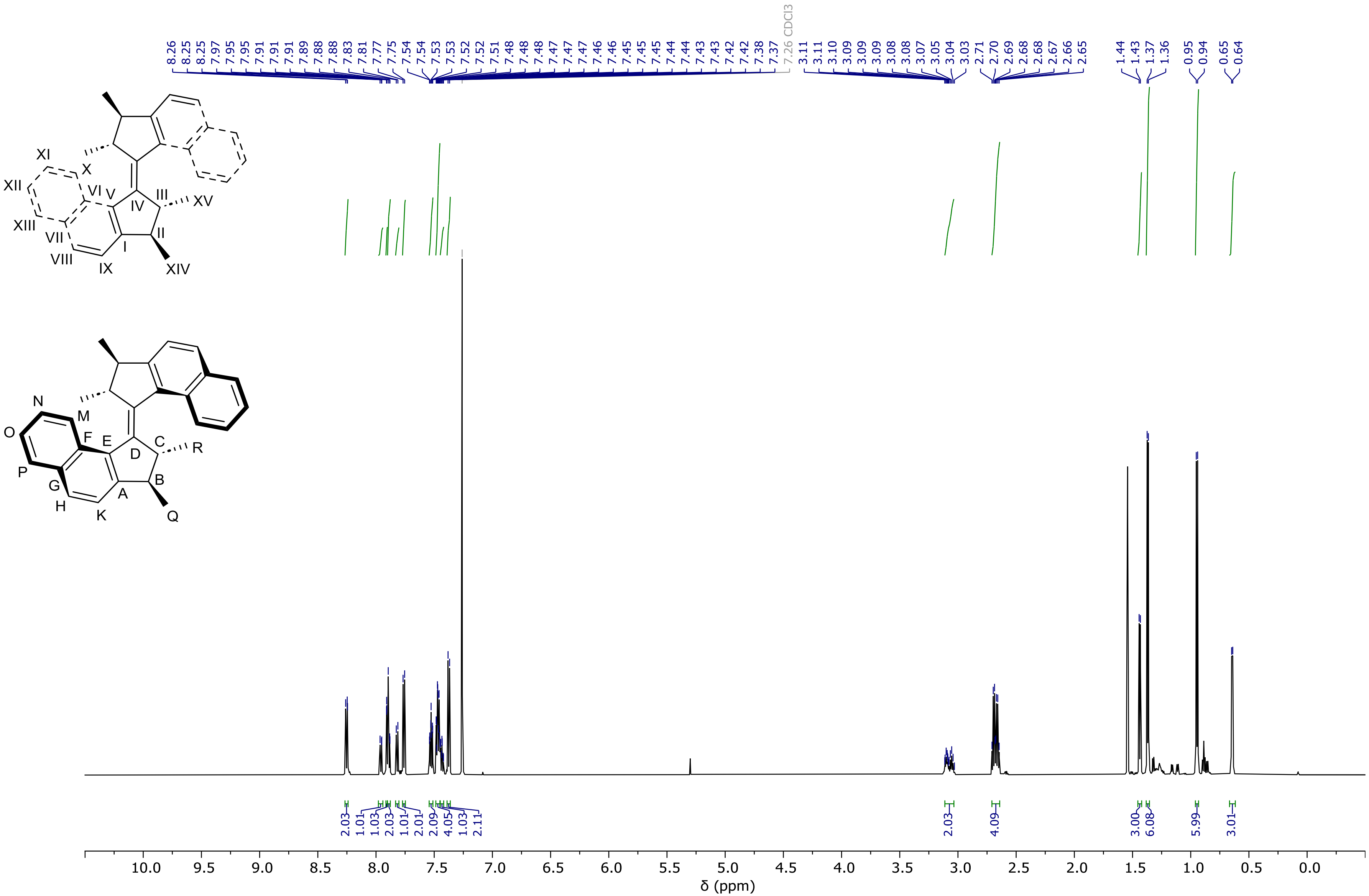

^{1}H-NMR of a mixture of *R*-E_s-**M1** and *R*-E_{ms}-**M1** (600 MHz, $CDCl_3$, 25 °C).

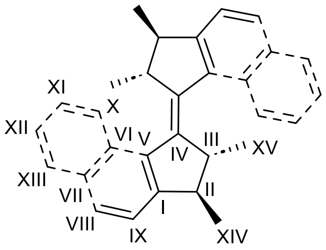

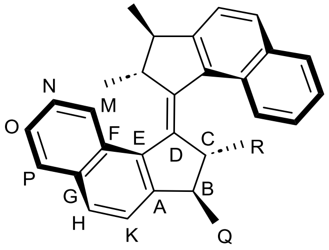

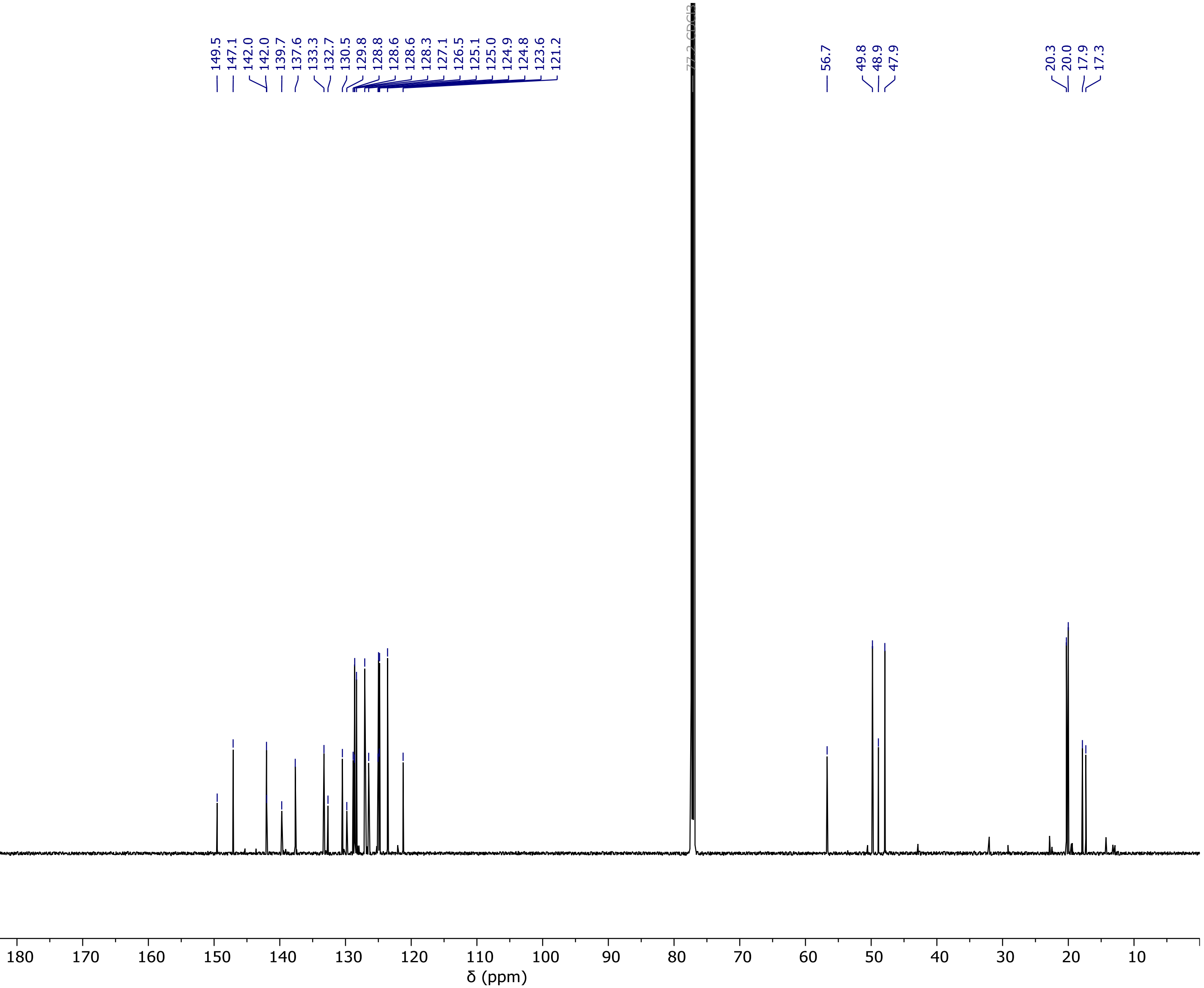

$^{13}C\{^{1}H\}$-NMR of a mixture of *R*-*E*$_{s}$-**M1** and *R*-*E*$_{ms}$-**M1** (151 MHz, $CDCl_3$, 25 °C).

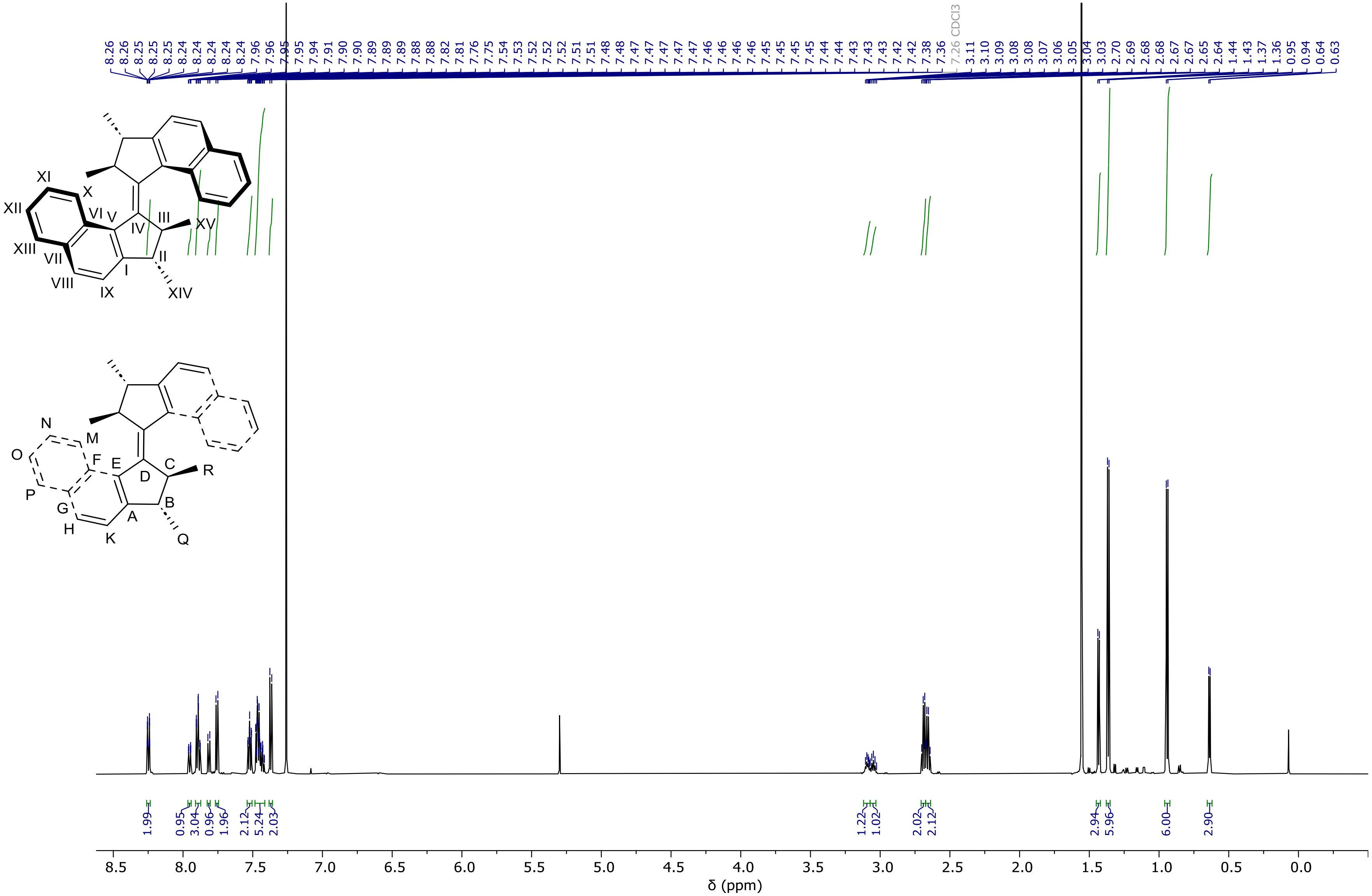

^{1}H-NMR of a mixture of *S*-*E*$_s$-**M1** and *S*-*E*$_{ms}$-**M1** (600 MHz, $CDCl_3$, 25 °C).

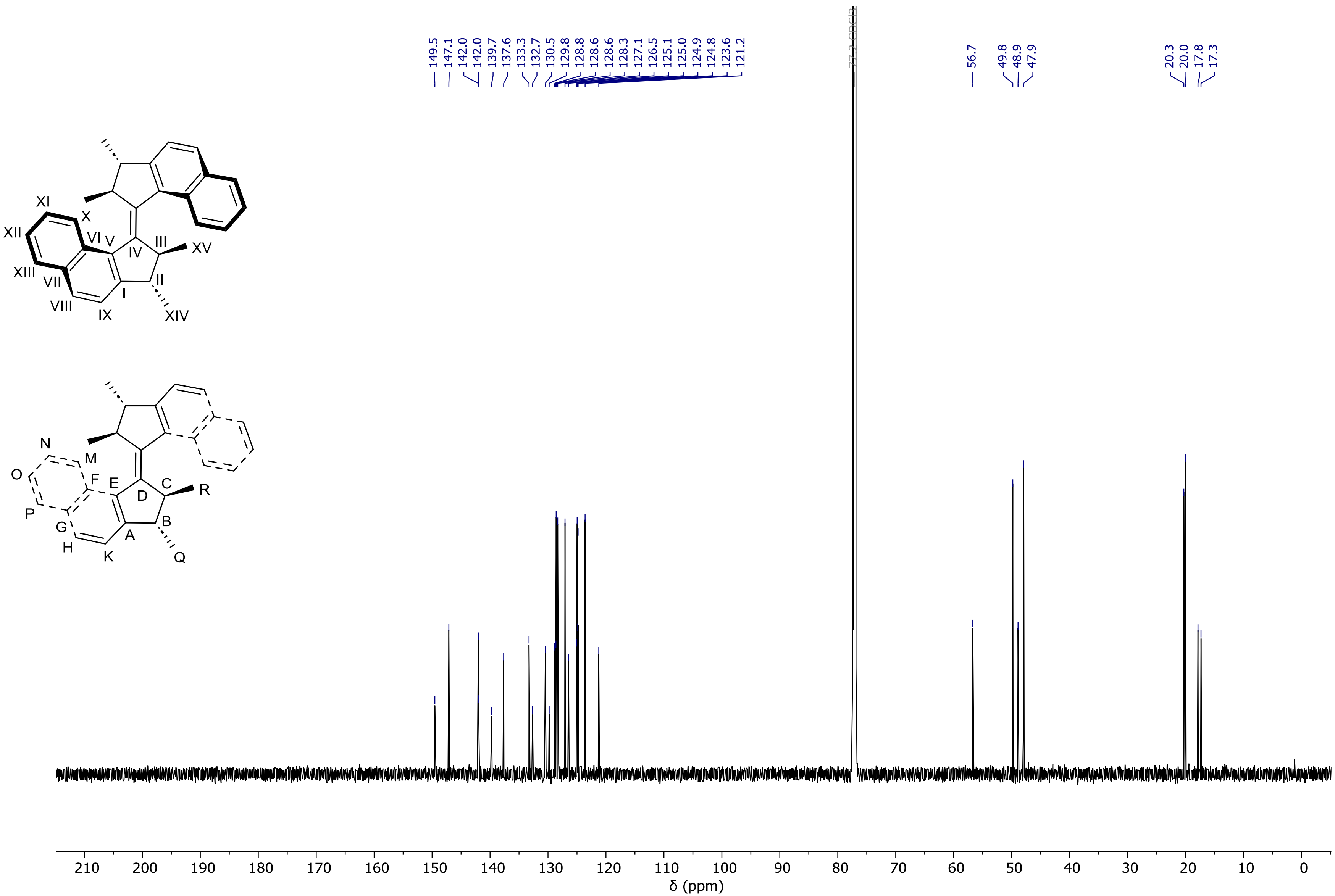

$^{13}C\{^{1}H\}$-NMR of a mixture of *S*-E_s-**M1** and *S*-E_{ms}-**M1** (151 MHz, $CDCl_3$, 25 °C).

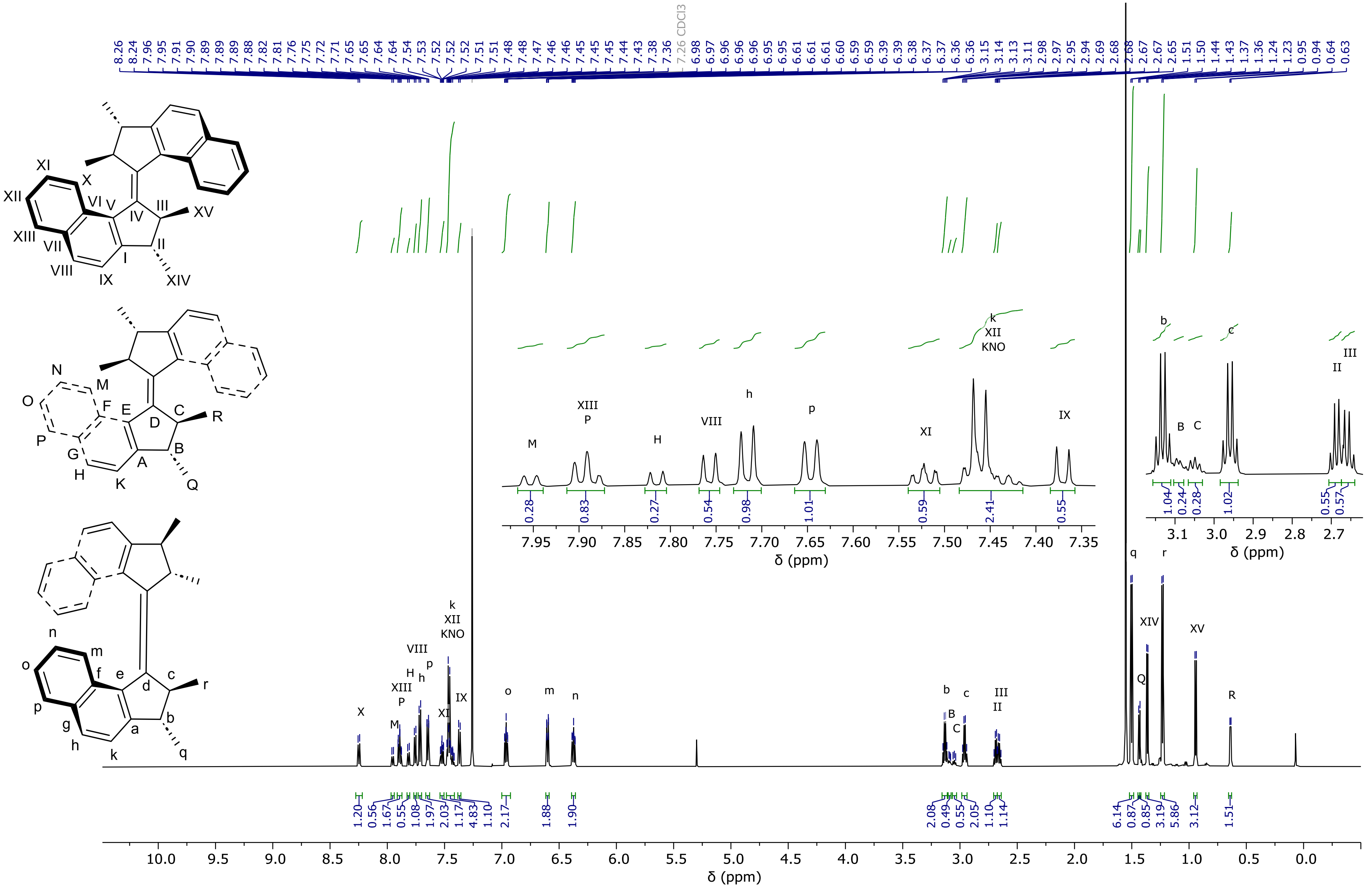

^{1}H-NMR of a mixture of *S*-E_s-**M1**, *S*-E_{ms}-**M1** and *S*-*Z*-**M1** (600 MHz, $CDCl_3$, 25 °C).

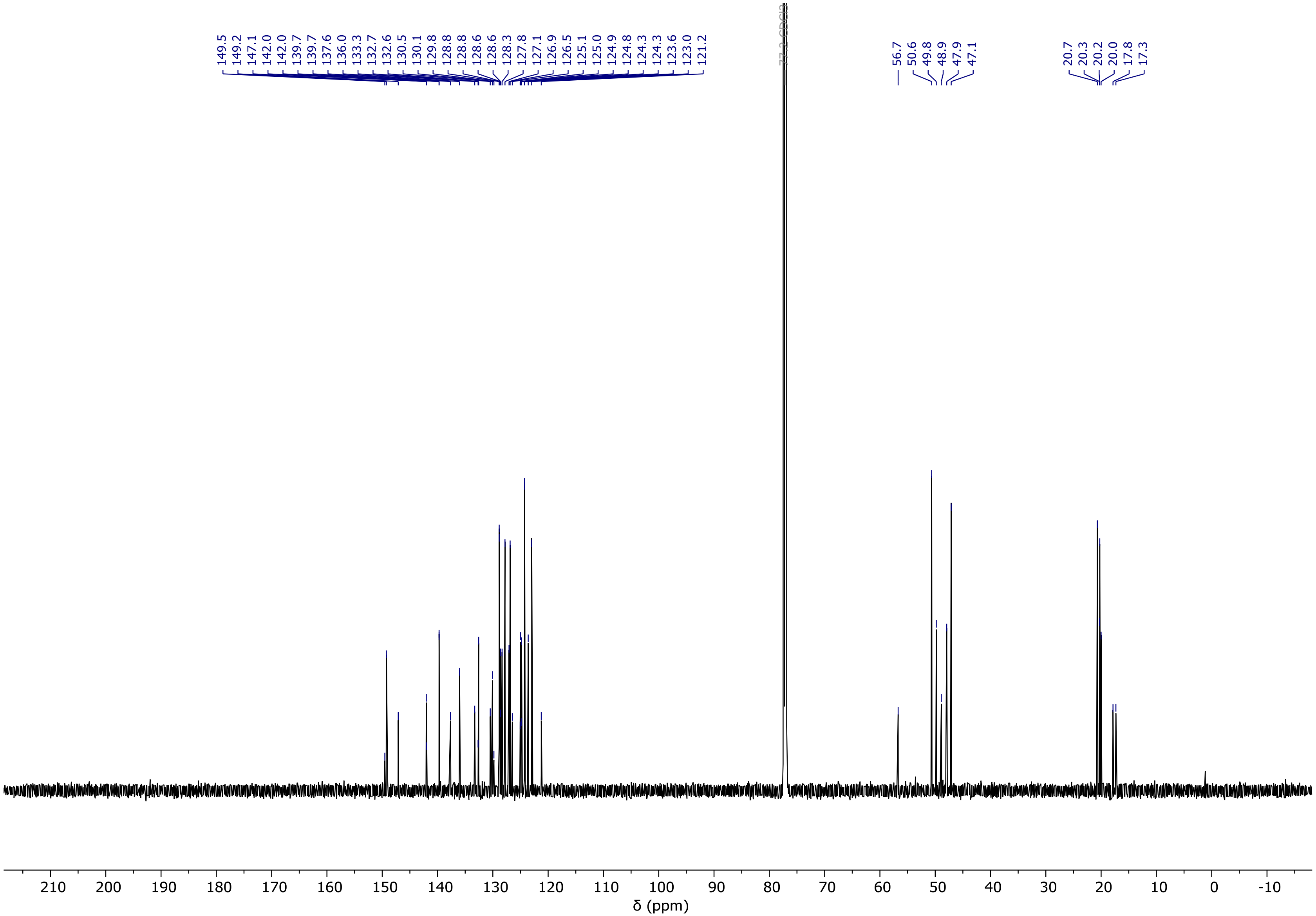

$^{13}C\{^1H\}$-NMR (complete spectrum) of a mixture of *S*-*E*$_s$-**M1**, *S*-*E*$_{ms}$-**M1** and *S*-*Z*-**M1** (151 MHz, $CDCl_3$, 25 °C).

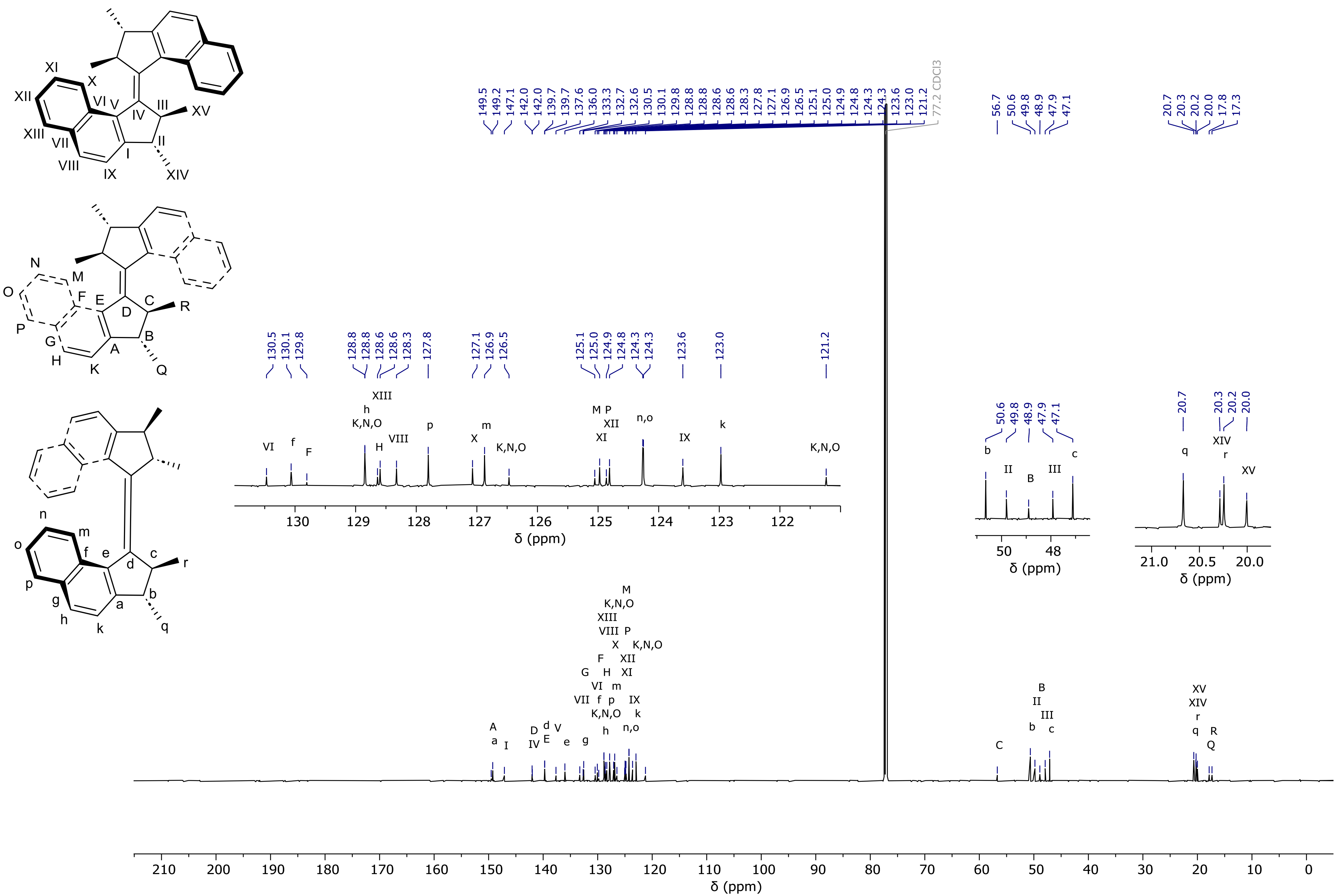

^{13}C{^{1}H}-NMR (with zoomed views) of a mixture of *S*-*E*$_{s}$-**M1**, *S*-*E*$_{ms}$-**M1** and *S*-*Z*-**M1** (151 MHz, CDCl$_3$, 25 °C).

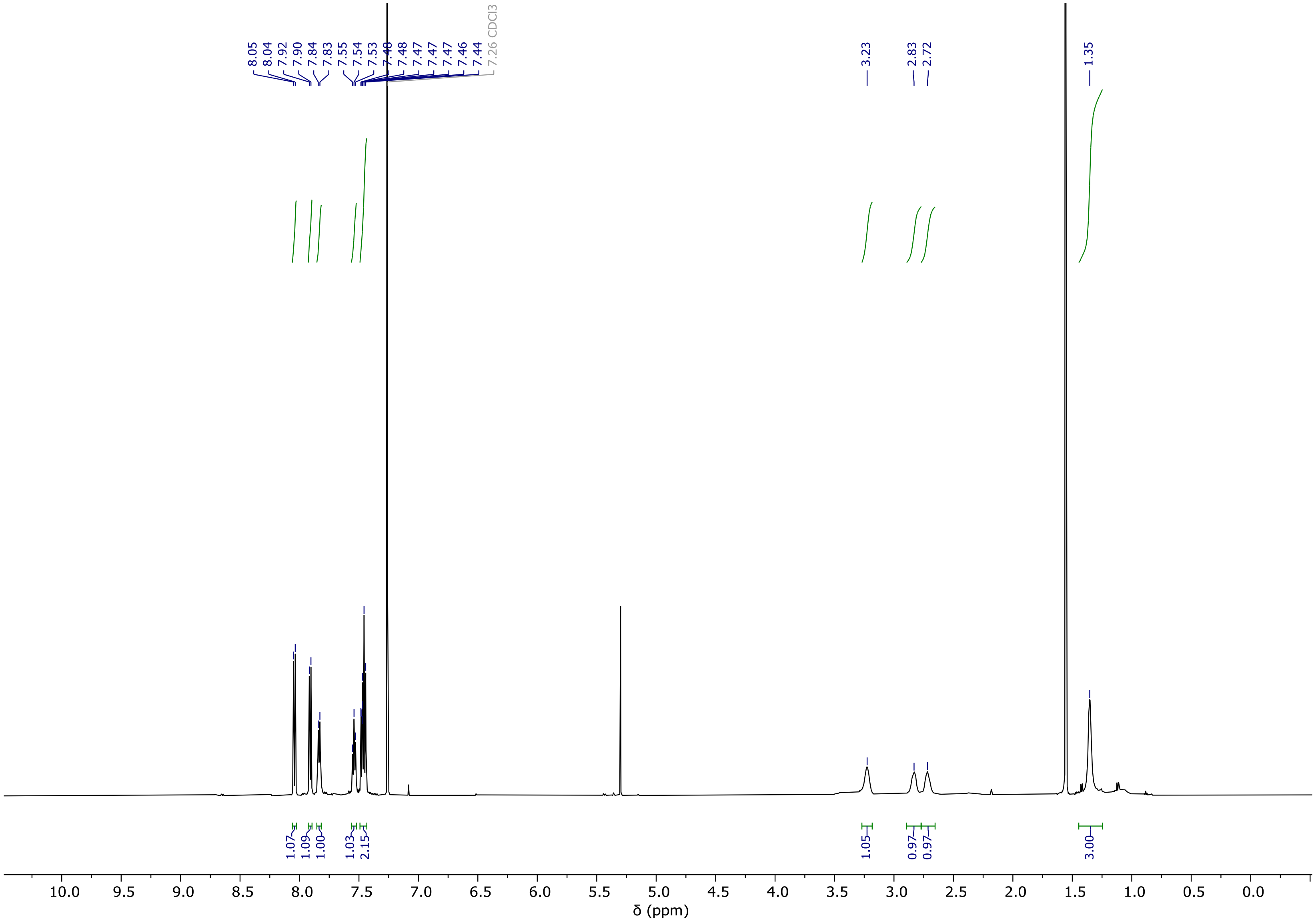

^{1}H-NMR of compound *S*-*E*-**M2** (600 MHz, $CDCl_3$, 25 °C).

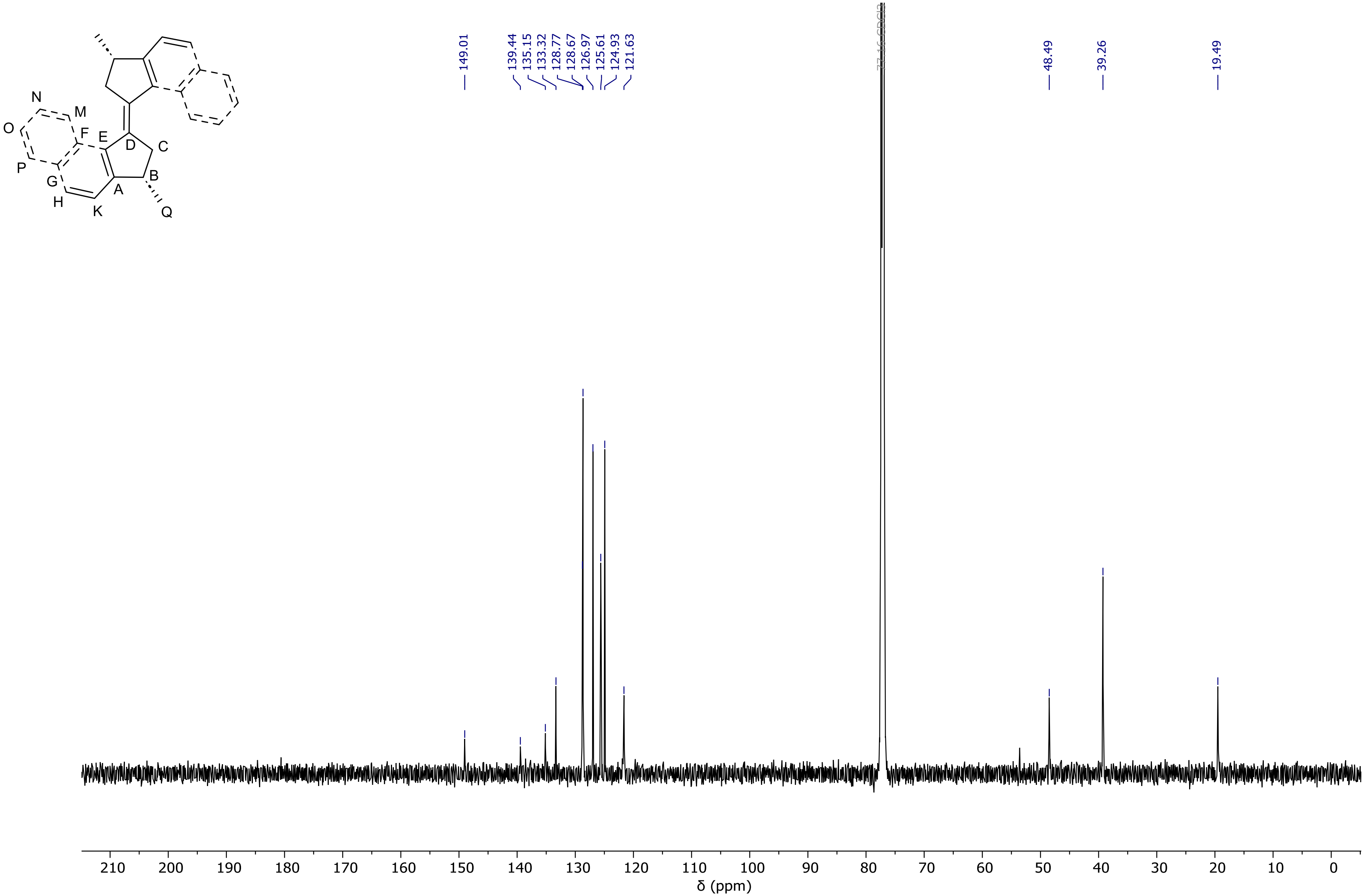

$^{13}C\{^{1}H\}$-NMR of compound *S*-*E*-**M2** (151 MHz, $CDCl_3$, 25 °C).

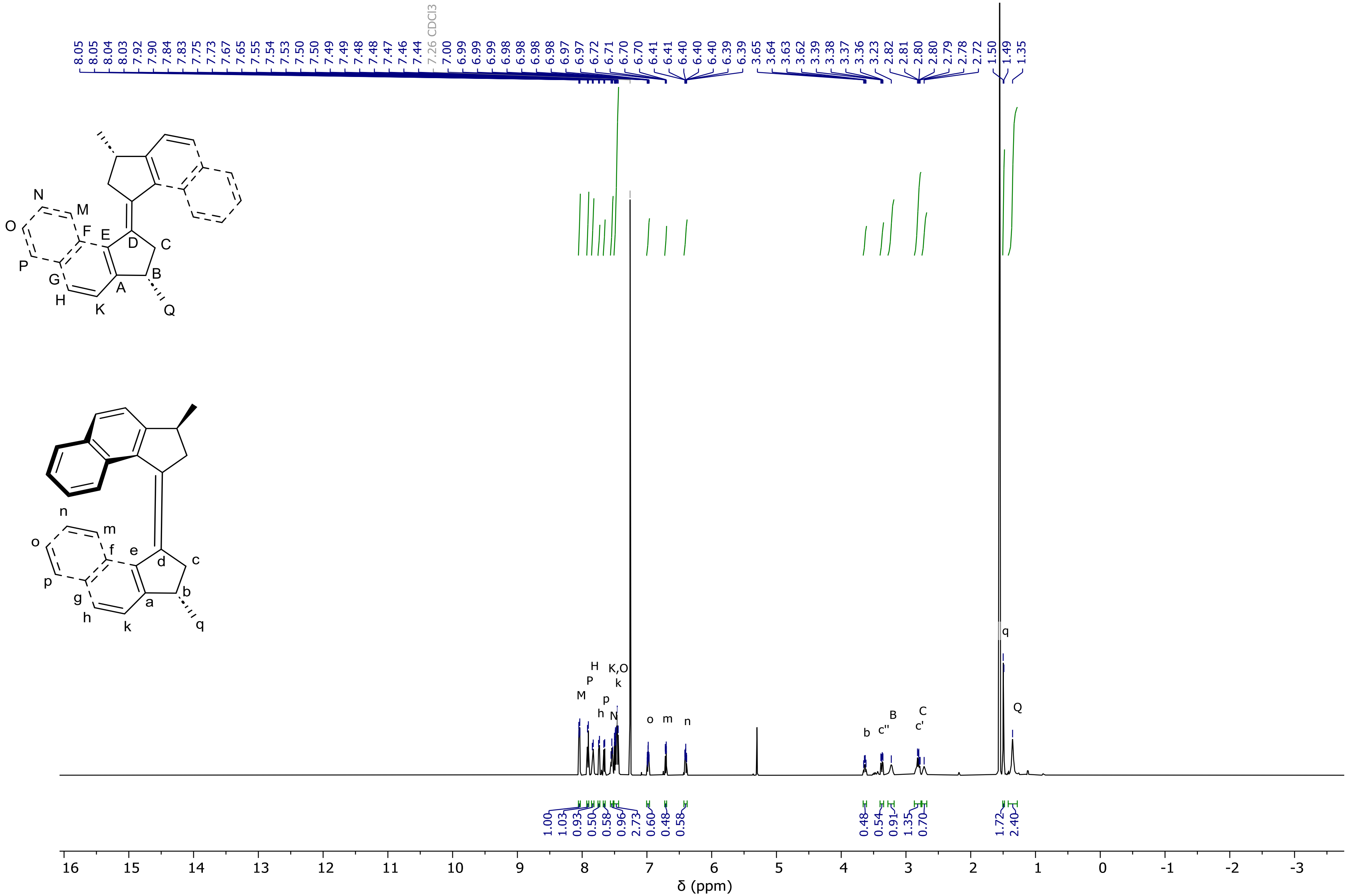

^{1}H-NMR of a mixture of *S*-*E*-**M2** and *S*-*Z*-**M2** (600 MHz, $CDCl_3$, 25 °C).

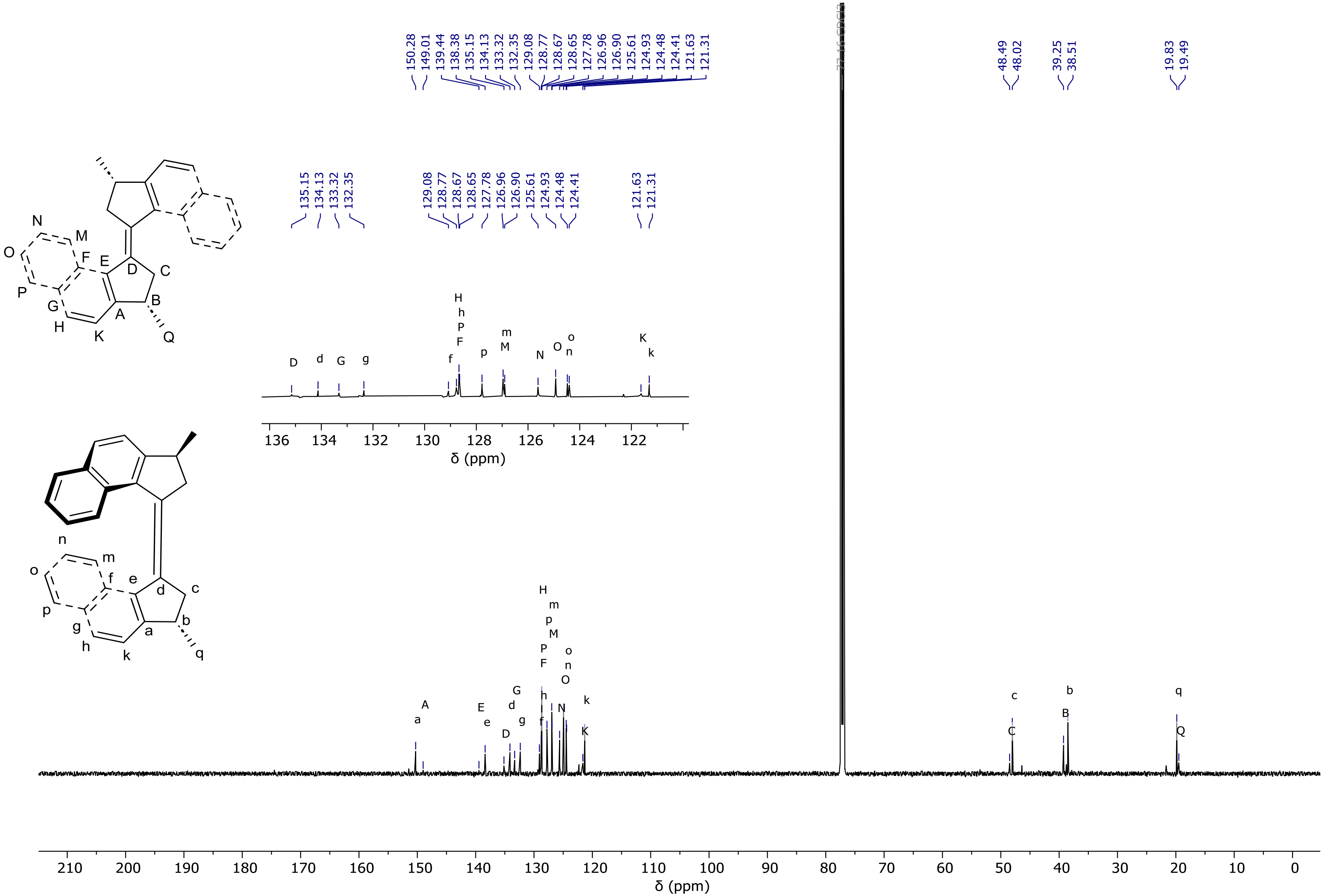

$^{13}C\{^{1}H\}$-NMR of a mixture of *S*-*E*-**M2** and *S*-*Z*-**M2** (151 MHz, $CDCl_3$, 25 °C).

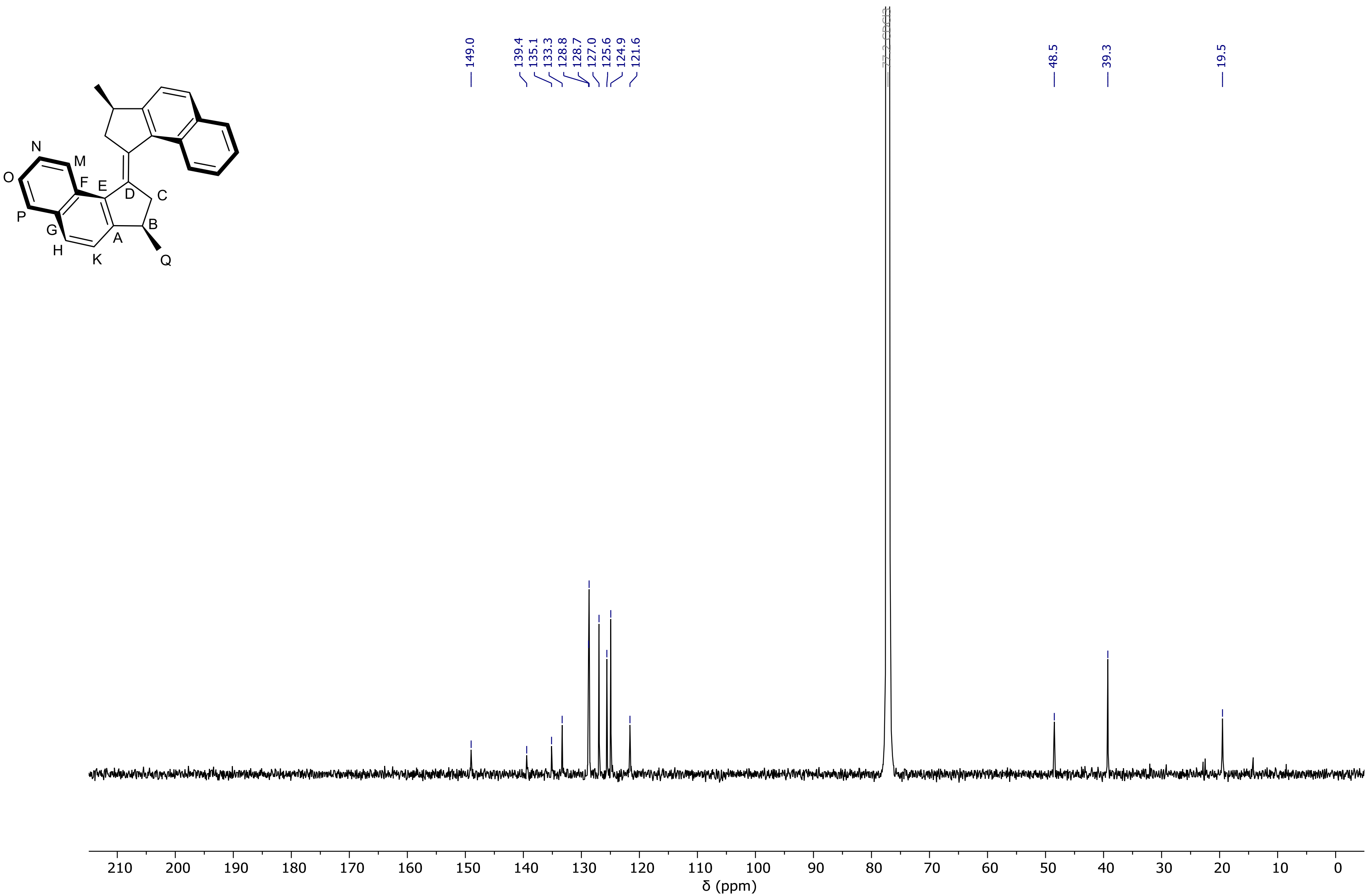

^{1}H-NMR of compound *R*-*E*-**M2** (600 MHz, $CDCl_3$, 25 °C).

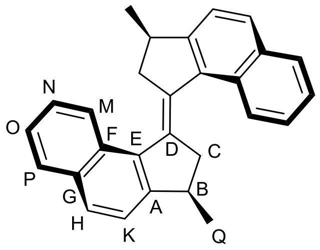

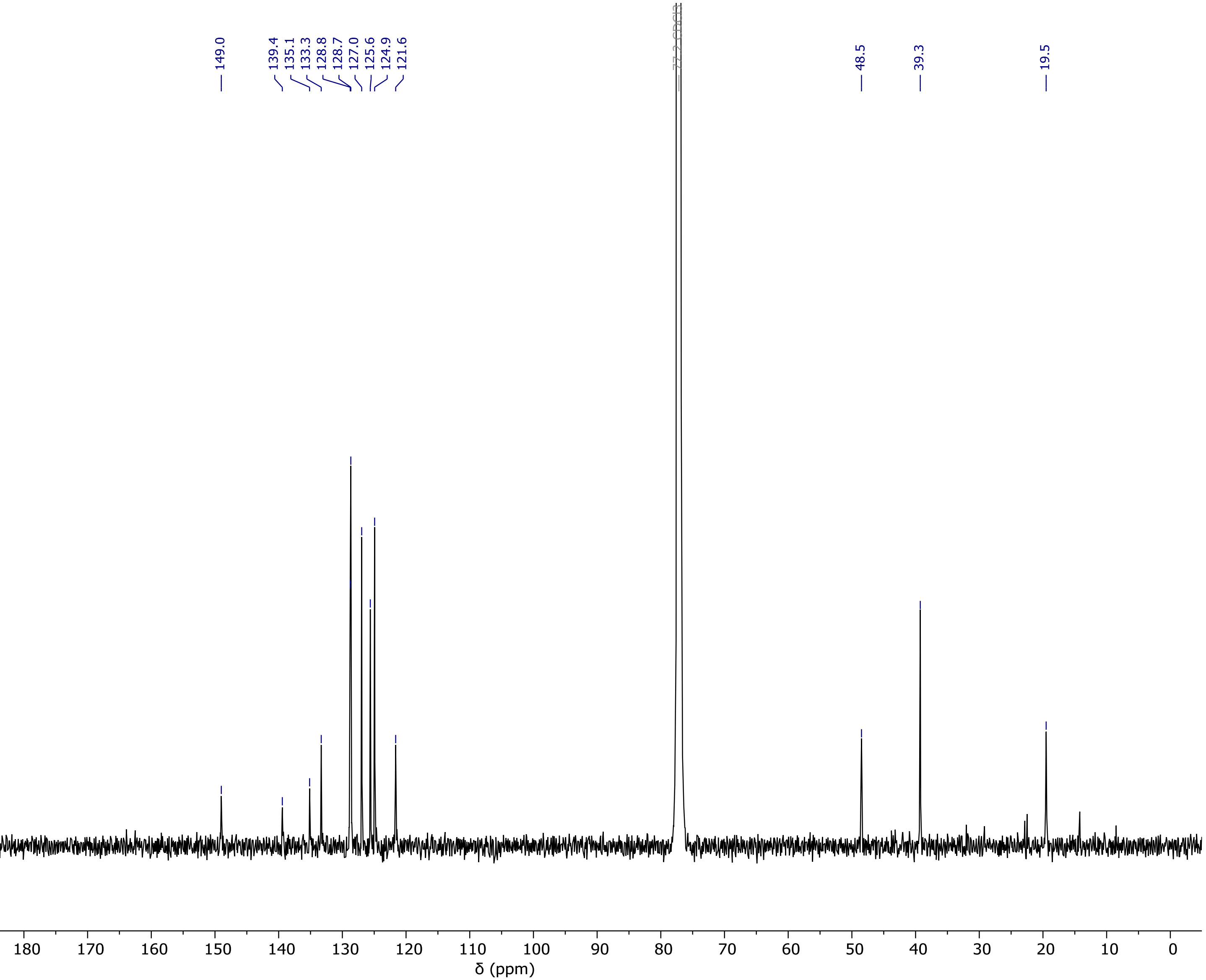

^{13}C{^{1}H}-NMR of compound *R*-*E*-**M2** (151 MHz, $CDCl_3$, 25 °C).

11. References

[1] A. Fujii, S. Hashiguchi, N. Uematsu, T. Ikariya, R. Noyori, *J. Am. Chem. Soc.* **1996**, *118*, 2521–2522.
[2] T. Van Leeuwen, W. Danowski, E. Otten, S. J. Wezenberg, B. L. Feringa, *J. Org. Chem.* **2017**, *82*, 5027–5033.
[3] F. Neese, F. Wennmohs, U. Becker, C. Riplinger, *J. Chem. Phys.* **2020**, *152*, 224108.
[4] S. Grimme, A. Hansen, S. Ehlert, J.-M. Mewes, *J. Chem. Phys.* **2021**, *154*, 064103.
[5] V. Barone, M. Cossi, *J. Phys. Chem. A* **1998**, *102*, 1995–2001.
[6] G. M. Sheldrick, *Acta Cryst. A* **2015**, *71*, 3–8.
[7] G. M. Sheldrick, *Acta Crys. A* **2008**, *64*, 112–122.
[8] O. V. Dolomanov, L. J. Bourhis, R. J. Gildea, J. a. K. Howard, H. Puschmann, *J. Appl. Cryst.* **2009**, *42*, 339–341.
[9] N. A. Farrow, O. Zhang, J. D. Forman-Kay, L. E. Kay, *J. Biomol. NMR* **1994**, *4*, 727–734.
